# Supplementary material for: Evaluating the use of blood pressure polygenic risk scores across race/ethnic background groups
Source: Nat Commun. 2023 Jun 2;14:3202. doi: 10.1038/s41467-023-38990-9 (PMC10238525; doi:10.1038/s41467-023-38990-9)
Supplement: Supplementary file 1 — Supplementary Information [file 41467_2023_38990_MOESM1_ESM.pdf]

# Evaluating the use of blood pressure polygenic risk scores across race/ethnic background groups:

## Supplementary Information

Kurniansyah et al.

Supplementary Note 1: supplementary methods ....... 4
UK Biobank methods ....... 4
Mass General Brigham Biobank methods ....... 6
Global genetic ancestry inference in TOPMed ....... 9
Secondary analysis of scaling and scaling + matching to address PRS distribution differences across datasets ....... 10
All of Us Methods ....... 11
Supplementary Tables ....... 15
Supplementary Table 1: Characteristics of TOPMed-BP study participants. ....... 15
Supplementary Table 2: Characteristics of Black participants from UK Biobank. ....... 16
Supplementary Table 3: Characteristics of MGB Biobank study participants. ....... 17
Supplementary Table 4: Characteristics of All of Us participants. ....... 18
Supplementary Table 5: Number of TOPMed + UKB individuals participating in each of the PRS analyses. ....... 19
Supplementary Table 6: PRS-CSx2 means and SDs in the TOPMed-BP dataset ....... 20
Supplementary Table 7 : Means and SDs of the weighted PRS-CSx2 sum in the TOPMed-BP dataset ....... 20
Supplementary Table 8 : MGB Biobank-trained PRS summation weights for PRS-CSx2 ....... 21
Supplementary Table 9: Standard concept names used to define clinical outcomes in All of Us ....... 21
Supplementary Figures ....... 22
Supplementary Figure 1: Estimated phenotypic and residual variances of SBP by study and diversity background. ....... 23
Supplementary Figure 2: Estimated phenotypic and residual variances of DBP by study and diversity background. ....... 24
Supplementary Figure 3: Estimated global proportions of continental ancestries in TOPMed-BP participants. ....... 25
Supplementary Figure 4: Comparison of variance explained according to weighting method: Background-specific versus Multi-ethnic PRS combination weights derived from MGB Biobank. ....... 26

Supplementary Figure 5: Comparison of variance explained according to LD reference panel choice: GWAS-matched, target-matched, or TOPMed LD-panels. 27

Supplementary Figure 6: Distribution of UKBB+ICBP LDPRed2 SBP PRS across race/ethnicity background group in different datasets 28

Supplementary Figure 7: Distribution of UKBB+ICBP LDPRed2 DBP PRS across race/ethnicity background group in different datasets 29

Supplementary Figure 8: PVE comparison of SBP PRS summations when summation weights are trained on MGB Biobank with different approaches for scaling the component PRSs 30

Supplementary Figure 9: PVE comparison of DBP PRS summations when summation weights are trained on MGB Biobank with different approaches for scaling the component PRSs 31

Supplementary Figure 10: PVE comparison of SPB and DBP PRS summations when summation weights are trained on MGB Biobank with and without adjustment for history of antihypertensive medication use 32

Supplementary Figure 11: Distribution of highest performing single GWAS PRS and multi-PRS by race/ethnic background group in the TOPMed-BP dataset 33

Supplementary Figure 12: Distribution of highest performing single GWAS PRS and multi-PRS by groups defined by genetic ancestry in the TOPMed-BP dataset 34

Supplementary Figure 13: Visualization of ancestry-specific allele frequencies of SNPs from GWAS used to guide SBP PRS construction 35

Supplementary Figure 14: Visualization of ancestry-specific allele frequencies of SNPs from GWAS used to guide DBP PRS construction 36

Supplementary Figure 15: Association of SBP and DBP PRS with BP measures among All of Us individuals, stratified by groups defined by combination of self-reported race/ethnicity and genetic similarity 37

Supplementary Figure 16: Association of SBP and DBP PRS with BP measures among individuals who do not use antihypertensive medications, and stratified by race/ethnic background in the All of Us dataset 38

Supplementary Figure 17: Association of SBP and DBP PRS with BP measures among individuals who use antihypertensive medications, and stratified by race/ethnic background in the All of Us dataset 39

Supplementary Figure 18: Association of BP PRSs with BP measures by strata of age, obesity, and sex in White participants from All of Us 40

Supplementary Figure 19: Association of BP PRSs with BP measures stratified by age, obesity, and sex in Black participants from All of Us 41

Supplementary Figure 20: Association of BP PRSs with BP measures stratified by age, obesity, and sex in Hispanic/Latino participants from All of Us 42

Supplementary Figure 21: Association of BP PRSs with BP measures stratified by age, obesity, and sex in Asian participants from All of Us 43

Supplementary Figure 22: Association of SBP and DBP PRS with BP measures among individuals who do not use antihypertensive medications, by strata of age, obesity, and sex in All of Us ....................44
Supplementary Figure 23: Association of SBP and DBP PRS with BP measures among individuals who use antihypertensive medications, by strata of age, obesity, and sex in participants from All of Us ....................45
Supplementary Figure 24: Association of BP and hypertension PRS with prevalent clinical outcomes in the All of Us dataset ....................46
Supplementary Figure 25: Association of BP and hypertension PRS with prevalent clinical outcomes in the All of Us dataset among antihypertensive medication users ....................47
Supplementary Figure 26: Association of BP and hypertension PRS with prevalent clinical outcomes in the All of Us dataset among individuals not using antihypertensive medication 48
Supplementary Note 2: Parent study descriptions....................49
Amish....................49
ARIC ....................49
BioMe ....................50
CARDIA ....................51
CFS....................52
CHS ....................53
COPDGene ....................54
FHS....................55
GENOA....................56
GenSalt ....................57
HCHS/SOL ....................58
JHS ....................59
WHI....................60
MESA ....................61
THRV....................62
Supplementary Note 3: TOPMed and CCDG acknowledgements ....................64
Supplementary Note 4: TOPMed consortium members....................66
Supplementary References ....................72

# Supplementary Note 1: supplementary methods

## UK Biobank methods

**The UK Biobank dataset**

The UK Biobank (UKBB) cohort consists of 502,620 participants, recruited in the United Kingdom, and is described elsewhere in detail [1]. Participants answered questionnaires to assess medical conditions, lifestyle, and demographic information. Interviews were conducted by trained medical staff to assess medical history, health status and medication intake. At the time of the initial interview, participants had a medical exam which included the SBP and DBP and blood pressure measurements used in this study. The National Health Service National Research Ethics Service (ref. 11/NW/0382) gave approval for the study. As accepted elsewhere in genetic studies of BP phenotypes, SBP and DBP values were raised by 15 mmHg and 10 mmHg, respectively, in individuals using antihypertensive medications.

**Identifying individuals of self-identified Black identity, and of African Ancestry**

We were interested in assessing performance of BP PRS in individuals of Black identity in the UKBB cohort. We used self-reported race/ethnic background (UKBB Data-Field 21000) to select 8,646 individuals who self-reported as “Black or Black British” or “White and Black Caribbean” or “White and Black African” ethnicity, which were the study-defined ethnic identities that referenced “Black” or “African” identity.

We also identified a subset of individuals with predominately African genetic ancestry, defined as proportion of continental African ancestry$\geq$ 0.8. We used these individuals for a secondary analysis of PRS performance in ancestry-defined groups, and for a secondary analysis of scaling

+ matching of PRS distributions between datasets. To identify such individuals, we performed an unsupervised analysis of ancestry proportions: we followed Constantinescu et al. [2] in analyzing all UKB non-European individuals (i.e., we excluded from the UKB dataset all individuals self-reported being of European or White ethnicity). We cleaned and pruned the data by removing genotypes with ≥ 1% missingness, followed by LD pruning using PLINK with the settings --indep-pairwise 50 10 0.1, i.e., removing SNPs with LD>0.1 within 50Kb distances of each other, and with step size variant count =10. Next, we applied the SCOPE software [3] on an unrelated set of individuals to compute admixture proportions using unsupervised analysis with k=4 ancestries. The number of ancestries was selected based on the number of super populations in the results reported by Constantinescu et al. [2] We identified component corresponding to African ancestry as the one with high proportions in individuals with Black identity. Out of the Black individuals, 5,816 were selected as having predominately African ancestry.

**Genotype data and imputation**

UKBB participants were genotyped on two closely related genotyping arrays: In total, 488,282 participants were genotyped on two closely related arrays: UK BiLEVE: N = 49,939, and UK Biobank Axiom: 438,343, and genotypes were imputed using reference sequence data as previously described [4].

**PRS calculations**

The PRS models, consisting of selected genetic markers and associated weights, calculated in the primary analysis using PRSice 2 software [5] were ported to positions and alleles to match the

UKBB imputed genotypes (build hg19). Then, we applied PRSice without additional clumping (i.e. using the clumping from the TOPMed BP cohort) to the selected markers at the $5x10^{-8}$, $1x10^{-7}$, $1x10^{-5}$, and $1x10^{-2}$ p-value thresholds. In primary analysis, PRS were scaled to have mean 0 and variance 1 in the UKBB-Black dataset by subtracting the mean PRS value and dividing by its standard deviation, computed on the TOPMed-BP dataset. In secondary analyses we also performed scaling + matching of PRS across platforms as described latter. PRS based on summations, association analyses, and performance analysis of PRS were calculated as described in the main manuscript.

## Mass General Brigham Biobank methods

Samples, genomic data, and health information were obtained from the Mass General Brigham Biobank, a biorepository of consented patient samples at Mass General Brigham. Phenotypic data was extracted from the MGB Biobank on February 1, 2023.

### DNA samples

DNA samples are processed from whole blood that was collected as a dedicated research draw or as a clinical discard. Dedicated research samples are aimed to be processed within four hours of collection. Clinical discards are processed 24+ hours after collection. Whole blood is spun to buffy coat with a centrifuge and the buffy coat is stored in a freezer up to several months. The buffy coat is then extracted to DNA. The DNA is then placed in an ultralow freezer (-80ºC).Each DNA aliquot contains a minimum of 2 ug of DNA. The concentration varies.

### Genotyping

Samples have been genotyped using three versions of the biobank SNP array offered by Illumina that is designed to capture the diversity of genetic backgrounds across the globe. The first batch of data was generated on the Multi-Ethnic Genotyping Array (MEGA) array, the first release of this SNP array. The second, third, and fourth batches were generated on the Expanded Multi-Ethnic Genotyping Array (MEGA Ex) array. All remaining data were generated on the Multi-Ethnic Global (MEG) BeadChip.

**Imputation**

Prior to performing imputation, files were converted to VCF format, separated by chromosomes. When multiple probes measured the same genotypes, they were checked for concordance and were set to a missing value if the genotypes did not match. Files were uploaded to the Michigan Imputation Server, and Genotypes were imputed using TOPMed reference panel. Genomic coordinates are provided in GRCh38.

**Quality control**

We performed quality control using PLINK (v2.0. We filtered SNPs with low-quality imputation ($r < 0.5$), with missing call rates > 0.1, HWE p-value less than $1x10^{-6}$ and MAF <1%.

We computed principal component (PC) using PLINK: we pruned the genotype data using a window size of 1000 variants, sliding across the genome with a step size of 250 variants at a time, filtering out any SNPs with LD $R^2>0.1$. We used unrelated individuals (3rd degree, identified using PLINK) to compute the loadings for the first 10 PCs.

**PRS construction**

We constructed all PRS using PRSice 2, using the same SNPs as those selected in the various developed PRS, i.e. without further clumping. In primary analysis, we scaled the PRSs by subtracting the mean and dividing by SD computed on the TOPMed-BP dataset. In secondary analysis, we implemented additional scaling + matching of PRS across datasets as described later.

**Identification of individuals with predominant European ancestry**

To apply scaling + matching approaches of PRS across datasets (TOPMed, MGB Biobank, and UKBB), we identified individuals of predominately European ancestry, defined as having a proportion of at least 0.8 of continental European ancestry. We used ADMIXTURE software [6] to compute proportions of genetic ancestry in the MGB Biobank. First, we prepared the genetic data: we removed genotypes with ≥ 1% missingness, followed LD pruning using PLINK with the settings --indep-pairwise 50 10 0.1, i.e., removing SNPs with LD>0.1 within 50Kb distances of each other, and with step size variant count =10. Next, we applied ADMIXTURE on an unrelated set of individuals in an unsupervised analysis, with k=4. We identified the component corresponding to European ancestry as the component with higher proportions in individuals self-reported as White. Out of 33,855 individuals self-reported as White, 20,936 had predominately European ancestry.

**Ethics statement**

All Biobank subjects have provided their consent to join the Partners Biobank, which includes agreeing to provide a blood sample linked to the electronic medical record. Subjects also agree to be recontacted by the Partners Biobank staff as needed.

**Acknowledgements**

We thank Mass General Brigham Biobank for providing samples, genomic data, and health information data.

## Global genetic ancestry inference in TOPMed

Ancestry inference was performed by the TOPMed Informatics Research Center (IRC). First, local ancestry was inferred using RFMix [7], with default parameter settings except the following option: --node-size=5. Then, global ancestry was computed as for each participant as a weighted average of the ancestries in inferred local ancestry intervals. The reference panel used was the Human Genome Diversity Panel (HGDP) downloaded from the Stanford HGDP website http://hagsc.org/hgdp/files.html. Genomic coordinates were lifted over from genome build 37 to build 38. The 53 HGDP populations were merged into 7 super-populations: Sub-Saharan Africa, Central and South Asia, East Asia, Europe, Native America, Oceania, Middle East. Local ancestry inference was performed in two versions. First, for samples available in TOPMed freeze 6, RFMix V1 was used, and local ancestry was inferred for the autosomes only. Later, for samples participating only in freeze 8 (but not in freeze 6), and for the X-chromosome, local ancestry inference was performed using RFMix V2.

## Secondary analysis of scaling and scaling + matching to address PRS distribution differences across datasets

We considered a few approaches for scaling and scaling + matching PRS. Here scaling + matching refers to the idea of explicitly scaling PRSs in various datasets so that PRS distribution agree in some objective criterion across the datasets. Our primary scaling approach implicitly assumes that the distributions of PRSs match between datasets: we computed the means and SDs of PRSs in TOPMed-BP and used the same means and SDs to scale the corresponding PRSs in all datasets (TOPMed-BP, MGB Biobank, and UKBB). In secondary analysis, we also scaled PRS independently in each dataset using dataset-specific mean and SD. We attempted two scaling + matching approaches: matching PRS distributions across datasets in groups defined by (a) genetic ancestry, and (b) self-reported race/ethnicity. In each of these scaling + matching instances, we identified groups of individuals who are similar in either their genetic ancestry (a) or by self-reported race/ethnicity (b) and computed the means and SDs of PRS in these groups. Then, used these means and SDs to scale PRSs within the respective dataset. In mathematical notation, let $PRS_i^g$ be the value of a given PRS in individual $i$ from group $g$. In a given dataset, let the mean and SD of a PRS computed over all individuals from group $g$, be defined as:

$$\mu_g = \frac{1}{n_g}\sum_i PRS_i^g \,, \sigma_g = \sqrt{\frac{1}{n_g}\sum_i \left(PRS_i^g - \mu_g\right)^2}$$

We next scale all individuals within the dataset, regardless of their specific group, using $\mu_g$ and $\sigma_g$. Importantly, for individuals from group $g$, after applying the scaling transformation where $\widetilde{PRS}_i^g = \frac{(PRS_i^g - \mu_g)}{\sigma_g^2}$, we have that the new mean computed on this group is zero and the new SD is 1. Thus, applying the same idea on two different datasets means that similar groups of individuals in two different datasets (e.g. individuals of predominant European ancestry in

TOPMed-BP and individuals of predominant ancestry in MGB Biobank) have the same mean and SD of their PRSs. Thus, the PRS distributions match between well-defined sets of individuals. When scaling + matching between TOPMed-BP and MGB Biobank we used groups corresponding to (a) predominant (at least 80%) European ancestry and (b) White self-reported background. When scaling + matching between TOPMed-BP and UKBB we used groups corresponding to (a) predominant (at least 80%) African ancestry and (b) Black self-reported background.

## All of Us Methods

At the time of analysis, there were 98,590 WGS samples available from All of Us. Report of sequencing and quality control methods are provided in this link: https://www.researchallofus.org/wp-content/themes/research-hub-wordpress-theme/media/2022/06/All%20Of%20Us%20Q2%202022%20Release%20Genomic%20Quality%20Report.pdf . All of Us provided PCs and performed relatedness analysis. In this work we removed first- and second-degree relatives to generate a set of individuals in which the relatedness between any pair is of degree third and higher using the pre-computed pairs provided by All of Us. The data were accessed on September 13, 2022.

### Genetic ancestry in All of Us

The All of Us study team provided ancestry labels, however, the computation of ancestry in All of Us differed from that in TOPMed, UKBB, and MGB Biobank. Proportions of global ancestries were not computed, but rather an ancestry label was assigned to each participant according to their "location" in the PC space, guided by self-reported labels from the survey.

Accordingly, in All of Us one of the ancestries is “AMR” referring to Latino/Admixed American, whereas in TOPMed we refer instead to the parent ancestries of Admixed Hispanics/Latinos as European, African, and Amerindian. To clarify this difference we refer to “ancestry” groups in All of Us as groups defined by a combination of self-reported race/ethnicity and genetic similarity.

**BP phenotypes in All of Us**

We followed a previously develop hypertension analysis in All of Us using a Jupyter notebook that was made available to All of Us researchers via a workspace called “Demo – Hypertension Prevalence”. In brief, we extracted measured SBP and DBP physical exam data for all participants who have WGS data. Afterwards, the earliest measurements were selected for use. We used age at the age of BP measurement used, and exacted BMI and antihypertensive medications from either the electronic health record (EHR) or the physical exam data, and from the closest date to when BP was measured. Following the analysis in the shared Jupyter notebook, antihypertensive medications were defined as peripheral vasodilators, agents acting on the renin-angiotensin system, beta blocking agents, antihypertensives, calcium channel blockers, diuretics. SBP and DBP values were raised by 15 mmHg and 10 mmHg, respectively, in individuals with first date of using antihypertensive medications before or at the same year of their BP measurement. Association analyses were adjusted to age, sex at birth, BMI, and the first 10 PCs of genetic data.

**PRS construction**

We extract HapMap SNP in from the Hail table of the All of Us WGS data. We filtered out SNPs with call rate lower than 99%, with MAF<1% in the All of Us dataset, SNPs that failed QC filters, and SNPs with missing genotypes in ≥1% of the analytic sample. We then converted the resulting hail table into a PLINK file. Next, we constructed the PRS-CSx2 ancestry-specific PRS using PLINK v1.9, standardized them using TOPMed means and SDs, and applied PRS summation weights computed using the MGB dataset to combine the PRSs. We then standardized the combined PRS again using TOPMed means and SDs.

**Clinical outcomes**

We computed the association of PRS with multiple adverse clinical outcomes, including hypertension, and other outcomes known to be associated with hypertension: type 2 diabetes, chronic kidney disease, coronary artery disease, atrial fibrillation, and heart failure. Hypertension was defined according to the AHA guidelines, as SBP≥ 130, DBP≥ 80, or use of antihypertensive medications. For these, we used SBP, DBP, and medication information from the main analysis. For other clinical outcomes we used the All of Us data browser to identify the top medical condition in terms of patient number and its corresponding SNOMEDCode and OMOP Concept ID as mapped by the All of Us dataset (note that the same standard concepts were mapped to these SNOMEDCodes and OMOP Concept IDs). The codes and standard concept names are provided in Supplementary Table 9.

**Ethics statement**

The All of Us research program was approved by a single IRB, the "All of Us IRB", which is charged with reviewing the protocol, informed consent, and other participant-facing materials for the *All of Us* Research Program. The IRB follows the regulations and guidance of the Office for Human Research Protections for all studies, ensuring that the rights and welfare of research participants are overseen and protected uniformly. More information is provided online https://allofus.nih.gov/about/who-we-are/institutional-review-board-irb-of-all-of-us-research-program and in the All of Us design paper [8].

**Acknowledgements**

The All of Us Research Program is supported by the National Institutes of Health, Office of the Director: Regional Medical Centers: 1 OT2 OD026549; 1 OT2 OD026554; 1 OT2 OD026557; 1 OT2 OD026556; 1 OT2 OD026550; 1 OT2 OD 026552; 1 OT2 OD026553; 1 OT2 OD026548; 1 OT2 OD026551; 1 OT2 OD026555; IAA #: AOD 16037; Federally Qualified Health Centers: HHSN 263201600085U; Data and Research Center: 5 U2C OD023196; Biobank: 1 U24 OD023121; The Participant Center: U24 OD023176; Participant Technology Systems Center: 1 U24 OD023163; Communications and Engagement: 3 OT2 OD023205; 3 OT2 OD023206; and Community Partners: 1 OT2 OD025277; 3 OT2 OD025315; 1 OT2 OD025337; 1 OT2 OD025276. The All of Us Research Program would not be possible without the partnership of its participants.

# Supplementary Tables

## Supplementary Table 1: Characteristics of TOPMed-BP study participants.

| | Black | Asian | White | Hispanic/Latino |
|---|---|---|---|---|
| **N** | 14,746 | 4,671 | 30,943 | 12,131 |
| **Sex = Female (%)** | 9303 (63.1) | 2412 (51.6) | 20478 (66.2) | 7278 (60.0) |
| **Age (mean (SD))** | 53.94 (14.34) | 48.35 (12.97) | 57.85 (14.59) | 51.27 (14.57) |
| **Study (%)** | | | | |
| **Amish** | 0 ( 0.0) | 0 ( 0.0) | 1061 ( 3.4) | 0 ( 0.0) |
| **ARIC** | 1324 ( 9.0) | 0 ( 0.0) | 5991 (19.4) | 0 ( 0.0) |
| **BioMe** | 1964 (13.3) | 109 ( 2.3) | 1714 ( 5.5) | 3235 (26.7) |
| **CARDIA** | 1364 ( 9.2) | 0 ( 0.0) | 1660 ( 5.4) | 0 ( 0.0) |
| **CFS** | 319 ( 2.2) | 0 ( 0.0) | 235 ( 0.8) | 0 ( 0.0) |
| **CHS** | 688 ( 4.7) | 0 ( 0.0) | 2747 ( 8.9) | 31 ( 0.3) |
| **COPDGene** | 2217 (15.0) | 0 ( 0.0) | 3636 (11.8) | 0 ( 0.0) |
| **FHS** | 0 ( 0.0) | 0 ( 0.0) | 3093 (10.0) | 11 ( 0.1) |
| **GENOA** | 1074 ( 7.3) | 0 ( 0.0) | 0 ( 0.0) | 0 ( 0.0) |
| **GenSalt** | 0 ( 0.0) | 1811 (38.8) | 0 ( 0.0) | 0 ( 0.0) |
| **HCHS_SOL** | 0 ( 0.0) | 0 ( 0.0) | 0 ( 0.0) | 7532 (62.1) |
| **JHS** | 3273 (22.2) | 0 ( 0.0) | 0 ( 0.0) | 0 ( 0.0) |
| **MESA** | 1098 ( 7.4) | 599 (12.8) | 1855 ( 6.0) | 1020 ( 8.4) |
| **THRV** | 0 ( 0.0) | 1955 (41.9) | 0 ( 0.0) | 0 ( 0.0) |
| **WHI** | 1425 ( 9.7) | 197 ( 4.2) | 8951 (28.9) | 302 ( 2.5) |
| **Current smoke =Yes (%)** | 4566 (31.0) | 1035 (22.2) | 6688 (21.6) | 2487 (20.5) |
| **BMI (mean (SD))** | 30.20 (7.09) | 24.14 (3.46) | 27.34 (5.60) | 29.96 (6.31) |
| **SBP (mean (SD))** | 136.03 (23.73) | 126.35 (23.15) | 128.93 (21.70) | 130.50 (23.99) |
| **DBP (mean (SD))** | 80.81 (12.76) | 76.89 (13.44) | 75.73 (11.60) | 76.68 (12.83) |
| **Antihypertensive med = Yes(%)** | 5950 (47.5) | 1021 (21.9) | 7640 (28.0) | 3652 (30.1) |
| **Hypertension= Yes(%)** | 10289 (73.1) | 2407 (51.5) | 17393 (59.2) | 6875 (56.7) |

## Supplementary Table 2: Characteristics of Black participants from UK Biobank.

| | Female | Male |
|---|---|---|
| **N** | 5015 | 3628 |
| **Age (mean (SD))** | 51.88 (7.92) | 51.67 (8.20) |
| **BMI (mean (SD))** | 30.03 (5.98) | 28.32 (4.29) |
| **SBP (mean (SD))** | 142.33 (23.29) | 144.97 (21.35) |
| **DBP (mean (SD))** | 87.24 (13.13) | 87.30 (12.80) |
| **Antihypertensive med = Yes (%)** | 1530(30.5) | 985(27.1) |
| **Hypertension = Yes (%)** | 3800 (77.0) | 2905 (81.5) |
| **Current Smoke =Yes (%)** | 390 (7.8) | 392 (10.8) |

## Supplementary Table 3: Characteristics of MGB Biobank study participants.

| | Black | Asian | White | Hispanic/Latino | Other | Unknown |
|---|---|---|---|---|---|---|
| **N** | 1837 | 736 | 30935 | 1341 | 917 | 668 |
| **Age (mean (SD))** | 55.25 (16.08) | 50.99 (15.92) | 62.15 (16.37) | 52.21 (16.49) | 49.41 (16.09) | 60.79 (16.57) |
| **Gender = Female (%)** | 1162 (63.30) | 440 (59.80) | 16646 (53.8) | 921 (68.7) | 585 (63.8) | 357 (53.4) |
| **SBP (mean (SD))** | 127.30 (12.60) | 119.54 (12.54) | 125.73 (12.29) | 121.43 (12.45) | 121.81 (11.62) | 125.36 (12.41) |
| **DBP (mean (SD))** | 76.63 (7.96) | 73.81 (8.37) | 74.99 (7.44) | 72.87 (7.30) | 74.06 (7.68) | 75.04 (7.95) |
| **Obesity= Yes(%)** | 1135 (61.80) | 130 (17.70) | 12629 (40.80) | 745 (55.60) | 471 (51.40) | 253 (37.90) |
| **Antihypertensive med= Yes (%)** | 1423 (77.50) | 411 (55.80) | 22513 (72.80) | 809 (60.30) | 604 (65.90) | 440 (65.90) |
| **Hypertension= Yes (%)** | 859 (46.80) | 139 (18.90) | 10314 (33.30) | 428 (31.90) | 238 (26.00) | 210 (31.40) |

MGB participant characteristics are based on a database query from February 1, 2023. Age is at the time of database query. SBP and DBP values were the medians in the health records. Obesity status was determined based on chart-validated algorithm [9] using BMI values, obesity and diabetes diagnosis codes, with threshold set by having positive predictive value of 0.90. Hypertension was determined based on a chart-validated algorithm using prescription and diagnosis codes related to hypertension, with threshold set by having positive predictive value of 0.95. Hypertension medication refers to a history of having any hypertension medication as described in the main manuscript.

## Supplementary Table 4: Characteristics of All of Us participants

| | Black | Asian | White | Hispanic/Latino |
|---|---|---|---|---|
| **N** | 19,441 | 2,891 | 48,155 | 18,034 |
| **Sex at birth= Female (%)** | 10754 (55.30) | 1725 (59.70) | 28705 (59.60) | 12277 (68.10) |
| **Age (mean (SD))** | 47.36 (14.80) | 42.96 (16.67) | 53.65 (16.61) | 44.27 (15.79) |
| **BMI (mean (SD))** | 30.70 (8.30) | 24.99 (4.83) | 28.78 (6.93) | 30.24 (6.91) |
| **SBP (mean (SD))** | 134.10 (21.94) | 122.74 (18.13) | 130.38 (19.39) | 127.36 (19.30) |
| **DBP (mean (SD))** | 82.86 (13.86) | 76.82 (11.87) | 79.67 (12.01) | 78.76 (12.32) |
| **Antihypertensive med = Yes (%)** | 4263 (22.10) | 462 (16.10) | 14528 (30.30) | 4163 (23.20) |
| **Hypertension= Yes (%)** | 13421 (69.50) | 1403 (49.00) | 31181 (65.10) | 10469 (58.40) |
| **Atrial fibrillation= Yes (%)** | 392 (2.00) | 39 (1.30) | 2952 (6.10) | 446 (2.50) |
| **Coronary artery disease =Yes (%)** | 386 (2.00) | 48 (1.70) | 2080 (4.30) | 366 (2.00) |
| **Chronic kidney disease= Yes (%)** | 773 (4.00) | 50 (1.70) | 1966 (4.10) | 636 (3.50) |
| **Heart failure= Yes (%)** | 640 (3.30) | 34 (1.20) | 1511 (3.10) | 377 (2.10) |
| **Type II diabetes= Yes (%)** | 1200 (6.20) | 94 (3.30) | 2459 (5.10) | 1080 (6.00) |

### Supplementary Table 5: Number of TOPMed + UKB individuals participating in each of the PRS analyses.

| | BBJ | MVP | UKB + ICBP & PRS-CSx1&2 | Multi PRS approaches other than PRS-CSx1&2 |
|---|---|---|---|---|
| **Black** | 23107 | 23107 | 23107 | 10578 |
| **Asian** | 4671 | 4671 | 4671 | 4671 |
| **White** | 30952 | 30952 | 15548 | 15548 |
| **Hispanic/Latino** | 12131 | 12131 | 12131 | 12131 |
| **Multi-ethnic** | 62501 | 62501 | 47097 | 34562 |

The number of individuals refer to the primary evaluation analysis in the manuscript. Multi PRS approaches refer to unweighted and weighted summation of PRSs, including PRS-CSx.

## Supplementary Table 6: PRS-CSx2 means and SDs in the TOPMed-BP dataset

| Trait | PRS Type | Mean | SD |
|---|---|---|---|
| **SBP** | PRS-CSx2 EAS | 4.20E-07 | 2.43E-07 |
| **SBP** | PRS-CSx2 AFR | 9.29E-07 | 2.38E-06 |
| **SBP** | PRS-CSx2 EUR | 1.49E-06 | 2.55E-07 |
| **DBP** | PRS-CSx2 EAS | 2.04E-07 | 2.46E-07 |
| **DBP** | PRS-CSx2 AFR | -1.49E-06 | 1.94E-06 |
| **DBP** | PRS-CSx2 EUR | 7.17E-07 | 2.19E-07 |

For each ancestry-specific PRS, the table provides its mean and SD in the multi-ethnic TOPMed-BP dataset. All PRS were standardized using TOPMed-BP means and SDs.

## Supplementary Table 7 : Means and SDs of the weighted PRS-CSx2 sum in the TOPMed-BP dataset

| Trait | Race/ethnicity | Mean | SD |
|---|---|---|---|
| SBP | All | -2.32E-14 | 2.62 |
| SBP | Black | -1.62E-14 | 1.84 |
| SBP | Asian | -1.91E-14 | 2.64 |
| SBP | Hispanic/Latino | -2.35E-14 | 2.63 |
| SBP | White | -2.36E-14 | 2.67 |
| DBP | All | 4.60E-15 | 1.57 |
| DBP | Black | 5.39E-15 | 1.56 |
| DBP | Asian | 5.41E-15 | 1.90 |
| DBP | Hispanic/Latino | 5.07E-15 | 1.70 |
| DBP | White | 4.45E-15 | 1.57 |

The means and SDs are provided for the combined PRS: after combining standardized ancestry-specific PRS (standardized based on means and SDs in Supplementary Table 6, and weighted using weights in Supplementary Table 8), primary analysis used background-specific weights. Therefore, PRS were standardized in each background group separately.

## Supplementary Table 8 : MGB Biobank-trained PRS summation weights for PRS-CSx2

| Trait | PRS Type | All | Asian | Black | Hispanic/Latino | White |
|---|---|---|---|---|---|---|
| SBP | PRS-CSx2 AFR | 1.81 | 0.48 | 2.00 | 1.78 | 1.82 |
| SBP | PRS-CSx2 EAS | 0.68 | 1.17 | 0.35 | 0.50 | 0.69 |
| SBP | PRS-CSx2 EUR | 3.28 | 2.58 | 2.31 | 3.37 | 3.35 |
| DBP | PRS-CSx2 AFR | 0.69 | 0.63 | 1.42 | 0.76 | 0.61 |
| DBP | PRS-CSx2 EAS | 0.40 | 0.83 | 0.38 | 0.53 | 0.39 |
| DBP | PRS-CSx2 EUR | 1.71 | 1.93 | 1.44 | 1.82 | 1.71 |

## Supplementary Table 9: Standard concept names used to define clinical outcomes in All of Us

| **Outcom** | **SNOMEDCode,<br>OMOP concept ID** | **Standard concept names** |
|---|---|---|
| **Type 2 diabetes** | SNOMEDCode: 44054006<br>OMOP concept ID: 201826 | Type 2 diabetes mellitus |
| **Atrial fibrillation** | SNOMEDCode: 49436004<br>OMOP concept ID: 313217 | Atrial fibrillation |
| **Chronic kidney disease** | SNOMEDCode: 709044004<br>OMOP concept ID: 46271022 | Chronic kidney disease |
| **Heart Failure** | SNOMEDCode: 84114007<br>OMOP concept ID:316139 | Heart failure |
| **Coronary artery disease** | SNOMEDCode: 53741008<br>OMOP concept ID: 317576 | Coronary arteriosclerosis |

For each outcome, standard concept names were pre-grouped by the All of Us database via the corresponding SNOMEDcode and OMOP concept ID provided.

## Supplementary Figures

## Supplementary Figure 1: Estimated phenotypic and residual variances of SBP by study and diversity background.

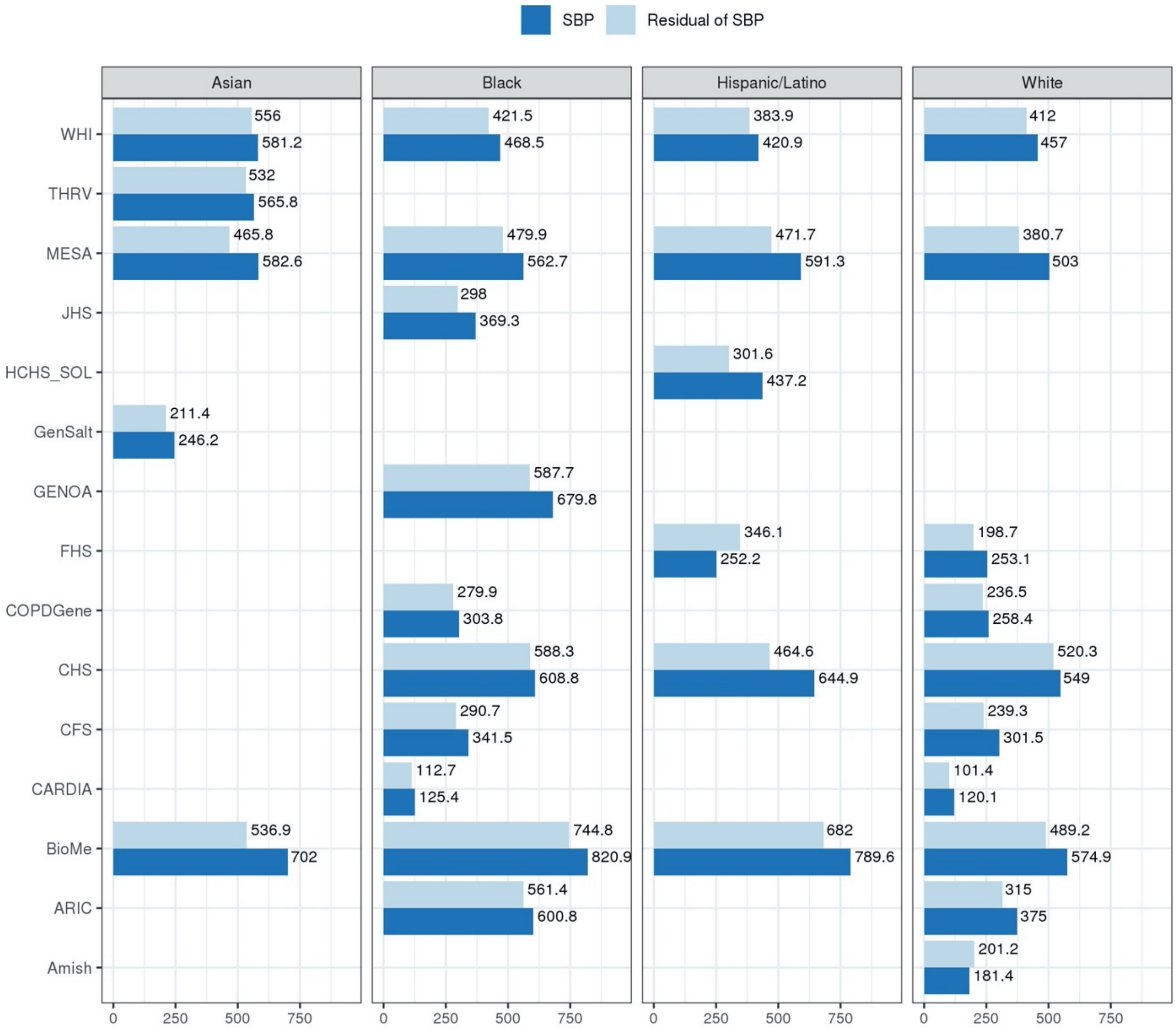


Phenotypic variances were estimated based on the raw phenotypes. Residual variances were estimated after regressing SBP on covariates (age, age$^2$, BMI, sex, smoking status, and 11 PCs). We used unrelated individuals for these computations. Abbreviations and definitions. BMI: body mass index; PC: principal component; SBP: systolic blood pressure.

## Supplementary Figure 2: Estimated phenotypic and residual variances of DBP by study and diversity background.

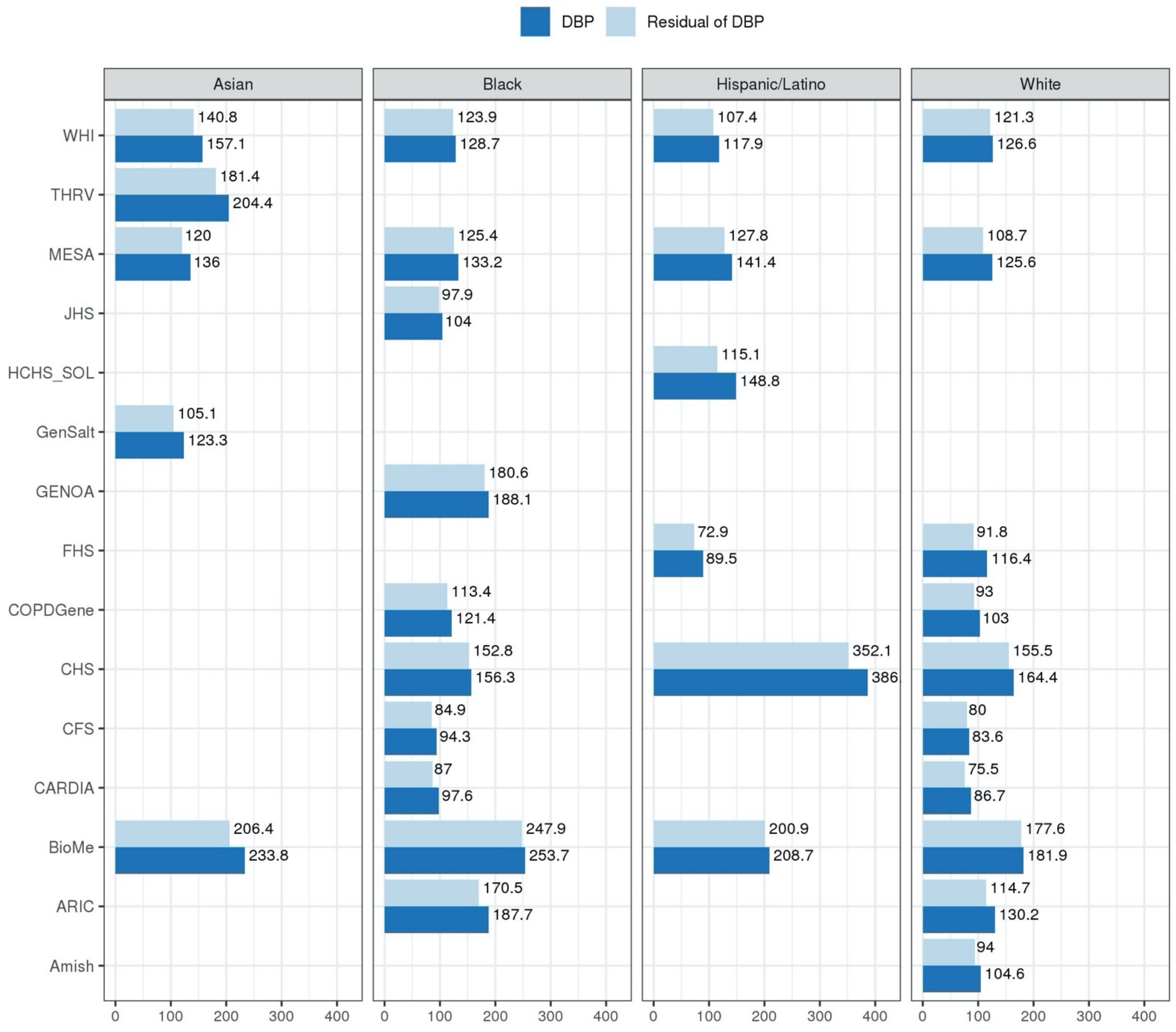


Phenotypic variances were estimated based on the raw phenotypes. Residual variance were estimated after regressing DBP on covariates (age, age$^2$, BMI, sex, smoking status, study site and 11 PCs). We used unrelated individuals for these computations. Abbreviations and definitions. BMI: body mass index; DBP: diastolic blood pressure; PC: principal component.

## Supplementary Figure 3: Estimated global proportions of continental ancestries in TOPMed-BP participants.

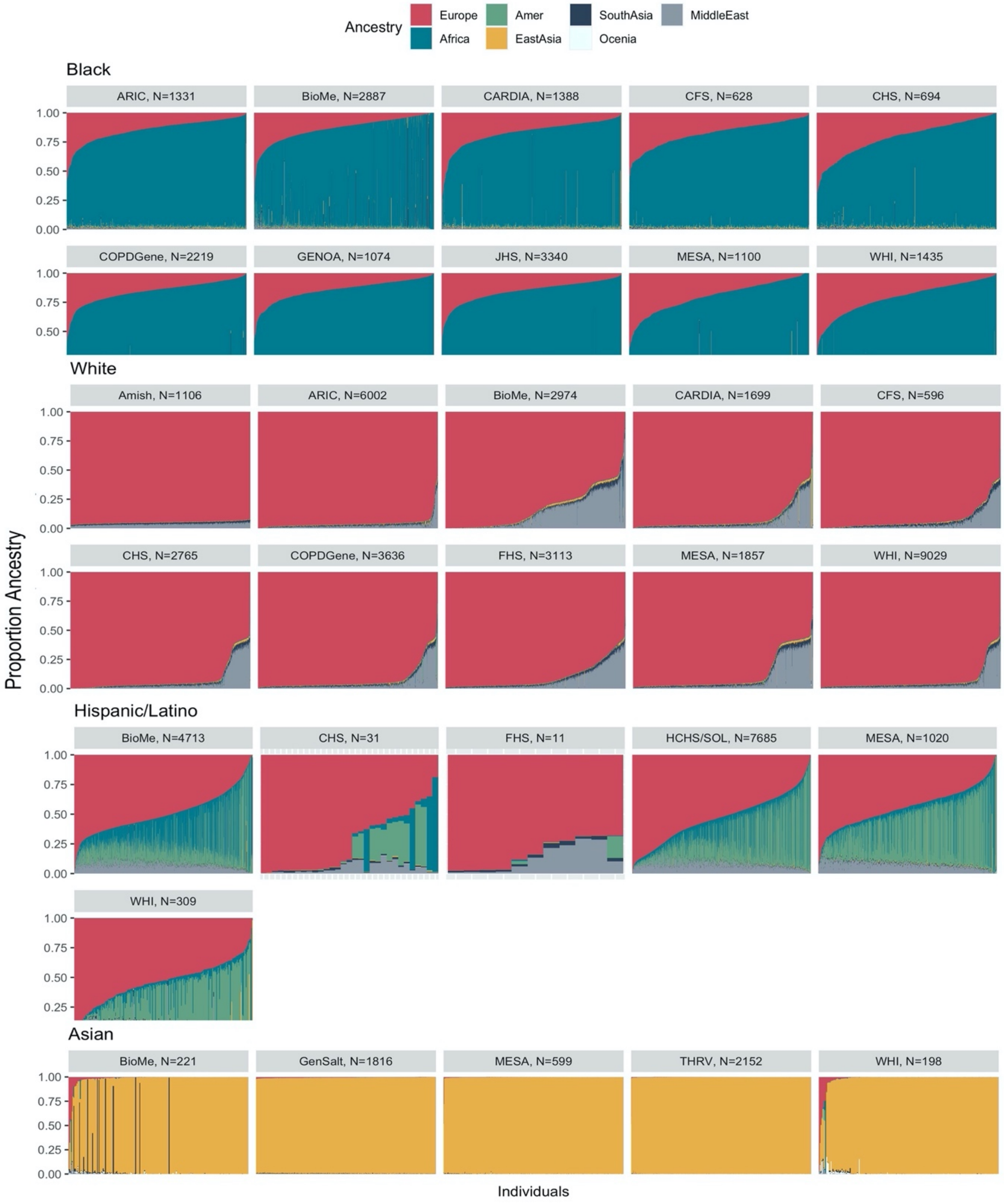


Each vertical line corresponds to a person from the corresponding background and TOPMed study. The colors of the lines represent the estimated proportions of global ancestries, in each individual, after sorting by estimated proportion of European continental ancestry.

Supplementary Figure 4: Comparison of variance explained according to weighting method: Background-specific versus Multi-ethnic PRS combination weights derived from MGB Biobank.

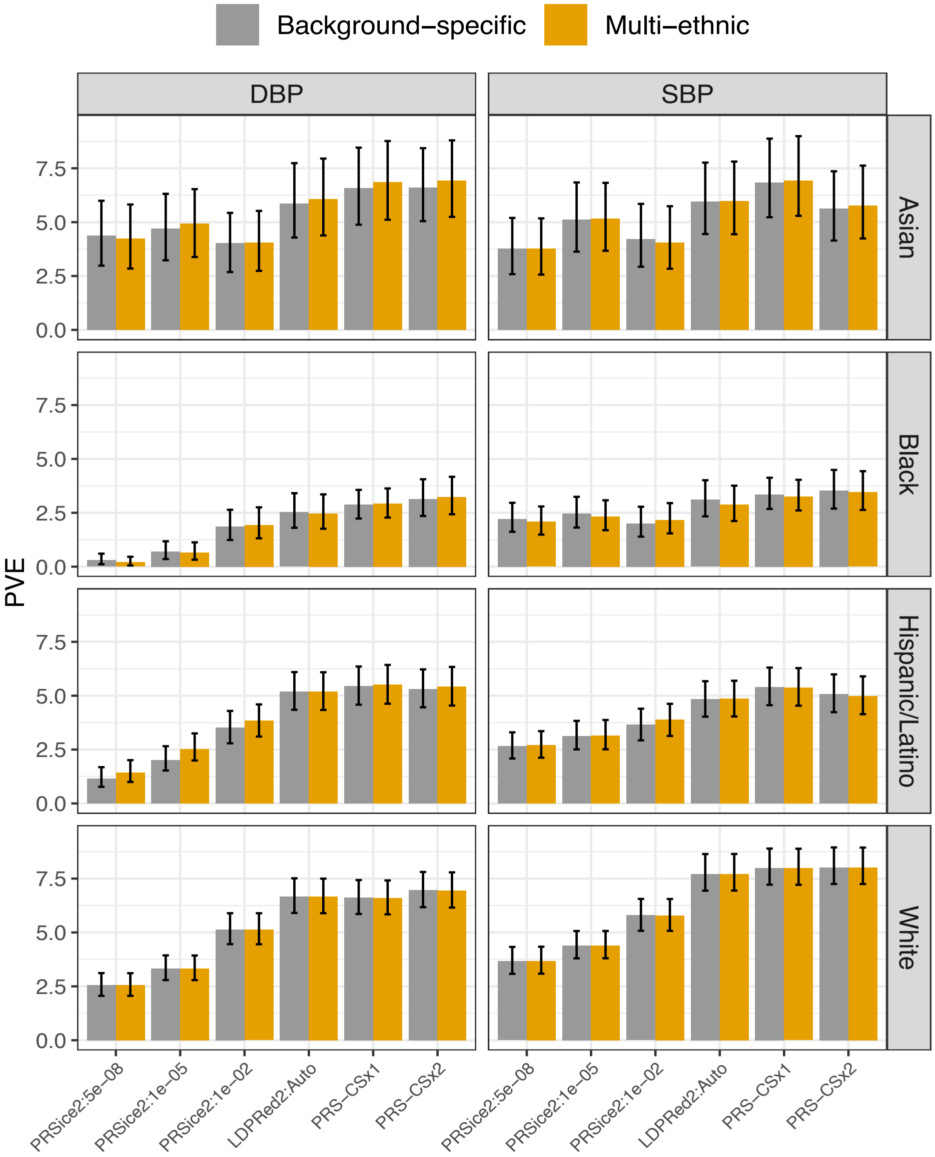


Estimated variance explained by PRS based on weighted combinations in the TOPMed-BP datasets (and UKBB Black individuals), stratified by diversity background. Combination weights were computed using the MGB Biobank dataset either using the multi-ethnic or background-specific dataset. The height of each bar represents the estimated PVE and intervals represent the 95% confidence intervals based on the 2.5% and 97.5% distribution percentiles from bootstrap performed using unrelated individuals. The left column corresponds to SBP PRSs and the right column to DBP PRSs. The sample sizes used in each of the analyses represented by the different bars in the figure are provided in Supplementary Table 5 (columns corresponding to multi-PRS approaches). Abbreviations and definitions. BP: blood pressure; LDPred: Bayesian PRS method (LDPred2 denotes a specific software implementation of the LDPred algorithm); PRS-CSx: continuous shrinkage cross-population PRS method; PRS: polygenic risk score; PRSice2: software for computing PRS based on the clumping & thresholding methodology; PVE: percent variance explained; SBP: systolic blood pressure; TOPMed: Trans-Omics for Precision Medicine; UKBB: UK biobank.

## Supplementary Figure 5: Comparison of variance explained according to LD reference panel choice: GWAS-matched, target-matched, or TOPMed LD-panels.

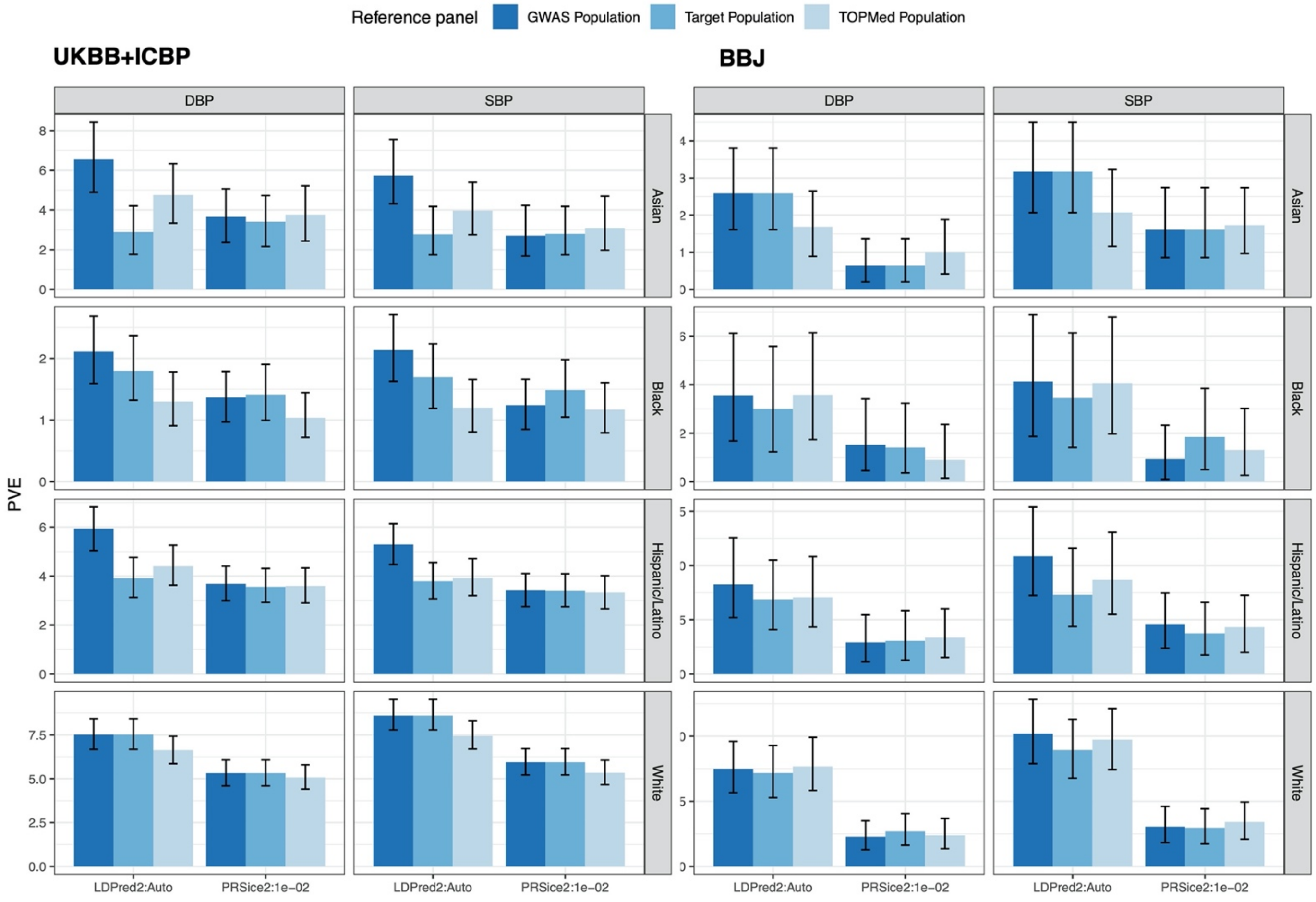


Estimated variance explained by PRS based on single GWAS (UKBB+ICBP and BBJ) in the TOPMed-BP datasets, stratified by diversity background. LD reference panel was either the complete TOPMed-BP dataset, or a subset of the TOPMed-BP dataset with population matching the discovery GWAS in ancestry make-up. The height of each bar represents the estimated PVE and intervals represent the 95% confidence intervals based on the 2.5% and 97.5% distribution percentiles from bootstrap performed using unrelated individuals. The left column corresponds to SBP PRSs and the right column to DBP PRSs. The sample sizes used in each of the analyses represented by the different bars in the figure are provided in Supplementary Table 5 (columns corresponding to UKBB+ICBP, and BBJ GWASs). Abbreviations and definitions. BBJ: Biobank Japan; GWAS: genome-wide association study; LD: linkage disequilibrium; LDPred: Bayesian PRS method (LDPred2 denotes a specific software implementation of the LDPred algorithm); PRS: polygenic risk score; PRSice2: software for computing PRS based on the clumping & thresholding methodology; SBP: systolic blood pressure; TOPMed: Trans-Omics for Precision Medicine; UKBB+ICBP: United Kingdom biobank and the International Consortium of Blood Pressure.

## Supplementary Figure 6: Distribution of UKBB+ICBP LDPRed2 SBP PRS across race/ethnicity background group in different datasets

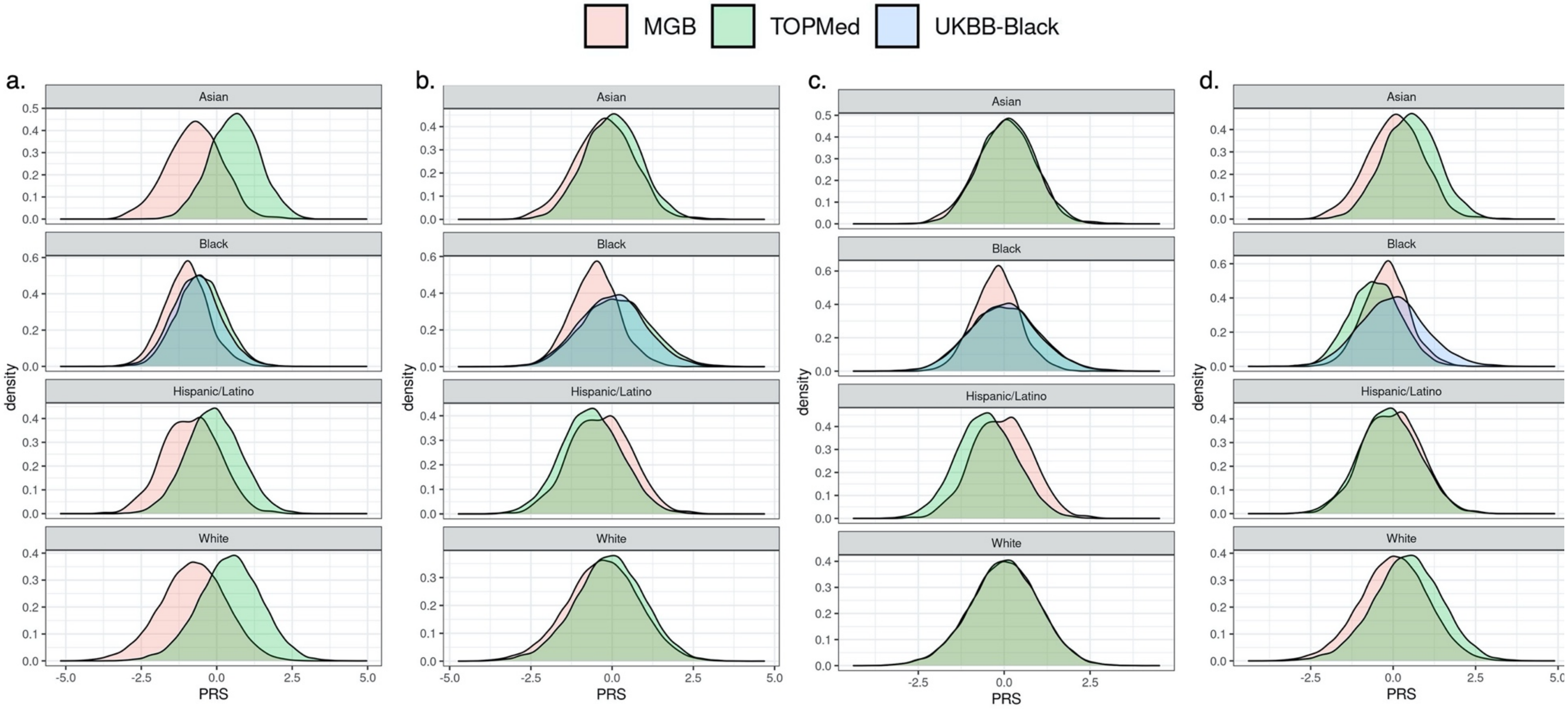


Comparison of SBP PRS distributions in MGB Biobank, TOPMed, and UKBB Black individuals when using different approaches for PRS scaling and scaling + matching. (a) TOPMed scaling (PRS are scaled using mean and standard deviations estimated on the TOPMed-BP dataset); (b) scaling + matching using groups defined by genetic ancestry (European ancestry when matching MGB Biobank to TOPMed, and African ancestry when matching UKBB Black individuals to TOPMed); (c) scaling + matching using groups defined by self-reported race/ethnicity (White or Black for MGB Biobank and UKBB, respectively); (d) dataset-specific scaling (PRS in each dataset are independently scaled to have mean 0 and variance 1 in the dataset). Abbreviations and definitions. LD: linkage disequilibrium; LDPred: Bayesian PRS method (LDPred2 denotes a specific software implementation of the LDPred algorithm); MGB: Mass General Brigham; PRS: polygenic risk score; SBP: systolic blood pressure; TOPMed: Trans-Omics for Precision Medicine; UKBB+ICBP: United Kingdom biobank and the International Consortium of Blood Pressure.

## Supplementary Figure 7: Distribution of UKBB+ICBP LDPRed2 DBP PRS across race/ethnicity background group in different datasets

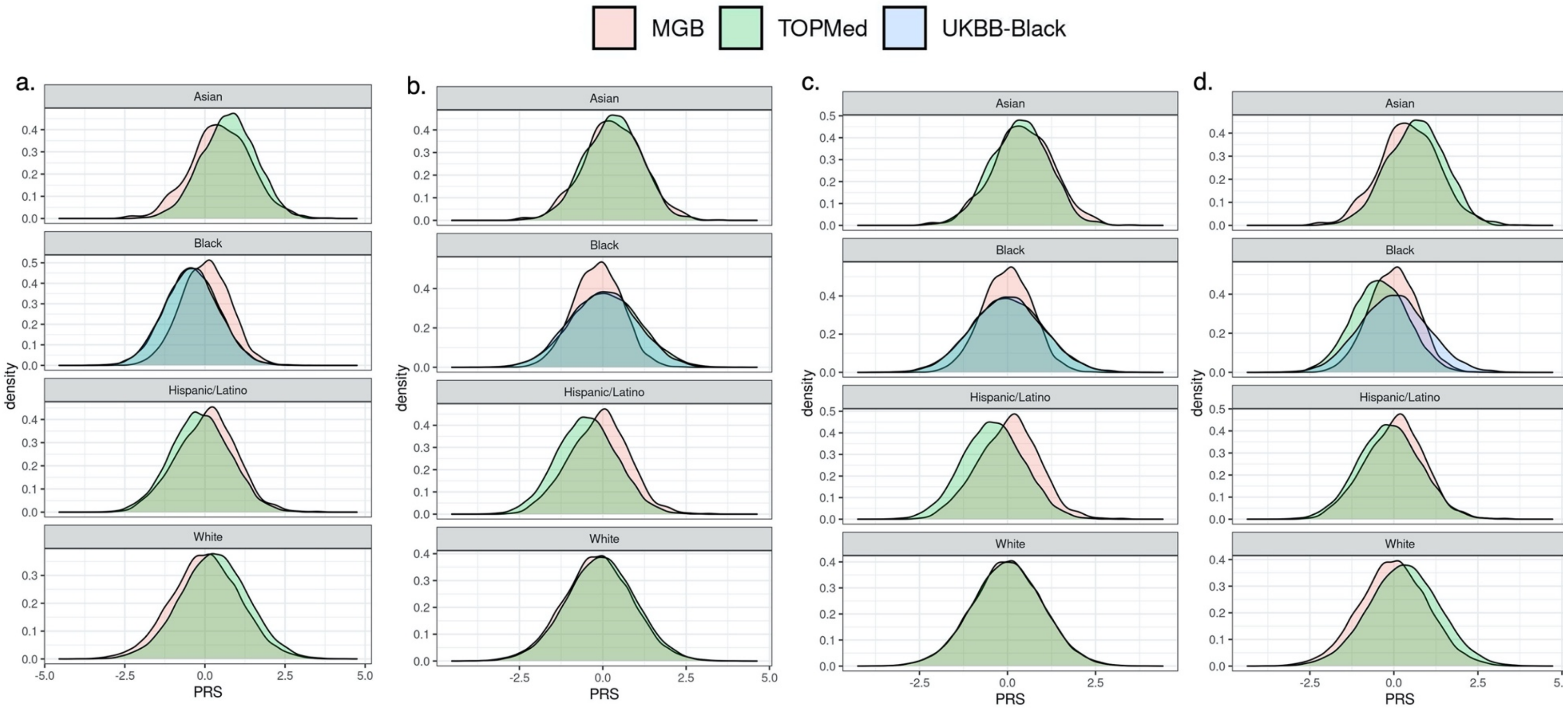


Comparison of DBP PRS distributions in MGB Biobank, TOPMed, and UKBB Black individuals when using different approaches for PRS scaling and scaling + matching. (a) TOPMed scaling (PRS are scaled using mean and standard deviations estimated on the TOPMed-BP dataset); (b) scaling + matching using groups defined by genetic ancestry (European ancestry when matching MGB Biobank to TOPMed, and African ancestry when matching UKBB Black individuals to TOPMed); (c) scaling + matching using groups defined by self-reported race/ethnicity (White or Black for MGB Biobank and UKBB, respectively); (d) dataset-specific scaling (PRS in each dataset are independently scaled to have mean 0 and variance 1 in the dataset). Abbreviations and definitions. DBP: diastolic blood pressure; LD: linkage disequilibrium; LDPred: Bayesian PRS method (LDPred2 denotes a specific software implementation of the LDPred algorithm); MGB: Mass General Brigham; PRS: polygenic risk score; TOPMed: Trans-Omics for Precision Medicine; UKBB+ICBP: United Kingdom biobank and the International Consortium of Blood Pressure.

## Supplementary Figure 8: PVE comparison of SBP PRS summations when summation weights are trained on MGB Biobank with different approaches for scaling the component PRSs

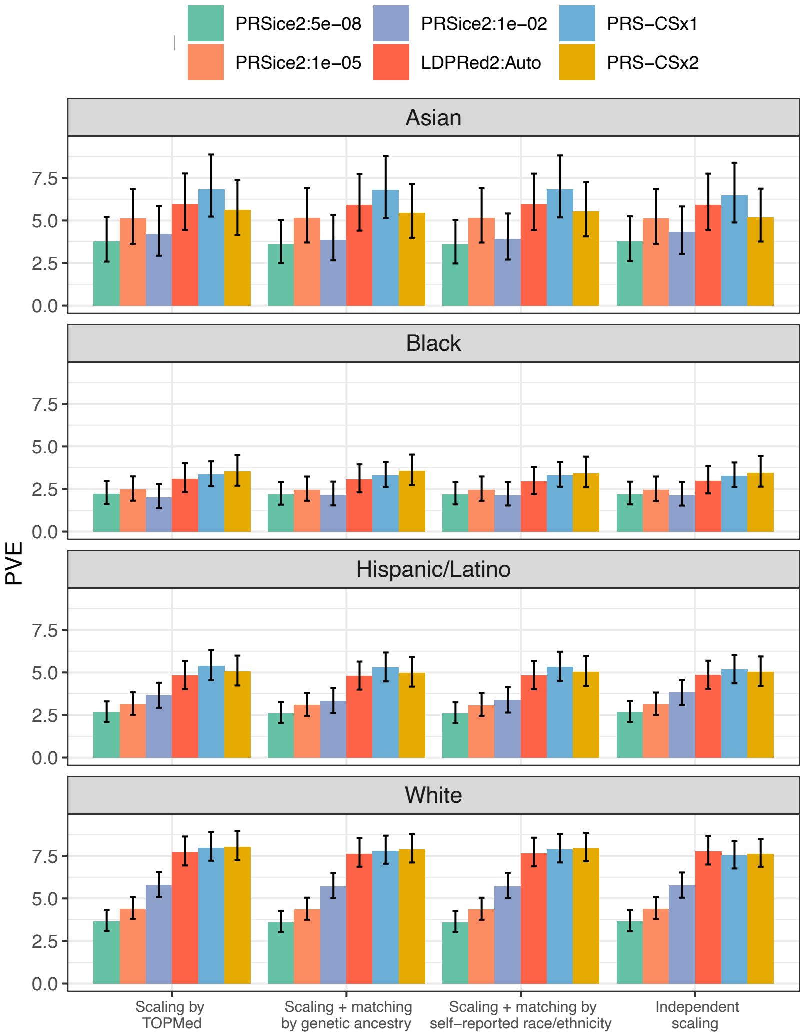


The height of each bar represents estimated PVE, and intervals represent the 95% confidence intervals based on the 2.5% and 97.5% distribution percentiles from bootstrap performed using unrelated individuals. SBP PRS PVEs were computed in TOPMed-BP and UKBB Black individuals, where PRS summation weights were trained using biobank data. Different PRS scaling approaches across datasets were taken. TOPMed scaling (PRS are scaled using mean and standard deviations estimated on the TOPMed-BP dataset); scaling + matching using groups defined by genetic ancestry (European ancestry when matching MGB Biobank to TOPMed, and African ancestry when matching UKBB Black individuals to TOPMed); scaling + matching using groups defined by self-reported race/ethnicity (White or Black for MGB Biobank and UKBB, respectively); dataset-specific, independent, scaling (PRS in each dataset are independently scaled to have mean 0 and variance 1 in the dataset). The sample sizes used in each of the analyses represented by the different bars in the figure are provided in Supplementary Table 5. Abbreviations and definitions. GWAS: genome-wide association study; LDPred: Bayesian PRS method (LDPred2 denotes a specific software implementation of the LDPred algorithm); MGB: Mass General Brigham; PRS-CSx: continuous shrinkage cross-population PRS method; PRS: polygenic risk score; PRSice2: software for computing PRS based on the clumping & thresholding methodology; SBP: systolic blood pressure; TOPMed: Trans-Omics for Precision Medicine.

## Supplementary Figure 9: PVE comparison of DBP PRS summations when summation weights are trained on MGB Biobank with different approaches for scaling the component PRSs

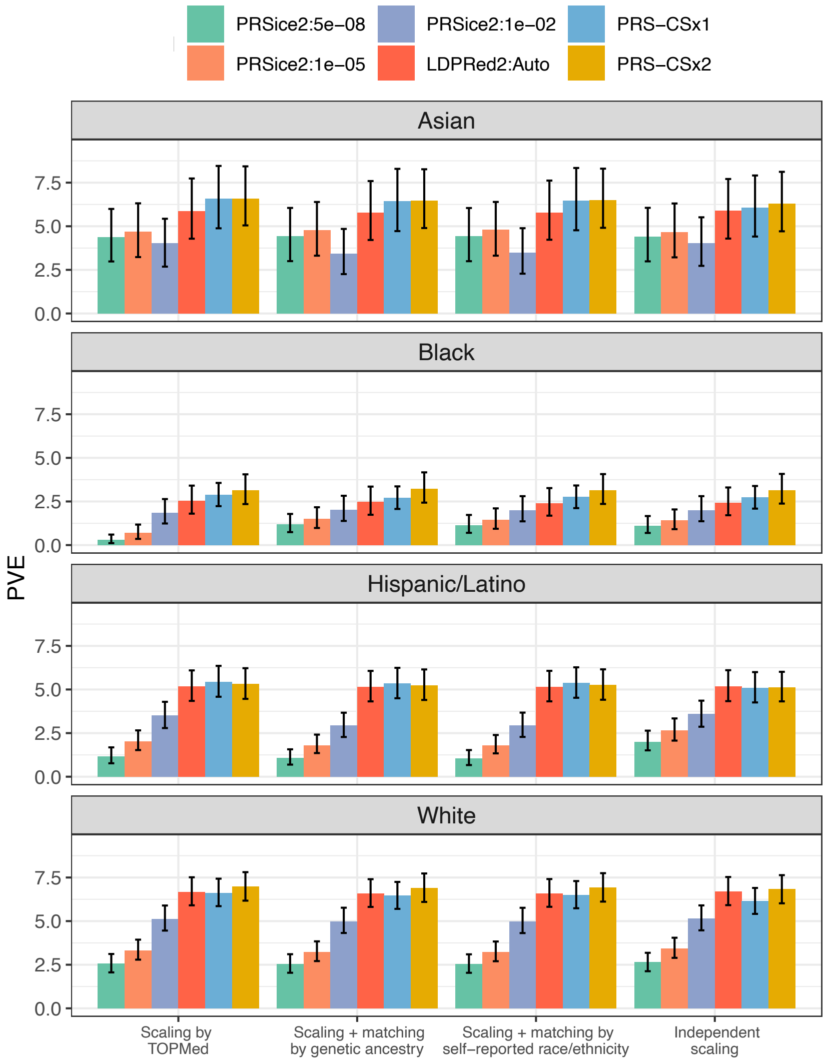


The height of each bar represents estimated PVE, and intervals represent the 95% confidence intervals based on the 2.5% and 97.5% distribution percentiles from bootstrap performed using unrelated individuals. DBP PRS PVEs were computed in TOPMed-BP and UKBB Black individuals, where PRS summation weights were trained using MGB Biobank data. Different PRS scaling approaches across datasets were taken. TOPMed scaling (PRS are scaled using mean and standard deviations estimated on the TOPMed-BP dataset); scaling + matching using groups defined by genetic ancestry (European ancestry when matching MGB Biobank to TOPMed, and African ancestry when matching UKBB Black individuals to TOPMed); scaling + matching using groups defined by self-reported race/ethnicity (White or Black for MGB Biobank and UKBB, respectively); dataset-specific, independent, scaling (PRS in each dataset are independently scaled to have mean 0 and variance 1 in the dataset). The sample sizes used in each of the analyses represented by the different bars in the figure are provided in Supplementary Table 5. Abbreviations and definitions. DBP: diastolic blood pressure; GWAS: genome-wide association study; LDPred: Bayesian PRS method (LDPred2 denotes a specific software implementation of the LDPred algorithm); MGB: Mass General Brigham; PRS-CSx: continuous shrinkage cross-population PRS method; PRS: polygenic risk score; PRSice2: software for computing PRS based on the clumping & thresholding methodology; TOPMed: Trans-Omics for Precision Medicine.

## Supplementary Figure 10: PVE comparison of SPB and DBP PRS summations when summation weights are trained on MGB Biobank with and without adjustment for history of antihypertensive medication use

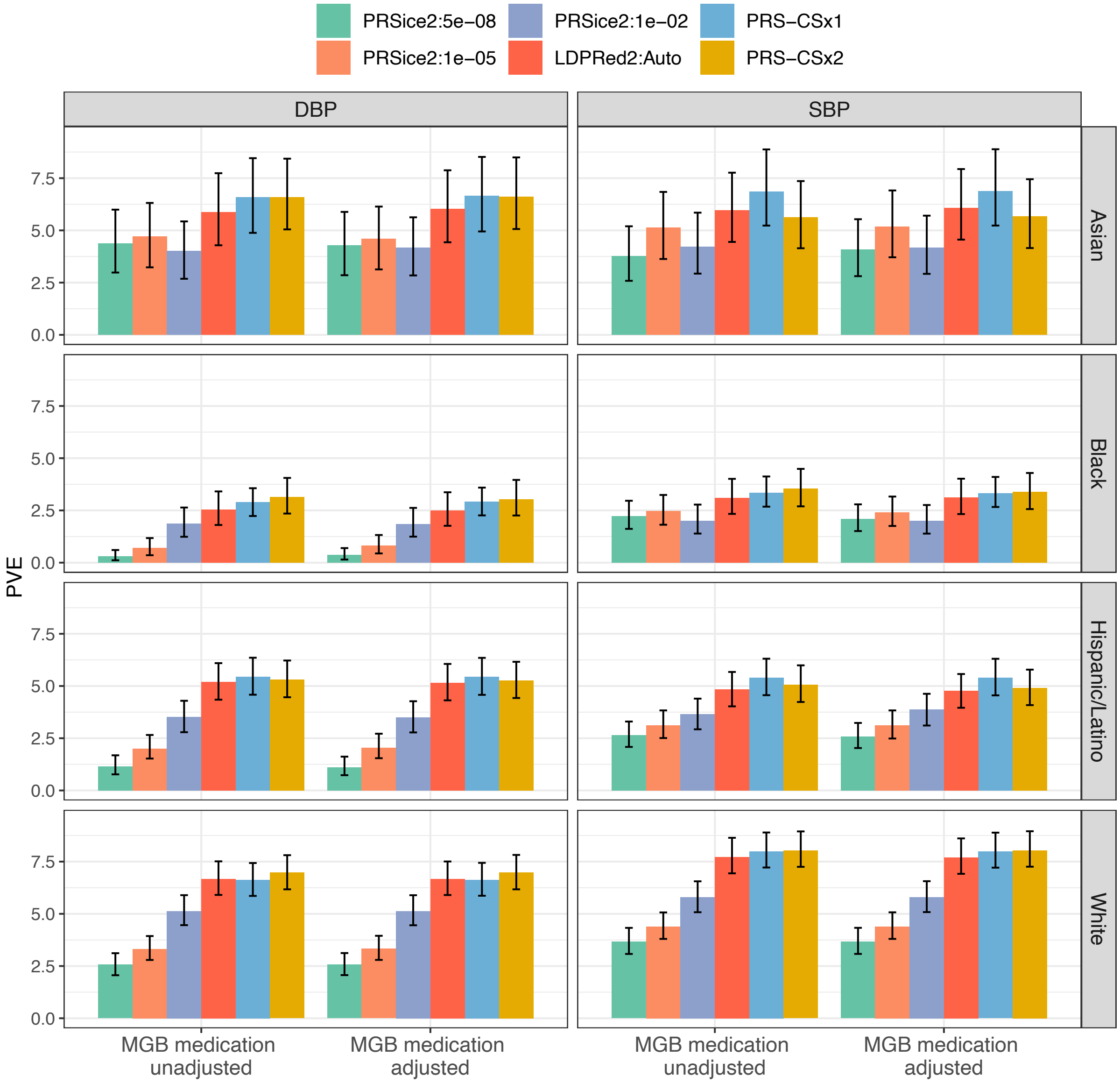


The height of each bar represents estimated PVE, and intervals represent the 95% confidence intervals based on the 2.5% and 97.5% distribution percentiles from bootstrap performed using unrelated individuals. SBP and DBP PRS PVEs were computed in TOPMed-BP and UKBB Black individuals, where PRS summation weights were trained using MGB Biobank data. "MGB medication adjusted" refers to values of SBP and DBP in MGB Biobank being raised by 15 and 10 mmHg in individuals with history of antihypertensive medication use. "MGB medication unadjusted" refers to an analysis that did not perform such adjustment of BP values. PRS in MGB Biobank were scaled as in the primary analysis, using means and SDs from the TOPMed-BP dataset. The sample sizes used in each of the analyses represented by the different bars in the figure are provided in Supplementary Table 5 (columns corresponding to multi-PRS approaches). Abbreviations and definitions. DBP: diastolic blood pressure; GWAS: genome-wide association study; LDPred: Bayesian PRS method (LDPred2 denotes a specific software implementation of the LDPred algorithm); MGB: Mass General Brigham; PRS-CSx: continuous shrinkage cross-population PRS method; PRS: polygenic risk score; PRSice2: software for computing PRS based on the clumping & thresholding methodology; SBP: systolic blood pressure; TOPMed: Trans-Omics for Precision Medicine .

## Supplementary Figure 11: Distribution of highest performing single GWAS PRS and multi-PRS by race/ethnic background group in the TOPMed-BP dataset

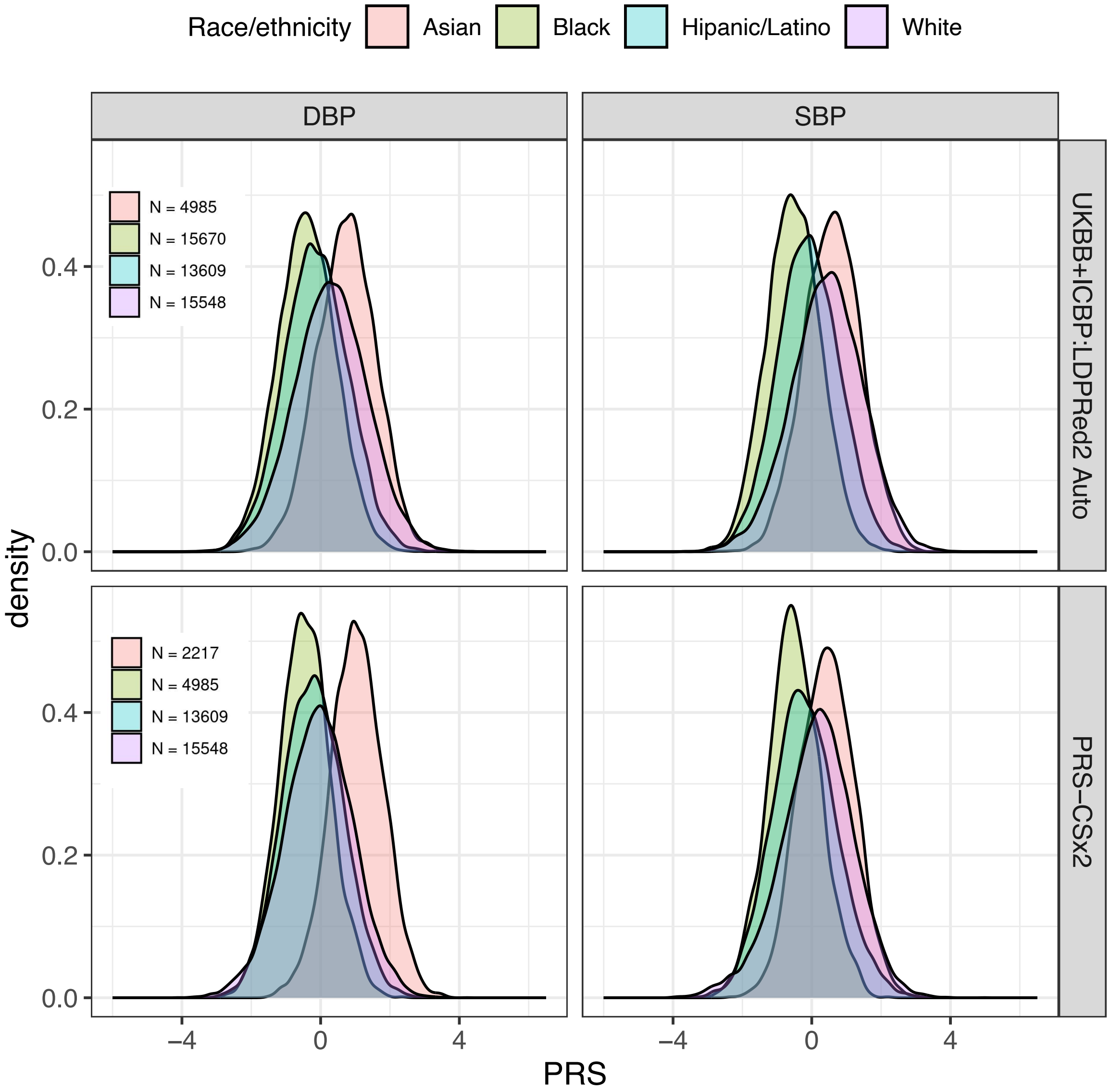


Estimated densities of the PRS-CSx2 and UKBB+ICBP LDPred2 PRSs in the TOPMed-BP dataset, stratified by race/ethnic background. Abbreviations and definitions. BP: blood pressure; DBP: diastolic blood pressure; GWAS: genome-wide association study; LDPred: Bayesian PRS method (LDPred2 denotes a specific software implementation of the LDPred algorithm); PRS-CSx: continuous shrinkage cross-population PRS method; PRS: polygenic risk score; SBP: systolic blood pressure; TOPMed: Trans-Omics for Precision Medicine .

Supplementary Figure 12: Distribution of highest performing single GWAS PRS and multi-PRS by groups defined by genetic ancestry in the TOPMed-BP dataset

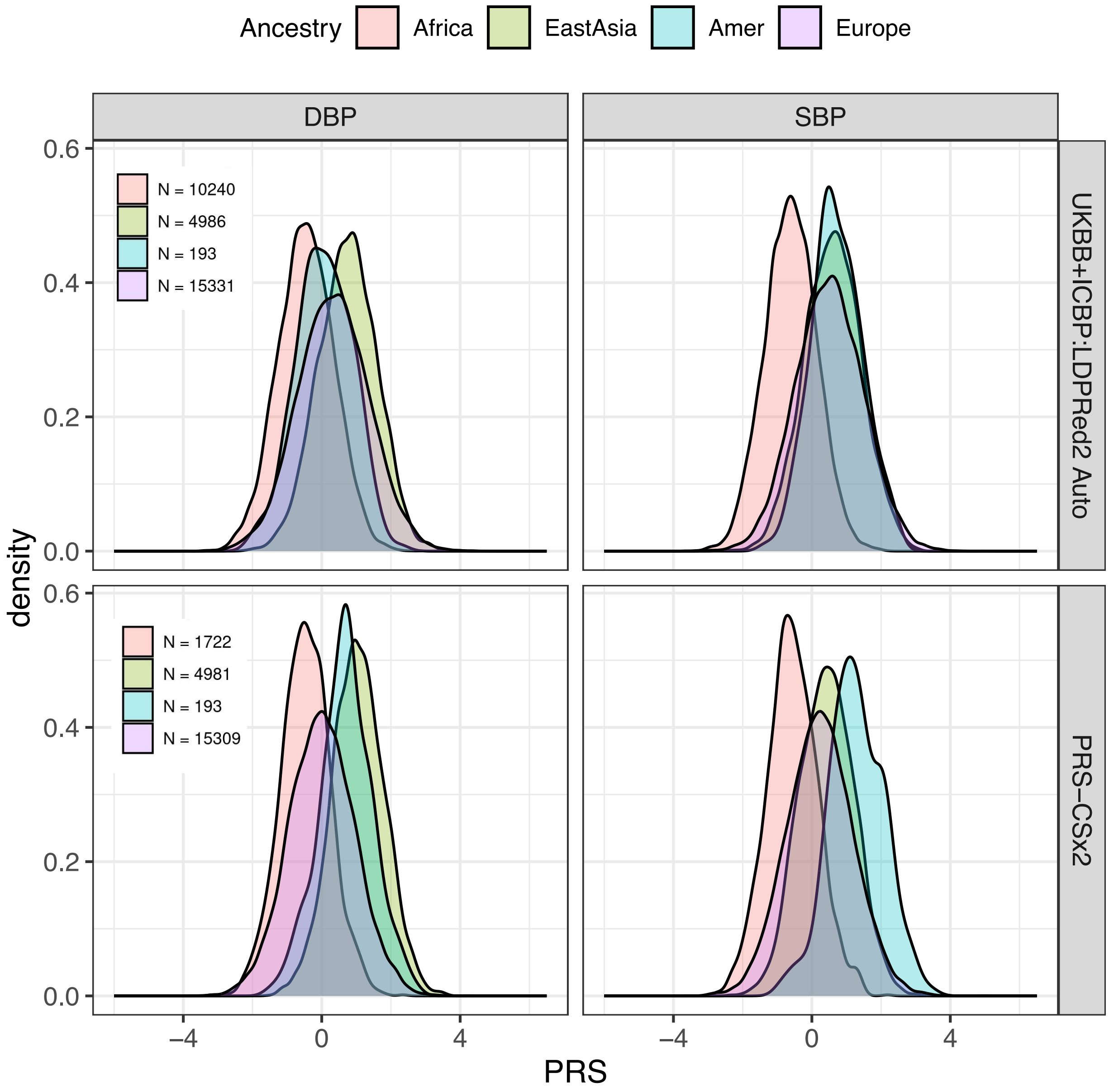


Estimated densities of the PRS-CSx2 and UKBB+ICBP LDPred2 PRSs in the TOPMed-BP dataset, stratified by groups defined by having ≥ 0.8 global proportion of a particular genetic ancestry. Abbreviations and definitions. BP: blood pressure; DBP: diastolic blood pressure; GWAS: genome-wide association study; LDPred: Bayesian PRS method (LDPred2 denotes a specific software implementation of the LDPred algorithm); PRS-CSx: continuous shrinkage cross-population PRS method; PRS: polygenic risk score; SBP: systolic blood pressure; TOPMed: Trans-Omics for Precision Medicine .

## Supplementary Figure 13: Visualization of ancestry-specific allele frequencies of SNPs from GWAS used to guide SBP PRS construction

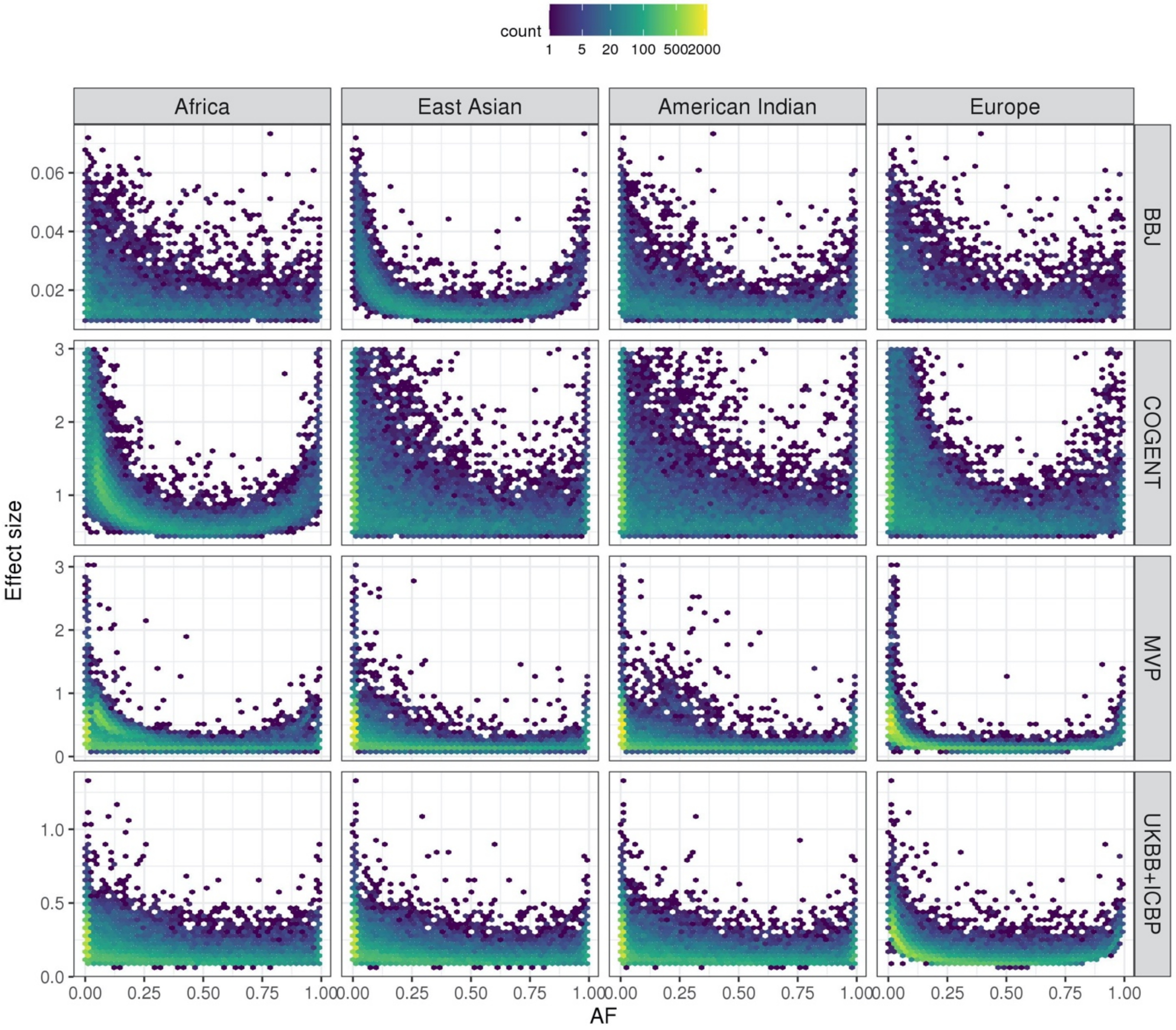


Estimated effect sizes of SNPs with p-value<0.01 in each of the SBP GWAS used in the manuscript, against estimated ancestry-specific allele frequencies (AF), estimated using the TOPMed-BP dataset and the LAFA method. Column correspond to continental genetic ancestries used. SNPs were clumped using the TOPMed-BP dataset as a reference panel with clumping parameters distance =1000Kb and $R^2$=0.1. Abbreviations and definitions. BBJ: Biobank Japan; BP: blood pressure; GWAS: genome-wide association study; MVP: Million Veteran Program; PRS: polygenic risk score; SBP: systolic blood pressure; TOPMed: Trans-Omics for Precision Medicine. LAFA: local ancestry frequency estimation; UKBB+ICBP: United Kingdom biobank and the International Consortium of Blood Pressure.

## Supplementary Figure 14: Visualization of ancestry-specific allele frequencies of SNPs from GWAS used to guide DBP PRS construction

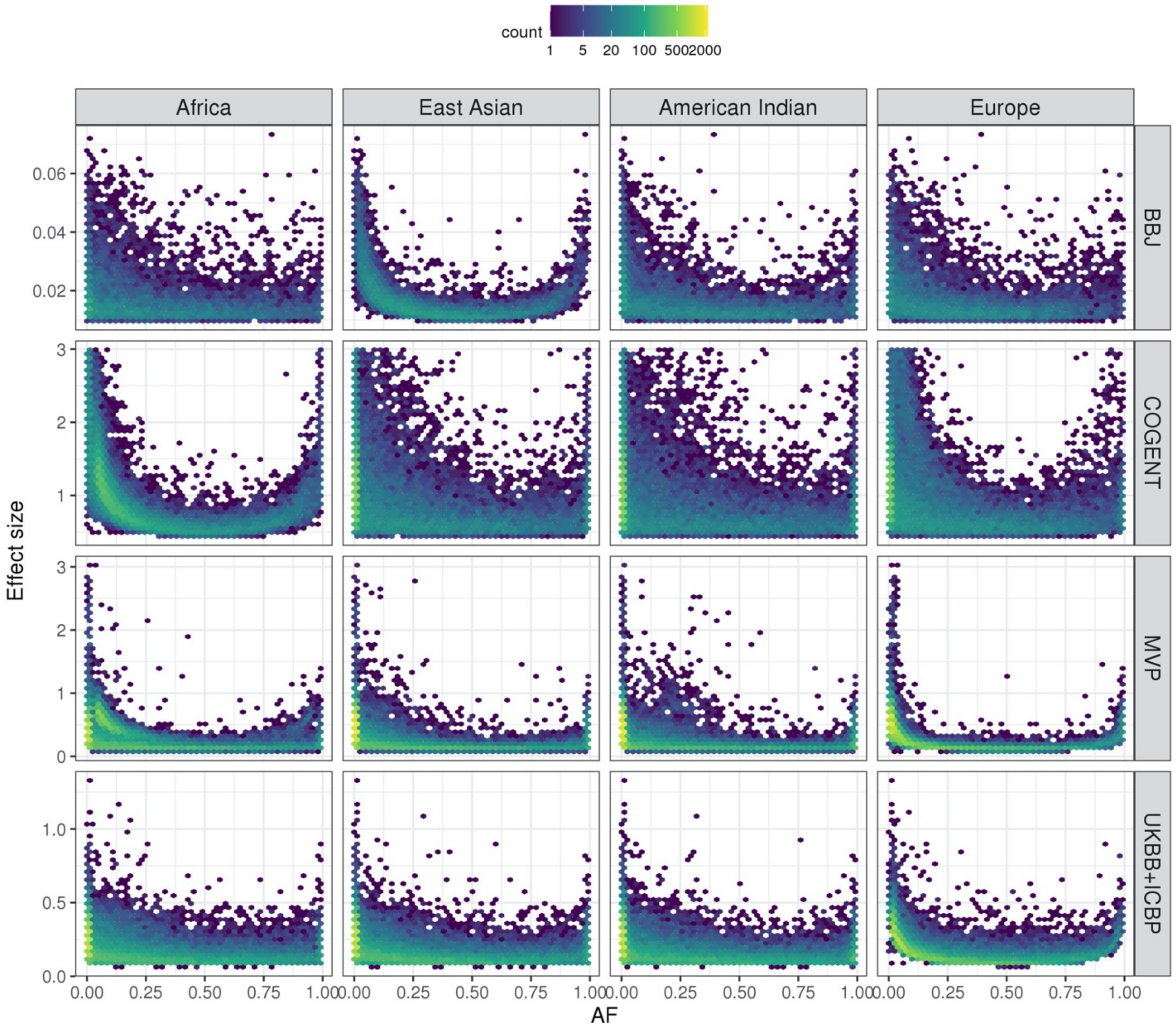


Estimated effect sizes of SNPs with p-value<0.01 in each of the DBP GWAS used in the manuscript, against estimated ancestry-specific allele frequencies (AF), estimated using the TOPMed-BP dataset and the LAFA method. Column correspond to continental genetic ancestries used. SNPs were clumped using the TOPMed-BP dataset as a reference panel with clumping parameters distance =1000Kb and $R^2$=0.1. Abbreviations and definitions. BBJ: Biobank Japan; BP: blood pressure; DBP: diastolic blood pressure; GWAS: genome-wide association study; MVP: Million Veteran Program; PRS: polygenic risk score; TOPMed: Trans-Omics for Precision Medicine. LAFA: local ancestry frequency estimation; UKBB+ICBP: United Kingdom biobank and the International Consortium of Blood Pressure.

Supplementary Figure 15: Association of SBP and DBP PRS with BP measures among All of Us individuals, stratified by groups defined by combination of self-reported race/ethnicity and genetic similarity

| | N | BETA [95% CI] | P |
|---|---|---|---|
| *Systolic blood pressure* | | | |
| EUR | 38236 | 3.79 [3.60;3.98] | 3.59e−345 |
| AFR | 18876 | 3.05 [2.67;3.43] | 1.02e−54 |
| AMR | 14355 | 3.92 [3.61;4.23] | 2.04e−132 |
| EAS | 1881 | 3.03 [2.16;3.90] | 1.21e−11 |
| All | 74290 | 3.62 [3.47;3.77] | 2.65e−508 |
| *Diastolic blood pressure* | | | |
| EUR | 38236 | 2.43 [2.31;2.56] | 1.67e−311 |
| AFR | 18876 | 2.09 [1.83;2.34] | 2.15e−57 |
| AMR | 14355 | 2.73 [2.51;2.95] | 3.27e−128 |
| EAS | 1881 | 2.19 [1.53;2.85] | 9.61e−11 |
| All | 74290 | 2.41 [2.31;2.50] | 9.03e−408 |

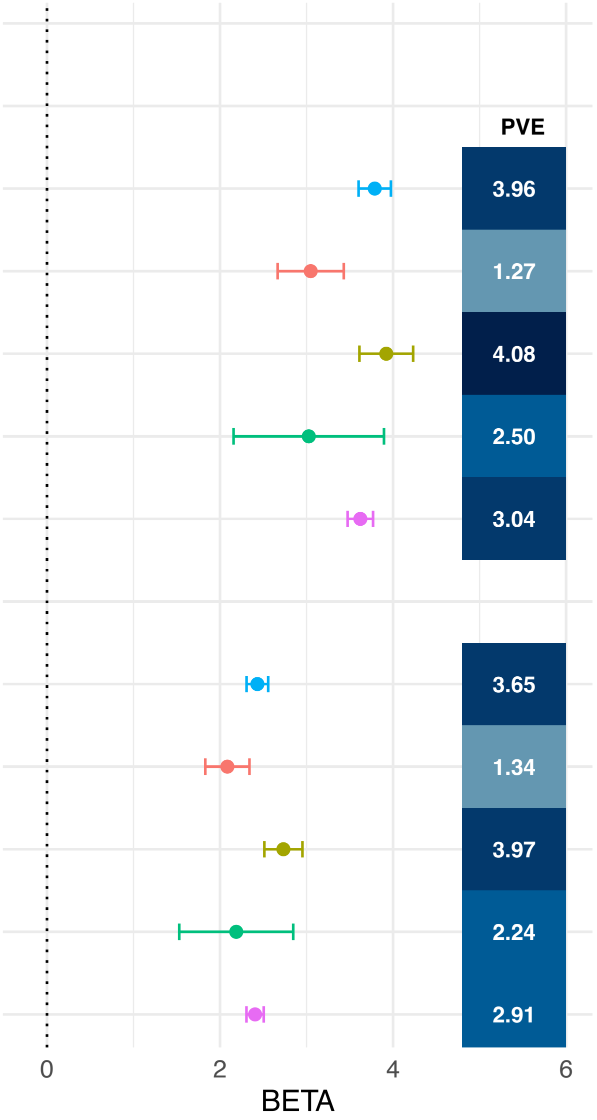


The figure visualizes the estimated associations (betas in units of mmHg per 1 SD increase in the PRS, standardized according to TOPMed; provided numerically and visualized as points in the forest plot) and 95% confidence intervals (CIs; provided numerically and visualized as the intervals in the forest plot) of the best performing SBP and DBP PRS as selected in the TOPMed dataset (PRS-CSx2), in All of Us, stratified by genetic ancestry. The figure also provides the sample sizes, in each stratum, association p-values computed based on a two-sided 1-degree of freedom Wald test, and PVEs. Analyses were adjusted for age, sex at birth, BMI, self-reported race/ethnicity, and the first 10 PCs of genetic data. Abbreviations and definitions. AFR: African ancestry; AMR: Latino/admixed American; DBP: diastolic blood pressure; EAS: East Asian; EUR: European ancestry; PRS-CSx: continuous shrinkage cross-population PRS method; PRS: polygenic risk score; SBP: systolic blood pressure; TOPMed: Trans-Omics for Precision Medicine.

## Supplementary Figure 16: Association of SBP and DBP PRS with BP measures among individuals who do not use antihypertensive medications, and stratified by race/ethnic background in the All of Us dataset

| | N | BETA [95% CI] | P |
|---|---|---|---|
| *Systolic blood pressure* | | | |
| Black | 13704 | 2.83 [2.42;3.23] | 1.08e−41 |
| Asian | 2237 | 2.92 [2.18;3.66] | 2.10e−14 |
| White | 27845 | 3.33 [3.14;3.53] | 6.86e−246 |
| Hispanic/Latino | 12774 | 3.76 [3.46;4.05] | 9.14e−136 |
| All | 56560 | 3.26 [3.11;3.42] | 5.72e−393 |
| *Diastolic blood pressure* | | | |
| Black | 13704 | 2.09 [1.80;2.38] | 2.02e−44 |
| Asian | 2237 | 2.01 [1.52;2.51] | 3.37e−15 |
| White | 27845 | 2.24 [2.11;2.37] | 2.01e−230 |
| Hispanic/Latino | 12774 | 2.41 [2.21;2.62] | 2.10e−112 |
| All | 56560 | 2.21 [2.11;2.32] | 6.67e−370 |

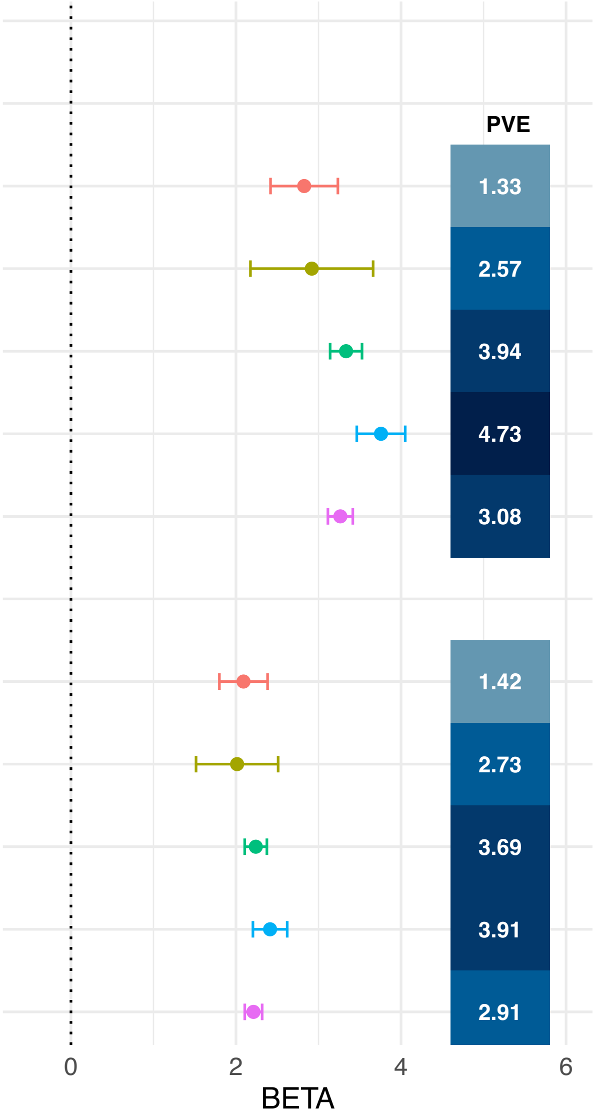


The figure visualizes the estimated associations (betas in units of mmHg per 1 SD increase in the PRS, standardized according to TOPMed; provided numerically and visualized as points in the forest plot) and 95% confidence intervals (CIs; provided numerically and visualized as the intervals in the forest plot) of the best performing SBP and DBP PRS as selected in the TOPMed dataset (PRS-CSx2), in All of Us participants who do not use antihypertensive medications, stratified by race/ethnic background. The figure also provides the sample sizes, in each stratum, association p-values computed based on a two-sided 1-degree of freedom Wald test, and PVEs. Analyses were adjusted for age, sex at birth, BMI, self-reported race/ethnicity, and the first 10 PCs of genetic data. Abbreviations and definitions. BMI: body mass index; BP: blood pressure; CI: confidence interval; DBP: diastolic blood pressure; PC: principal component; PRS-CSx: continuous shrinkage cross-population PRS method; PRS: polygenic risk score; PVE: percent variance explained; SBP: systolic blood pressure; SD: standard deviation; TOPMed: Trans-Omics for Precision Medicine.

## Supplementary Figure 17: Association of SBP and DBP PRS with BP measures among individuals who use antihypertensive medications, and stratified by race/ethnic background in the All of Us dataset

| | N | BETA [95% CI] | P |
|---|---|---|---|
| *Systolic blood pressure* | | | |
| Black | 3551 | 2.59 [ 1.66;3.52] | 5.18e−08 |
| Asian | 400 | 1.38 [−0.78;3.54] | 2.12e−01 |
| White | 10018 | 2.76 [ 2.39;3.13] | 4.19e−47 |
| Hispanic/Latino | 3761 | 2.93 [ 2.28;3.57] | 7.10e−19 |
| All | 17730 | 2.71 [ 2.41;3.02] | 4.36e−67 |
| *Diastolic blood pressure* | | | |
| Black | 3551 | 1.75 [ 1.13;2.37] | 3.04e−08 |
| Asian | 400 | 1.29 [−0.01;2.59] | 5.18e−02 |
| White | 10018 | 1.71 [ 1.48;1.95] | 1.19e−44 |
| Hispanic/Latino | 3761 | 2.15 [ 1.72;2.57] | 6.48e−23 |
| All | 17730 | 1.81 [ 1.61;2.00] | 4.44e−71 |

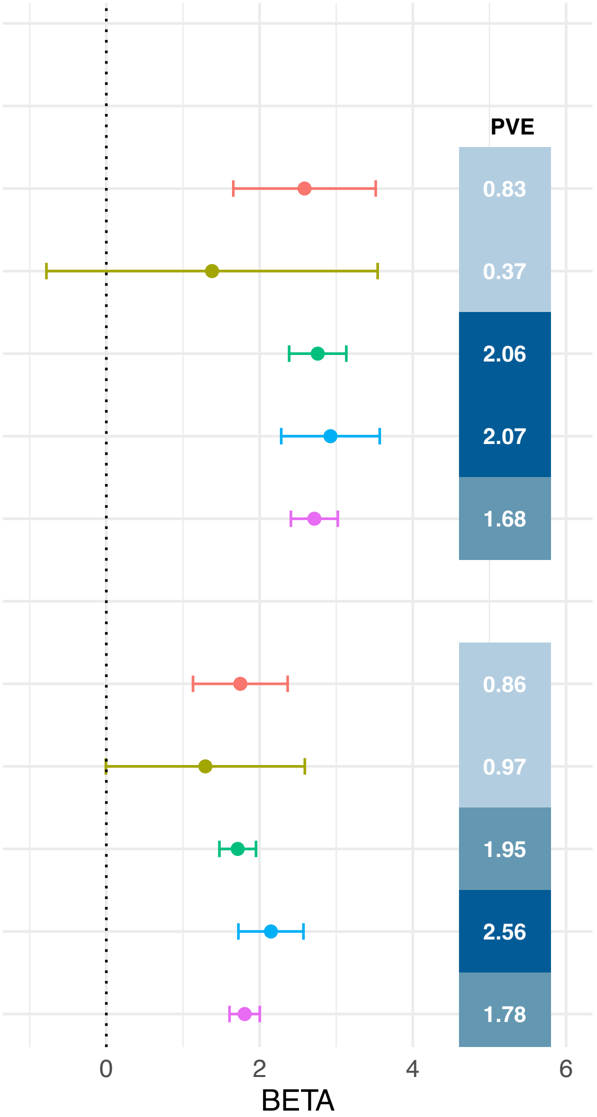


The figure visualizes the estimated associations (betas in units of mmHg per 1 SD increase in the PRS, standardized according to TOPMed; provided numerically and visualized as points in the forest plot) and 95% confidence intervals (CIs; provided numerically and visualized as the intervals in the forest plot) of the best performing SBP and DBP PRS as selected in the TOPMed dataset (PRS-CSx2), in All of Us participants who use antihypertensive medications, stratified by race/ethnic background. The figure also provides the sample sizes, in each stratum, association p-values computed based on a two-sided 1-degree of freedom Wald test, and PVEs. Analyses were adjusted for age, sex at birth, BMI, self-reported race/ethnicity, and the first 10 PCs of genetic data. Abbreviations and definitions. BMI: body mass index; BP: blood pressure; CI: confidence interval; DBP: diastolic blood pressure; PC: principal component; PRS-CSx: continuous shrinkage cross-population PRS method; PRS: polygenic risk score; PVE: percent variance explained; SBP: systolic blood pressure; SD: standard deviation; TOPMed: Trans-Omics for Precision Medicine.

## Supplementary Figure 18: Association of BP PRSs with BP measures by strata of age, obesity, and sex in White participants from All of Us

| | N | BETA [95% CI] | P |
|---|---|---|---|
| *Systolic blood pressure* | | | |
| *Age range* | | | |
| ≤ 40 | 9627 | 2.77 [2.47;3.07] | 2.40e-70 |
| 41-60 | 12555 | 4.31 [3.98;4.64] | 3.10e-140 |
| ≥ 61 | 15681 | 3.84 [3.51;4.16] | 3.96e-117 |
| *Obesity* | | | |
| Non-Obese | 24839 | 3.92 [3.69;4.15] | 5.29e-247 |
| Obese | 11496 | 3.49 [3.13;3.84] | 1.51e-79 |
| *Sex at birth* | | | |
| Male | 15004 | 3.46 [3.16;3.76] | 1.02e-110 |
| Female | 22859 | 3.96 [3.72;4.20] | 6.91e-225 |
| *Diastolic blood pressure* | | | |
| *Age range* | | | |
| ≤ 40 | 9627 | 2.35 [2.11;2.59] | 1.42e-80 |
| 41-60 | 12555 | 2.77 [2.55;2.99] | 8.23e-126 |
| ≥ 61 | 15681 | 2.30 [2.10;2.50] | 6.27e-112 |
| *Obesity* | | | |
| Non-Obese | 24839 | 2.63 [2.47;2.78] | 6.05e-245 |
| Obese | 11496 | 2.17 [1.93;2.41] | 6.31e-69 |
| *Sex at birth* | | | |
| Male | 15004 | 2.12 [1.91;2.32] | 2.22e-89 |
| Female | 22859 | 2.66 [2.49;2.82] | 1.08e-224 |

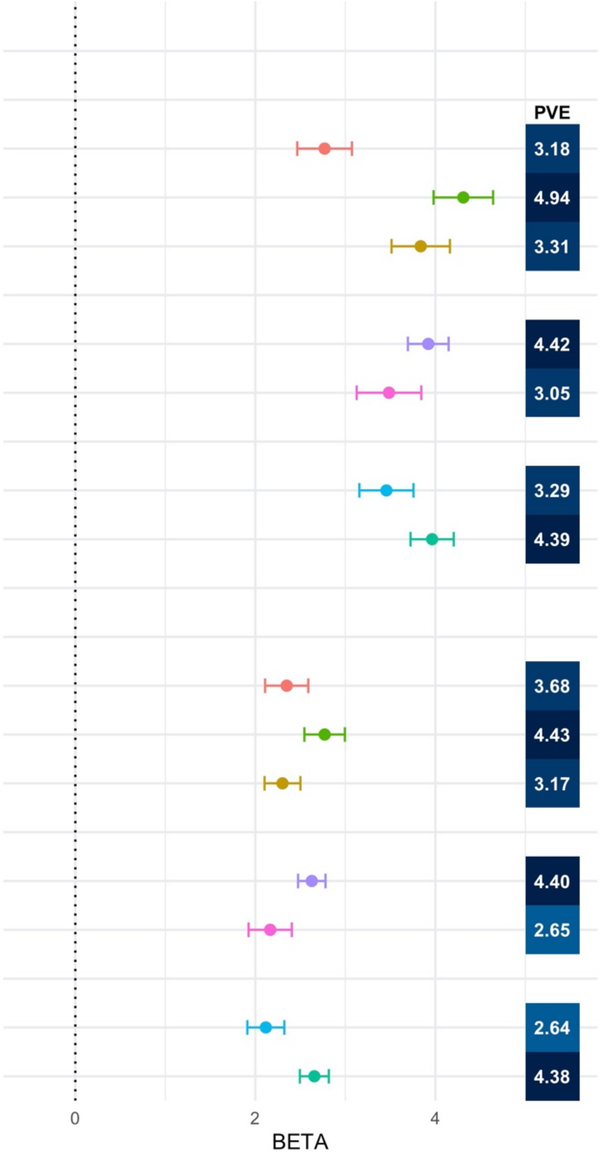


The figure visualizes the estimated associations (betas in units of mmHg per 1 SD increase in the PRS, standardized according to TOPMed; provided numerically and visualized as points in the forest plot) and 95% confidence intervals (CIs; provided numerically and visualized as the intervals in the forest plot) of the best performing SBP and DBP PRS as selected in the TOPMed dataset (PRS-CSx2), in All of Us self-reported White individuals, stratified by strata of age, obesity, and sex. The figure also provides the sample sizes, in each stratum, association p-values computed based on a two-sided 1-degree of freedom Wald test, and PVEs. Analyses were adjusted for age, sex, BMI, self-reported race/ethnicity, and the first 10 PCs of genetic data. Abbreviations and definitions. BMI: body mass index; BP: blood pressure; CI: confidence interval; DBP: diastolic blood pressure; PC: principal component; PRS-CSx: continuous shrinkage cross-population PRS method; PRS: polygenic risk score; PVE: percent variance explained; SBP: systolic blood pressure; SD: standard deviation; TOPMed: Trans-Omics for Precision Medicine.

## Supplementary Figure 19: Association of BP PRSs with BP measures stratified by age, obesity, and sex in Black participants from All of Us

| | N | BETA [95% CI] | P |
|---|---|---|---|
| *Systolic blood pressure* | | | |
| *Age range* | | | |
| ≤ 40 | 5494 | 2.72 [2.11;3.33] | 2.85e-18 |
| 41-60 | 8205 | 3.54 [2.92;4.15] | 2.18e-29 |
| ≥ 61 | 3556 | 2.50 [1.55;3.44] | 2.32e-07 |
| *Obesity* | | | |
| Non-Obese | 9254 | 3.14 [2.62;3.67] | 3.38e-31 |
| Obese | 7294 | 2.98 [2.34;3.61] | 4.70e-20 |
| *Sex at birth* | | | |
| Male | 7887 | 2.85 [2.28;3.43] | 1.85e-22 |
| Female | 9368 | 3.36 [2.81;3.91] | 1.33e-32 |
| *Diastolic blood pressure* | | | |
| *Age range* | | | |
| ≤ 40 | 5494 | 2.25 [1.76;2.75] | 7.28e-19 |
| 41-60 | 8205 | 2.39 [1.98;2.81] | 1.21e-29 |
| ≥ 61 | 3556 | 1.65 [1.04;2.25] | 9.45e-08 |
| *Obesity* | | | |
| Non-Obese | 9254 | 2.21 [1.84;2.59] | 7.69e-31 |
| Obese | 7294 | 2.27 [1.84;2.71] | 2.42e-24 |
| *Sex at birth* | | | |
| Male | 7887 | 2.20 [1.79;2.62] | 5.05e-25 |
| Female | 9368 | 2.29 [1.92;2.66] | 7.11e-33 |

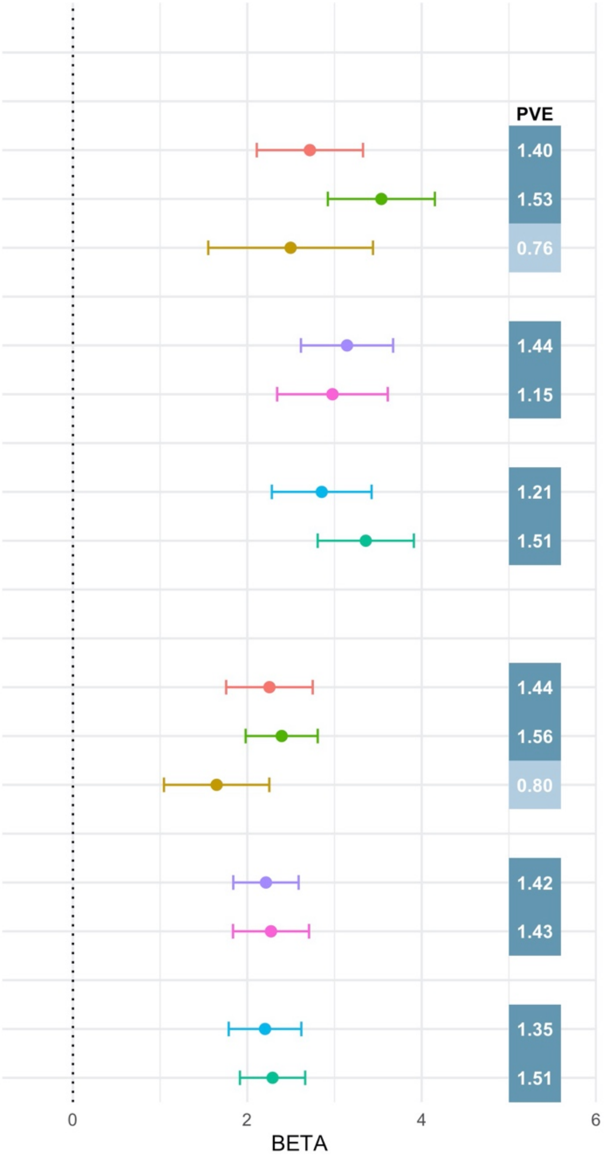


The figure visualizes the estimated associations (betas in units of mmHg per 1 SD increase in the PRS, standardized according to TOPMed; provided numerically and visualized as points in the forest plot) and 95% confidence intervals (CIs; provided numerically and visualized as the intervals in the forest plot) of the best performing SBP and DBP PRS as selected in the TOPMed dataset (PRS-CSx2), in All of Us self-reported Black individuals, stratified by strata of age, obesity, and sex. The figure also provides the sample sizes, in each stratum, association p-values computed based on a two-sided 1-degree of freedom Wald test, and PVEs. Analyses were adjusted for age, sex, BMI, self-reported race/ethnicity, and the first 10 PCs of genetic data. Abbreviations and definitions. BMI: body mass index; BP: blood pressure; CI: confidence interval; DBP: diastolic blood pressure; PC: principal component; PRS-CSx: continuous shrinkage cross-population PRS method; PRS: polygenic risk score; PVE: percent variance explained; SBP: systolic blood pressure; SD: standard deviation; TOPMed: Trans-Omics for Precision Medicine.

## Supplementary Figure 20: Association of BP PRSs with BP measures stratified by age, obesity, and sex in Hispanic/Latino participants from All of Us

| | N | BETA [95% CI] | P |
|---|---|---|---|
| *Systolic blood pressure* | | | |
| *Age range* | | | |
| ≤ 40 | 7224 | 3.21 [2.82;3.60] | 2.16e-57 |
| 41-60 | 6278 | 4.75 [4.24;5.27] | 2.53e-72 |
| ≥ 61 | 3033 | 3.91 [3.12;4.71] | 9.24e-22 |
| *Obesity* | | | |
| Non-Obese | 9103 | 4.13 [3.75;4.52] | 1.05e-95 |
| Obese | 6541 | 3.73 [3.25;4.22] | 2.84e-50 |
| *Sex at birth* | | | |
| Male | 5215 | 3.72 [3.19;4.26] | 9.54e-42 |
| Female | 11320 | 4.10 [3.75;4.45] | 1.08e-113 |
| *Diastolic blood pressure* | | | |
| *Age range* | | | |
| ≤ 40 | 7224 | 2.44 [2.14;2.74] | 1.47e-56 |
| 41-60 | 6278 | 3.03 [2.70;3.37] | 3.78e-68 |
| ≥ 61 | 3033 | 2.02 [1.55;2.49] | 4.08e-17 |
| *Obesity* | | | |
| Non-Obese | 9103 | 2.57 [2.30;2.83] | 8.15e-79 |
| Obese | 6541 | 2.55 [2.22;2.88] | 5.14e-51 |
| *Sex at birth* | | | |
| Male | 5215 | 2.40 [2.03;2.76] | 1.01e-36 |
| Female | 11320 | 2.66 [2.42;2.90] | 3.61e-103 |

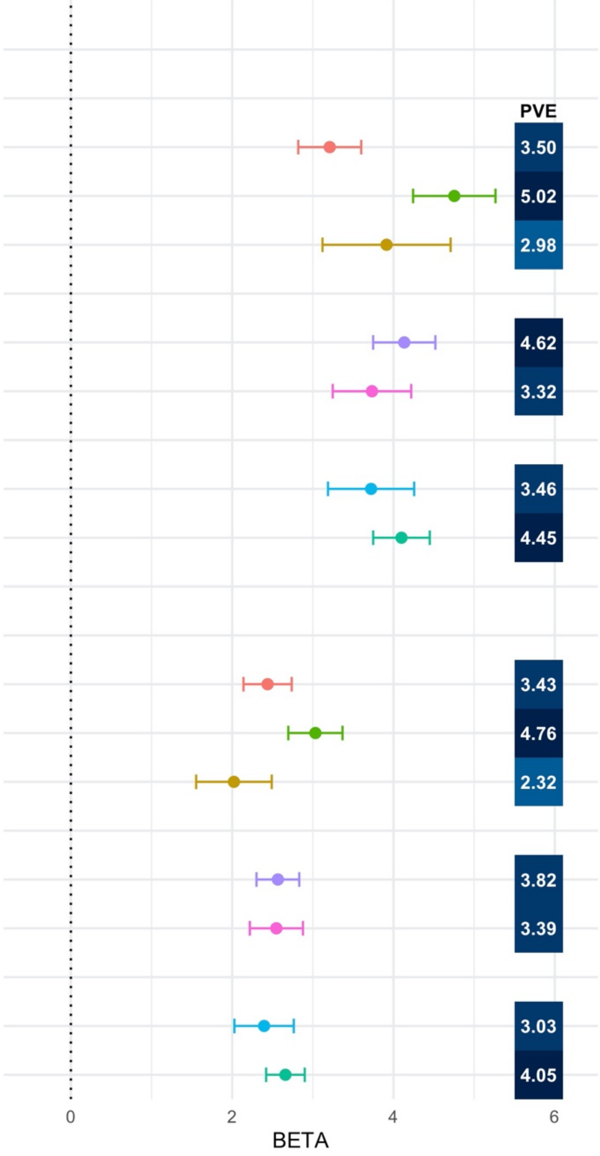


The figure visualizes the estimated associations (betas in units of mmHg per 1 SD increase in the PRS, standardized according to TOPMed; provided numerically and visualized as points in the forest plot) and 95% confidence intervals (CIs; provided numerically and visualized as the intervals in the forest plot) of the best performing SBP and DBP PRS as selected in the TOPMed dataset (PRS-CSx2), in All of Us self-reported Hispanic/Latino individuals, stratified by strata of age, obesity, and sex. The figure also provides the sample sizes, in each stratum, association p-values computed based on a two-sided 1-degree of freedom Wald test, and PVEs. Analyses were adjusted for age, sex, BMI, self-reported race/ethnicity, and the first 10 PCs of genetic data. Abbreviations and definitions. BMI: body mass index; BP: blood pressure; CI: confidence interval; DBP: diastolic blood pressure; PC: principal component; PRS-CSx: continuous shrinkage cross-population PRS method; PRS: polygenic risk score; PVE: percent variance explained; SBP: systolic blood pressure; SD: standard deviation; TOPMed: Trans-Omics for Precision Medicine.

## Supplementary Figure 21: Association of BP PRSs with BP measures stratified by age, obesity, and sex in Asian participants from All of Us

| | N | BETA [95% CI] | P |
|---|---|---|---|
| *Systolic blood pressure* | | | |
| *Age range* | | | |
| ≤ 40 | 1350 | 2.57 [ 1.69;3.46] | 1.44e-08 |
| 41-60 | 778 | 4.26 [ 2.69;5.70] | 1.28e-07 |
| ≥ 61 | 509 | 2.22 [ 0.10;4.35] | 4.11e-02 |
| *Obesity* | | | |
| Non-Obese | 2323 | 2.94 [ 2.14;3.75] | 1.11e-12 |
| Obese | 248 | 4.16 [ 1.45;5.70] | 2.90e-03 |
| *Sex at birth* | | | |
| Male | 1059 | 2.70 [ 1.48;3.93] | 1.70e-05 |
| Female | 1578 | 3.36 [ 2.38;4.33] | 2.09e-11 |
| *Diastolic blood pressure* | | | |
| *Age range* | | | |
| ≤ 40 | 1350 | 2.16 [ 1.53;2.79] | 2.97e-11 |
| 41-60 | 778 | 2.48 [ 1.49;3.47] | 1.10e-06 |
| ≥ 61 | 509 | 1.54 [ 0.26;2.81] | 1.87e-02 |
| *Obesity* | | | |
| Non-Obese | 2323 | 2.14 [ 1.62;2.65] | 6.38e-16 |
| Obese | 248 | 1.74 [-0.20;3.69] | 7.96e-02 |
| *Sex at birth* | | | |
| Male | 1059 | 1.83 [ 1.04;2.62] | 6.06e-06 |
| Female | 1578 | 2.30 [ 1.67;2.94] | 1.83e-12 |

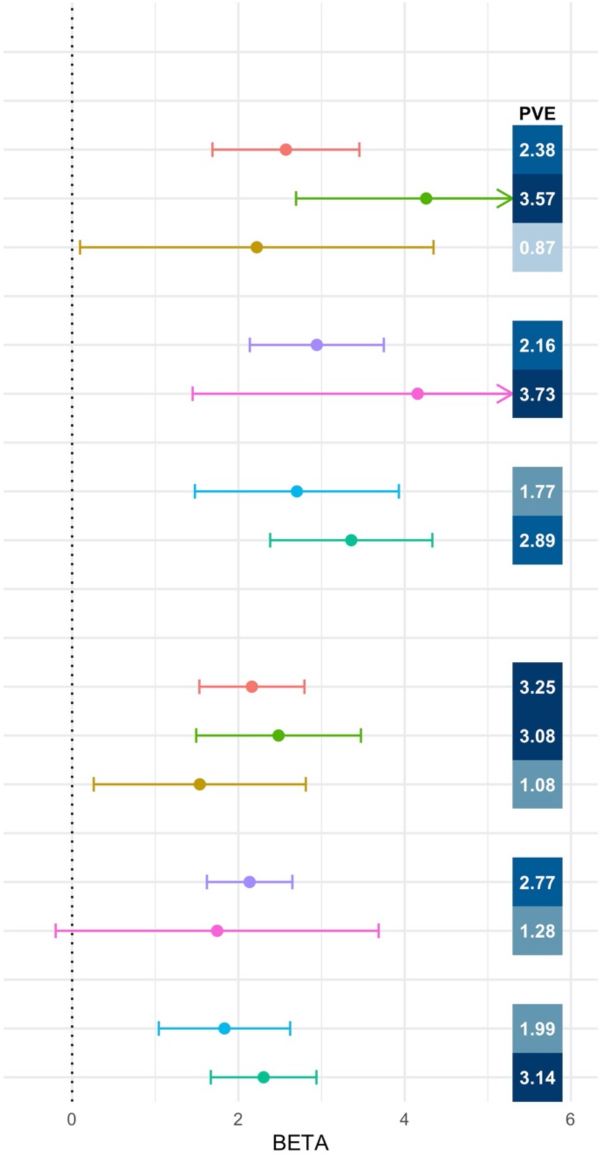


The figure visualizes the estimated associations (betas in units of mmHg per 1 SD increase in the PRS, standardized according to TOPMed; provided numerically and visualized as points in the forest plot) and 95% confidence intervals (CIs; provided numerically and visualized as the intervals in the forest plot) of the best performing SBP and DBP PRS as selected in the TOPMed dataset (PRS-CSx2), in All of Us self-reported Asian individuals, stratified by strata of age, obesity, and sex. The figure also provides the sample sizes, in each stratum, association p-values computed based on a two-sided 1-degree of freedom Wald test, and PVEs. Analyses were adjusted for age, sex, BMI, self-reported race/ethnicity, and the first 10 PCs of genetic data. Abbreviations and definitions. BMI: body mass index; BP: blood pressure; CI: confidence interval; DBP: diastolic blood pressure; PC: principal component; PRS-CSx: continuous shrinkage cross-population PRS method; PRS: polygenic risk score; PVE: percent variance explained; SBP: systolic blood pressure; SD: standard deviation; TOPMed: Trans-Omics for Precision Medicine.

## Supplementary  Figure 22: Association of SBP and DBP PRS with BP measures among individuals who do not use antihypertensive medications, by strata of age, obesity, and sex in All of Us

| | N | BETA [95% CI] | P |
|---|---|---|---|
| ***Systolic blood pressure*** | | | |
| *Age range* | | | |
| ≤ 40 | 20114 | 2.55 [2.34;2.76] | 6.31e-122 |
| 41-60 | 21230 | 3.76 [3.49;4.02] | 3.63e-169 |
| ≥ 61 | 15216 | 3.32 [3.00;3.64] | 3.15e-91 |
| *Obesity* | | | |
| Non-Obese | 36076 | 3.34 [3.16;3.53] | 5.16e-277 |
| Obese | 18102 | 3.09 [2.80;3.37] | 3.61e-100 |
| *Sex at birth* | | | |
| Male | 22336 | 2.99 [2.75;3.24] | 5.17e-127 |
| Female | 34224 | 3.44 [3.25;3.63] | 5.97e-269 |
| ***Diastolic blood pressure*** | | | |
| *Age range* | | | |
| ≤ 40 | 20114 | 2.16 [1.99;2.33] | 2.27e-132 |
| 41-60 | 21230 | 2.49 [2.30;2.67] | 3.88e-157 |
| ≥ 61 | 15216 | 1.91 [1.71;2.11] | 1.00e-78 |
| *Obesity* | | | |
| Non-Obese | 36076 | 2.27 [2.14;2.40] | 1.15e-260 |
| Obese | 18102 | 2.13 [1.93;2.32] | 2.45e-99 |
| *Sex at birth* | | | |
| Male | 22336 | 1.95 [1.78;2.12] | 1.53e-107 |
| Female | 34224 | 2.37 [2.24;2.51] | 5.40e-265 |

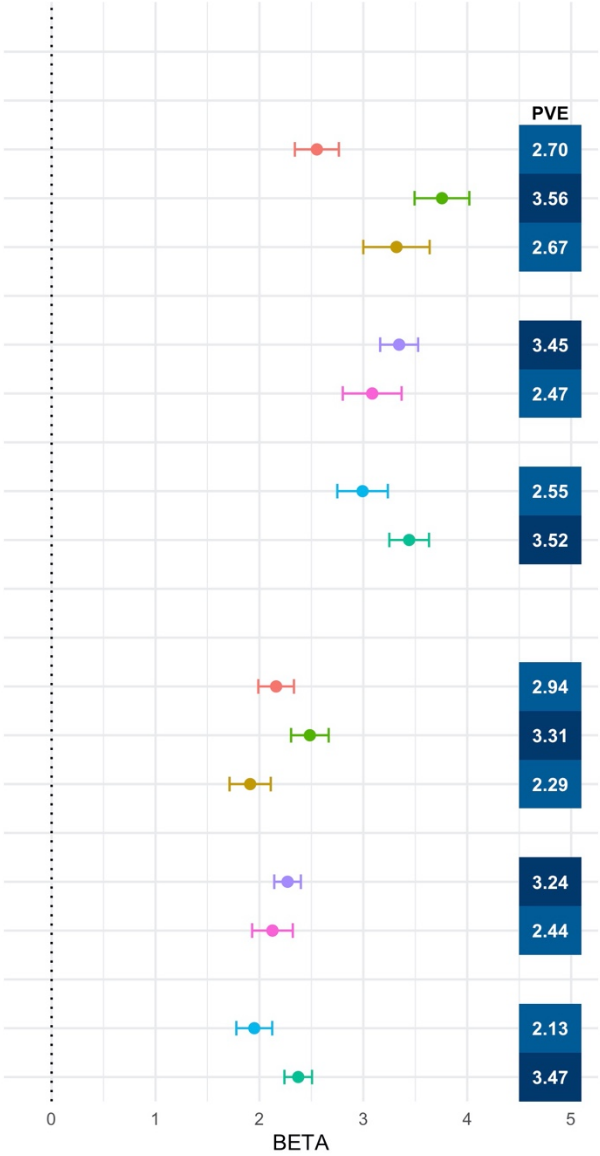


The figure visualizes the estimated associations (betas in units of mmHg per 1 SD increase in the PRS, standardized according to TOPMed; provided numerically and visualized as points in the forest plot) and 95% confidence intervals (CIs; provided numerically and visualized as the intervals in the forest plot) of the best performing SBP and DBP PRS as selected in the TOPMed dataset (PRS-CSx2), in All of Us self-reported Asian individuals, stratified by strata of age, obesity, and sex. The figure also provides the sample sizes, in each stratum, association p-values computed based on a two-sided 1-degree of freedom Wald test, and PVEs. Analyses were adjusted for age, sex, BMI, self-reported race/ethnicity, and the first 10 PCs of genetic data. Abbreviations and definitions. BMI: body mass index; BP: blood pressure; CI: confidence interval; DBP: diastolic blood pressure; PC: principal component; PRS-CSx: continuous shrinkage cross-population PRS method; PRS: polygenic risk score; PVE: percent variance explained; SBP: systolic blood pressure; SD: standard deviation; TOPMed: Trans-Omics for Precision Medicine.

Supplementary Figure 23: Association of SBP and DBP PRS with BP measures among individuals who use antihypertensive medications, by strata of age, obesity, and sex in participants from All of Us

| | N | BETA [95% CI] | P |
|---|---|---|---|
| *Systolic blood pressure* | | | |
| *Age range* | | | |
| ≤ 40 | 3581 | 2.62 [2.03;3.22] | 7.57e-18 |
| 41-60 | 6586 | 2.88 [2.35;3.41] | 4.95e-26 |
| ≥ 61 | 7563 | 2.47 [2.00;2.94] | 9.78e-25 |
| *Obesity* | | | |
| Non-Obese | 9443 | 2.97 [2.56;3.38] | 7.61e-46 |
| Obese | 7477 | 2.32 [1.83;2.81] | 1.30e-20 |
| *Sex at birth* | | | |
| Male | 6829 | 2.28 [1.79;2.77] | 1.31e-19 |
| Female | 10901 | 2.94 [2.55;3.33] | 1.34e-49 |
| *Diastolic blood pressure* | | | |
| *Age range* | | | |
| ≤ 40 | 3581 | 2.24 [1.78;2.71] | 5.35e-21 |
| 41-60 | 6586 | 1.92 [1.58;2.26] | 3.09e-28 |
| ≥ 61 | 7563 | 1.58 [1.30;1.86] | 8.90e-28 |
| *Obesity* | | | |
| Non-Obese | 9443 | 1.93 [1.67;2.19] | 3.40e-47 |
| Obese | 7477 | 1.64 [1.32;1.96] | 6.83e-24 |
| *Sex at birth* | | | |
| Male | 6829 | 1.52 [1.19;1.85] | 7.54e-20 |
| Female | 10901 | 1.95 [1.70;2.20] | 2.77e-53 |

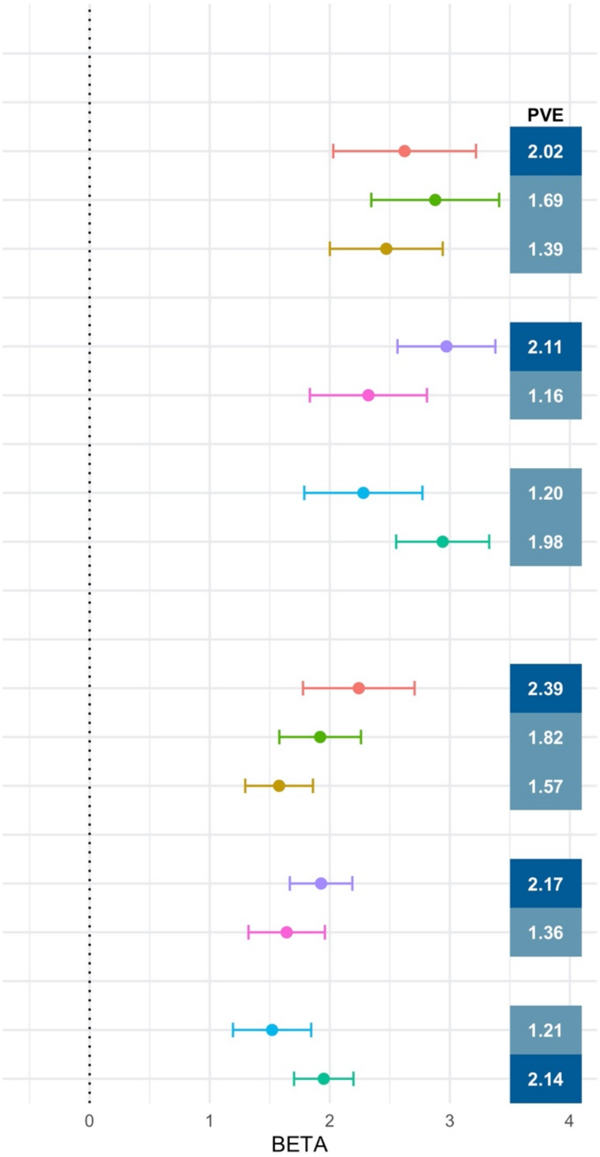


The figure visualizes the estimated associations (betas in units of mmHg per 1 SD increase in the PRS, standardized according to TOPMed; provided numerically and visualized as points in the forest plot) and 95% confidence intervals (CIs; provided numerically and visualized as the intervals in the forest plot) of the best performing SBP and DBP PRS as selected in the TOPMed dataset (PRS-CSx2), in All of Us self-reported Asian individuals, stratified by strata of age, obesity, and sex. The figure also provides the sample sizes, in each stratum, association p-values computed based on a two-sided 1-degree of freedom Wald test, and PVEs. Analyses were adjusted for age, sex, BMI, self-reported race/ethnicity, and the first 10 PCs of genetic data. Abbreviations and definitions. BMI: body mass index; BP: blood pressure; CI: confidence interval; DBP: diastolic blood pressure; PC: principal component; PRS-CSx: continuous shrinkage cross-population PRS method; PRS: polygenic risk score; PVE: percent variance explained; SBP: systolic blood pressure; SD: standard deviation; TOPMed: Trans-Omics for Precision Medicine.

## Supplementary Figure 24: Association of BP and hypertension PRS with prevalent clinical outcomes in the All of Us dataset

| | Cases | Controls | OR [95% CI] | P | AUC |
|---|---|---|---|---|---|
| *SBP PRS* | | | | | |
| Hypertension | 46887 | 27403 | 1.41 [1.38;1.44] | 4.37e−292 | 0.71 |
| Type II Diabetes | 4833 | 83688 | 1.12 [1.08;1.16] | 3.38e−11 | 0.65 |
| Chronic Kidney Disease | 3425 | 85096 | 1.11 [1.07;1.15] | 1.91e−07 | 0.66 |
| Coronary Artery Disease | 2880 | 85641 | 1.11 [1.06;1.15] | 2.26e−06 | 0.74 |
| Atrial Fibrillation | 3829 | 84692 | 1.07 [1.03;1.10] | 7.37e−04 | 0.74 |
| Heart Failure | 2562 | 85959 | 1.14 [1.09;1.19] | 1.18e−08 | 0.68 |
| *DBP PRS* | | | | | |
| Hypertension | 46887 | 27403 | 1.43 [1.40;1.45] | 1.36e−292 | 0.70 |
| Type II Diabetes | 4833 | 83688 | 1.08 [1.04;1.12] | 9.66e−06 | 0.66 |
| Chronic Kidney Disease | 3425 | 85096 | 1.09 [1.05;1.13] | 3.61e−05 | 0.67 |
| Coronary Artery Disease | 2880 | 85641 | 1.09 [1.04;1.13] | 1.92e−04 | 0.73 |
| Atrial Fibrillation | 3829 | 84692 | 1.07 [1.03;1.11] | 3.27e−04 | 0.75 |
| Heart Failure | 2562 | 85959 | 1.12 [1.07;1.17] | 1.65e−06 | 0.67 |
| *SBP and DBP PRS weighted sum* | | | | | |
| Hypertension | 46889 | 27417 | 1.44 [1.41;1.47] | 4.62e−321 | 0.71 |
| Type II Diabetes | 4833 | 83688 | 1.11 [1.08;1.15] | 7.77e−10 | 0.66 |
| Chronic Kidney Disease | 3425 | 85096 | 1.11 [1.06;1.15] | 3.94e−07 | 0.67 |
| Coronary Artery Disease | 2880 | 85641 | 1.10 [1.06;1.15] | 4.32e−06 | 0.74 |
| Atrial Fibrillation | 3829 | 84692 | 1.07 [1.03;1.11] | 2.91e−04 | 0.75 |
| Heart Failure | 2562 | 85959 | 1.14 [1.09;1.19] | 1.57e−08 | 0.68 |
| *HTN PRS (Previously published)* | | | | | |
| Hypertension | 46887 | 27403 | 1.27 [1.25;1.29] | 6.67e−158 | 0.70 |
| Type II Diabetes | 4833 | 83688 | 1.14 [1.10;1.17] | 9.64e−16 | 0.66 |
| Chronic Kidney Disease | 3425 | 85096 | 1.11 [1.07;1.16] | 1.65e−08 | 0.66 |
| Coronary Artery Disease | 2880 | 85641 | 1.07 [1.03;1.12] | 1.24e−03 | 0.74 |
| Atrial Fibrillation | 3829 | 84692 | 1.04 [1.00;1.08] | 2.76e−02 | 0.76 |
| Heart Failure | 2562 | 85959 | 1.14 [1.09;1.19] | 3.83e−09 | 0.67 |

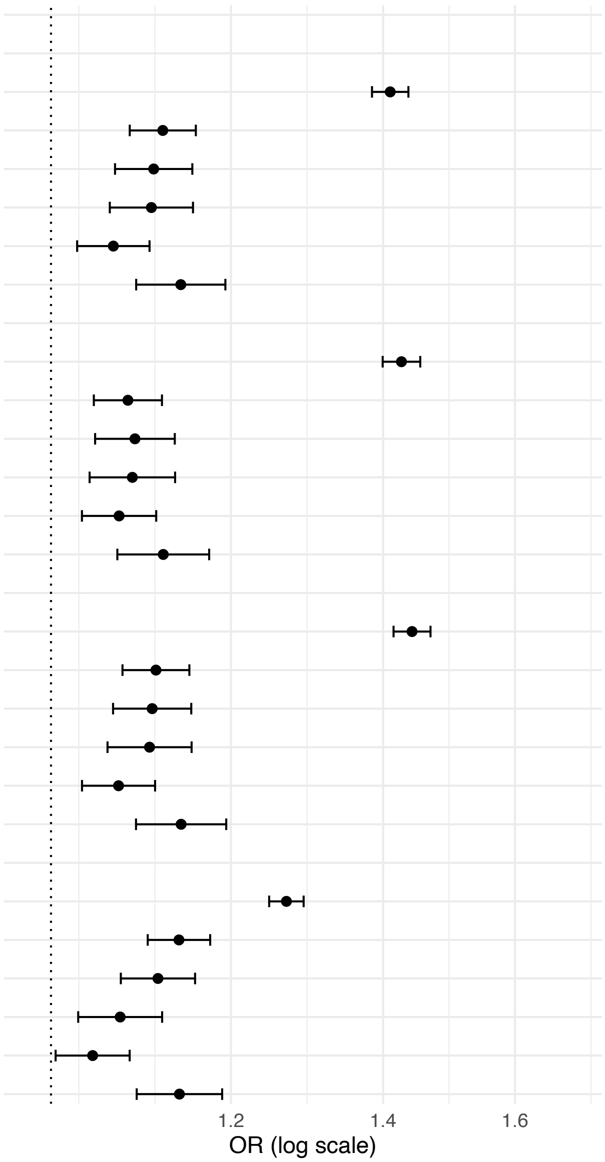


The figure visualizes the estimated associations (odds ratios; ORs; provided numerically and visualized as points in the forest plot) and 95% confidence intervals (CIs; provided numerically and visualized as the intervals in the forest plot) of the best performing SBP and DBP PRSs as selected in the TOPMed dataset (PRS-CSx2), their simple sum, and the previously-developed HTN-PRS with prevalent clinical outcomes in All of Us. The figure also provides the numbers of cases and controls for each outcome, association p-values computed based on a two-sided 1-degree of freedom Wald test, and AUCs (area under the receiver operating curve). Analyses were adjusted for age, sex, BMI, self-reported race/ethnicity, and the first 10 PCs of genetic data. Abbreviations and definitions. BMI: body mass index; BP: blood pressure; CI: confidence interval; DBP: diastolic blood pressure; PC: principal component; PRS-CSx: continuous shrinkage cross-population PRS method; PRS: polygenic risk score; PVE: percent variance explained; SBP: systolic blood pressure; SD: standard deviation; TOPMed: Trans-Omics for Precision Medicine.

## Supplementary Figure 25: Association of BP and hypertension PRS with prevalent clinical outcomes in the All of Us dataset among antihypertensive medication users

| | Cases | Controls | OR [95% CI] | P | AUC |
|---|---|---|---|---|---|
| *SBP PRS* | | | | | |
| Type II Diabetes | 3087 | 20329 | 1.04 [0.99;1.09] | 9.02e−02 | 0.65 |
| Chronic Kidney Disease | 2373 | 21043 | 1.01 [0.96;1.06] | 7.47e−01 | 0.63 |
| Coronary Artery Disease | 2011 | 21405 | 1.00 [0.95;1.06] | 9.02e−01 | 0.69 |
| Atrial Fibrillation | 2554 | 20862 | 0.99 [0.94;1.04] | 6.40e−01 | 0.68 |
| Heart Failure | 1859 | 21557 | 1.04 [0.99;1.10] | 1.23e−01 | 0.61 |
| *DBP PRS* | | | | | |
| Type II Diabetes | 3087 | 20329 | 1.04 [0.99;1.09] | 1.05e−01 | 0.65 |
| Chronic Kidney Disease | 2373 | 21043 | 0.98 [0.94;1.03] | 5.27e−01 | 0.61 |
| Coronary Artery Disease | 2011 | 21405 | 0.99 [0.94;1.05] | 7.38e−01 | 0.71 |
| Atrial Fibrillation | 2554 | 20862 | 1.01 [0.96;1.06] | 7.10e−01 | 0.68 |
| Heart Failure | 1859 | 21557 | 1.03 [0.98;1.09] | 2.35e−01 | 0.59 |
| *SBP and DBP PRS weighted sum* | | | | | |
| Type II Diabetes | 3087 | 20329 | 1.04 [1.00;1.09] | 7.97e−02 | 0.64 |
| Chronic Kidney Disease | 2373 | 21043 | 1.00 [0.95;1.05] | 9.88e−01 | 0.66 |
| Coronary Artery Disease | 2011 | 21405 | 1.00 [0.95;1.05] | 9.68e−01 | 0.68 |
| Atrial Fibrillation | 2554 | 20862 | 1.00 [0.95;1.04] | 8.58e−01 | 0.67 |
| Heart Failure | 1859 | 21557 | 1.04 [0.99;1.10] | 1.36e−01 | 0.60 |
| *HTN PRS (Previously published)* | | | | | |
| Type II Diabetes | 3087 | 20329 | 1.12 [1.08;1.17] | 9.19e−08 | 0.65 |
| Chronic Kidney Disease | 2373 | 21043 | 1.08 [1.03;1.14] | 7.60e−04 | 0.62 |
| Coronary Artery Disease | 2011 | 21405 | 1.01 [0.96;1.06] | 7.72e−01 | 0.69 |
| Atrial Fibrillation | 2554 | 20862 | 0.99 [0.94;1.04] | 6.67e−01 | 0.71 |
| Heart Failure | 1859 | 21557 | 1.06 [1.01;1.12] | 2.06e−02 | 0.64 |

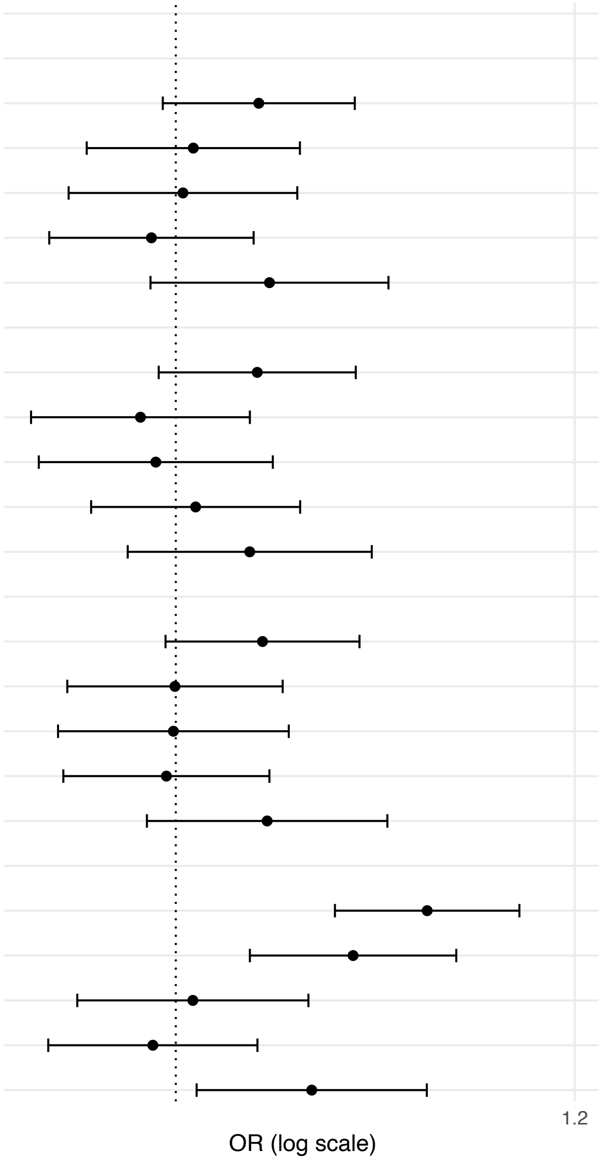


The figure visualizes the estimated associations (odds ratios; ORs; provided numerically and visualized as points in the forest plot) and 95% confidence intervals (CIs; provided numerically and visualized as the intervals in the forest plot) of the best performing SBP and PRSs as selected in the TOPMed dataset (PRS-CSx2), their simple sum, and the previously-developed HTN-PRS with prevalent outcomes in All of Us individuals using antihypertensives. The figure also provides the numbers of cases and controls for each outcome, association p-values computed based on a two-sided 1-degree of freedom Wald test, and AUCs (area under the receiver operating curve). Analyses were adjusted for age, sex, BMI, self-reported race/ethnicity, and the first 10 PCs of genetic data. Abbreviations and definitions. BMI: body mass index; BP: blood pressure; CI: confidence interval; DBP: diastolic blood pressure; PC: principal component; PRS-CSx: continuous shrinkage cross-population PRS method; PRS: polygenic risk score; PVE: percent variance explained; SBP: systolic blood pressure; SD: standard deviation; TOPMed: Trans-Omics for Precision Medicine.

## Supplementary Figure 26: Association of BP and hypertension PRS with prevalent clinical outcomes in the All of Us dataset among individuals not using antihypertensive medication

| | Cases | Controls | OR [95% CI] | P | AUC |
|---|---|---|---|---|---|
| *SBP PRS* | | | | | |
| Type II Diabetes | 1720 | 62774 | 1.06 [1.00;1.12] | 5.21e−02 | 0.64 |
| Chronic Kidney Disease | 1007 | 63487 | 1.09 [1.02;1.17] | 1.68e−02 | 0.69 |
| Coronary Artery Disease | 847 | 63647 | 1.10 [1.02;1.18] | 1.77e−02 | 0.76 |
| Atrial Fibrillation | 1236 | 63258 | 1.01 [0.95;1.08] | 7.73e−01 | 0.78 |
| Heart Failure | 663 | 63831 | 1.10 [1.01;1.20] | 3.79e−02 | 0.63 |
| *DBP PRS* | | | | | |
| Type II Diabetes | 1720 | 62774 | 0.96 [0.91;1.02] | 1.83e−01 | 0.62 |
| Chronic Kidney Disease | 1007 | 63487 | 1.08 [1.01;1.17] | 3.12e−02 | 0.68 |
| Coronary Artery Disease | 847 | 63647 | 1.06 [0.98;1.15] | 1.26e−01 | 0.76 |
| Atrial Fibrillation | 1236 | 63258 | 0.99 [0.93;1.06] | 8.21e−01 | 0.76 |
| Heart Failure | 663 | 63831 | 1.06 [0.96;1.16] | 2.33e−01 | 0.68 |
| *SBP and DBP PRS weighted sum* | | | | | |
| Type II Diabetes | 1720 | 62774 | 1.02 [0.97;1.08] | 4.04e−01 | 0.61 |
| Chronic Kidney Disease | 1007 | 63487 | 1.09 [1.02;1.18] | 1.53e−02 | 0.68 |
| Coronary Artery Disease | 847 | 63647 | 1.09 [1.01;1.18] | 2.93e−02 | 0.76 |
| Atrial Fibrillation | 1236 | 63258 | 1.00 [0.94;1.07] | 9.10e−01 | 0.77 |
| Heart Failure | 663 | 63831 | 1.09 [1.00;1.19] | 6.36e−02 | 0.73 |
| *HTN PRS (Previously published)* | | | | | |
| Type II Diabetes | 1720 | 62774 | 1.05 [1.00;1.11] | 4.55e−02 | 0.64 |
| Chronic Kidney Disease | 1007 | 63487 | 1.02 [0.95;1.09] | 6.18e−01 | 0.66 |
| Coronary Artery Disease | 847 | 63647 | 1.06 [0.98;1.15] | 1.33e−01 | 0.77 |
| Atrial Fibrillation | 1236 | 63258 | 1.01 [0.94;1.07] | 8.64e−01 | 0.77 |
| Heart Failure | 663 | 63831 | 1.13 [1.04;1.23] | 3.54e−03 | 0.70 |

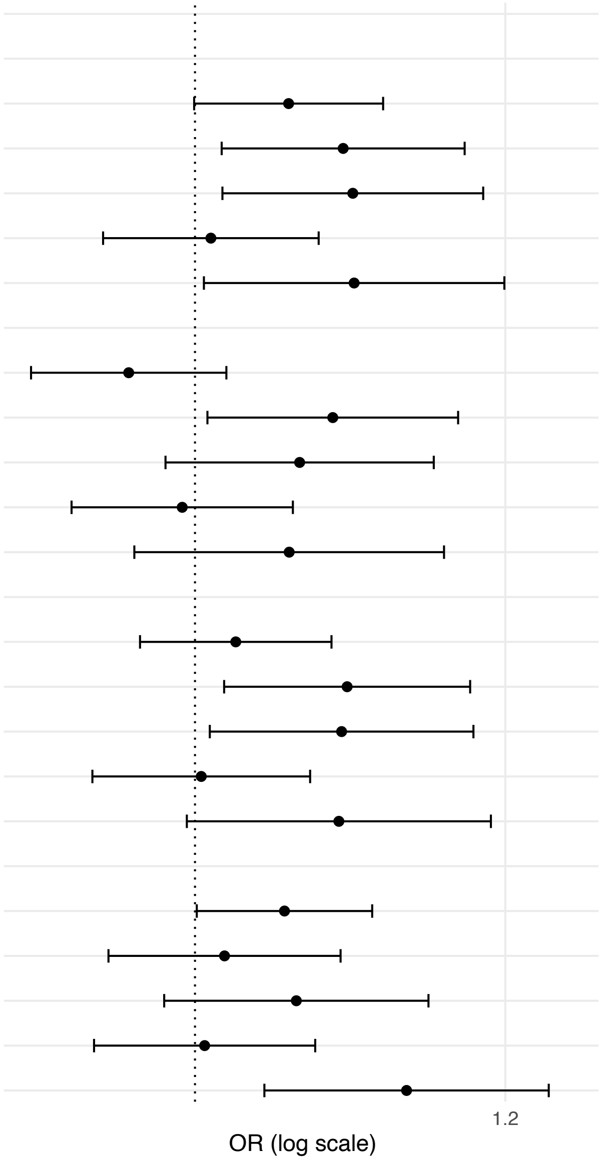


The figure visualizes the estimated associations (odds ratios; ORs; provided numerically and visualized as points in the forest plot) and 95% confidence intervals (CIs; provided numerically and visualized as the intervals in the forest plot) of the best performing SBP and DBP PRSs as selected in the TOPMed dataset (PRS-CSx2), their simple sum, and the previously-developed HTN-PRS with prevalent outcomes in All of Us individuals not using antihypertensives. The figure also provides the numbers of cases and controls for each outcome, association p-values computed based on a two-sided 1-degree of freedom Wald test, and AUCs (area under the receiver operating curve). Analyses were adjusted for age, sex, BMI, self-reported race/ethnicity, and the first 10 PCs of genetic data. Abbreviations and definitions. BMI: body mass index; BP: blood pressure; CI: confidence interval; DBP: diastolic blood pressure; PC: principal component; PRS-CSx: continuous shrinkage cross-population PRS method; PRS: polygenic risk score; PVE: percent variance explained; SBP: systolic blood pressure; SD: standard deviation; TOPMed: Trans-Omics for Precision Medicine.

## Supplementary Note 2: Parent study descriptions

### Amish

**Ethics statement:**

All study protocols were approved by the institutional review board at the University of Maryland Baltimore. Informed consent was obtained from each study participant.

**Amish acknowledgements:**

We gratefully acknowledge our Amish liaisons, research volunteers, field workers, and Amish Research Clinic staff and the extraordinary cooperation and support of the Amish community without which these studies would not have been possible. The Amish studies are supported by grants and contracts from the NIH, including U01 HL072515, U01 HL84756, U01 HL137181, and P30 DK72488. The TOPMed component of the Amish Research Program was supported by NIH grants R01 HL121007, U01 HL072515, and R01 AG18728.

### ARIC

The Atherosclerosis Risk in Communities study (dbGaP accession phs000090) is a population-based prospective cohort study of cardiovascular disease sponsored by the National Heart, Lung, and Blood Institute (NHLBI). ARIC included 15,792 individuals, predominantly European American and African American, aged 45-64 years at baseline (1987-89), chosen by probability sampling from four US communities. Cohort members completed three additional triennial follow-up examinations, a fifth exam in 2011-2013, a sixth exam in 2016-2017, and a seventh exam in 2018-2019. The ARIC study has been described in detail previously [10,11].

**Ethics statement:**

The ARIC study has been approved by Institutional Review Boards (IRB) at all participating institutions: University of North Carolina at Chapel Hill IRB, Johns Hopkins University IRB,

University of Minnesota IRB, and University of Mississippi Medical Center IRB. Study participants provided written informed consent at all study visits.

**ARIC acknowledgements:**
The Atherosclerosis Risk in Communities study has been funded in whole or in part with Federal funds from the National Heart, Lung, and Blood Institute, National Institutes of Health, Department of Health and Human Services (contract numbers HHSN268201700001I, HHSN268201700002I, HHSN268201700003I, HHSN268201700004I and HHSN268201700005I). The authors thank the staff and participants of the ARIC study for their important contributions.

### BioMe

The BioMe Biobank is an ongoing, prospective, hospital- and outpatient- based population research program operated by The Charles Bronfman Institute for Personalized Medicine (IPM) at Mount Sinai. BioMe has enrolled over 50,000 participants between September 2007 and July 2019. BioMe is an Electronic Medical Record (EMR)-linked biobank that integrates research data and clinical care information for consented patients at The Mount Sinai Medical Center, which serves diverse local communities of upper Manhattan with broad health disparities. IPM BioMe populations include 25% of African American ancestry (AA), 36% of Hispanic Latino ancestry (HL), 30% of white European ancestry (EA), and 9% of other ancestry. The BioMe disease burden is reflective of health disparities in the local communities. BioMe operations are fully integrated in clinical care processes, including direct recruitment from clinical sites waiting areas and phlebotomy stations by dedicated BioMe recruiters independent of clinical care providers, prior to or following a clinician standard of care visit. Recruitment currently occurs at a broad spectrum of over 30 clinical care sites.

**Ethics statement:**

The BioMe cohort was approved by the Institutional Review Board at the Icahn School of Medicine at Mount Sinai. All BioMe participants provided written, informed consent for genomic data sharing.

**BioMe acknowledgements:**

The Mount Sinai BioMe Biobank has been supported by The Andrea and Charles Bronfman Philanthropies and in part by Federal funds from the NHLBI and NHGRI (U01HG00638001; U01HG007417; X01HL134588). We thank all participants in the Mount Sinai Biobank. We also thank all our recruiters who have assisted and continue to assist in data collection and management and are grateful for the computational resources and staff expertise provided by Scientific Computing at the Icahn School of Medicine at Mount Sinai.

## CARDIA

The Coronary Artery Risk Development in Young Adults study (dbGaP accession phs000285) is a prospective multicenter study with 5,115 adults Caucasian and African American participants of the age group 18-30 years at baseline, recruited from four centers at the baseline examination in 1985-1986 [12]. The recruitment was done from the total community in Birmingham, AL, from selected census tracts in Chicago, IL and Minneapolis, MN; and from the Kaiser Permanente health plan membership in Oakland, CA. Nine examinations have been completed in the years 0, 2, 5, 7, 10, 15, 20, 25 and 30, with high retention rates (91%, 86%, 81%, 79%, 74%, 72%, 72%, and 71%, respectively) and written informed consent was obtained in each visit.

**Ethics statement:**

All CARDIA participants provided informed consent, and the study was approved by the Institutional Review Boards of the University of Alabama at Birmingham and the University of Texas Health Science Center at Houston.

**CARDIA acknowledgements:**

The Coronary Artery Risk Development in Young Adults Study (CARDIA) is conducted and supported by the National Heart, Lung, and Blood Institute (NHLBI) in collaboration with the University of Alabama at Birmingham (HHSN268201800005I & HHSN268201800007I), Northwestern University (HHSN268201800003I), University of Minnesota (HHSN268201800006I), and Kaiser Foundation Research Institute (HHSN268201800004I). CARDIA was also partially supported by the Intramural Research Program of the National Institute on Aging (NIA) and an intra-agency agreement between NIA and NHLBI (AG0005).

## CFS

The Cleveland Family Study (CFS) was designed to examine the genetic basis of sleep apnea in 2,534 African-American and European-American individuals from 356 families. Index probands with confirmed sleep apnea were recruited from sleep centers in northern Ohio, supplemented with additional family members and neighborhood control families [13]. Four visits occurred between 1990 and 2006; in the first 3, data were collected in participants' homes while the last occurred in a clinical research center (2000 - 2006). Measurements included sleep apnea monitoring, blood pressure, anthropometry, spirometry and other related phenotypes. Blood samples (overnight fasting, before bed and following an oral glucose tolerance test), nasal and oral ultrasound, and ECG were also obtained during the 4th exam. Institutional Review Board approval and signed informed consent was obtained for all participants.

**Ethics statement:**

Cleveland Family Study was approved by the Institutional Review Board (IRB) of Case Western Reserve University and Mass General Brigham (formerly Partners HealthCare). Written informed consent was obtained from all participants.

## CHS

The Cardiovascular Health Study (CHS) is a population-based cohort study initiated by the National Heart, Lung and Blood Institute (NHLBI) in 1987 to determine the risk factors for development and progression of cardiovascular disease (CVD) in older adults, with an emphasis on subclinical measures. The study recruited 5,888 adults aged 65 or older at entry in four U.S. communities and conducted extensive annual clinical exams between 1989-1999 along with semi-annual phone calls, events adjudication, and subsequent data analyses and publications. Additional data are collected by studies ancillary to CHS. In June 1990, four Field Centers (Sacramento, CA; Hagerstown, MD; Winston-Salem, NC; Pittsburgh, PA) completed the recruitment of 5201 participants. Between November 1992 and June 1993, an additional 687 adults of primarily African Americans ethnicity were recruited using similar methods . Blood samples were drawn from all participants at their baseline examination and during follow-up clinic visits and DNA was subsequently extracted from available samples. CHS analyses were limited to participants with available DNA who consented to genetic studies. The baseline examinations consisted of a home interview and a clinic examination that assessed not only traditional risk factors but also measures of subclinical disease, including carotid ultrasound, echocardiography, electrocardiography, and pulmonary function. Between enrollment and 1998-99, participants were seen in the clinic annually, and contacted by phone at 6-month intervals to collect information about hospitalizations and potential cardiovascular events. Major exam components were repeated during annual follow-up examinations through 1999. Cranial MRI scans, retinal photography, and tests of endothelial function were added as new components. Standard protocols for the identification and adjudication of events were implemented during follow-up. The adjudicated events are CHD, angina, heart failure (HF), stroke, transient ischemic attack (TIA), claudication and mortality. Adjudication of cause of death continues using a streamlined protocol; adjudication of other events ended in June 2015. Deep venous thrombosis and pulmonary embolism events from baseline through 2001 were adjudicated in an ancillary study: the Longitudinal Investigation of Thromboembolism Etiology (LITE). Since 1999, participants have been contacted every 6 months by phone, primarily to ascertain health status and for events follow-up. The study was initially approved by

institutional review boards at the Field Centers (Wake Forest, University of California – Davis, Johns Hopkins University, University of Pittsburgh), the Core Laboratory (University of Vermont) and at the Coordinating Center (University of Washington). The University of Washington now handles CHS Data Repository approvals.

**Ethics statement:**

All CHS participants provided informed consent, and the study was approved by the Institutional Review Board [or ethics review committee] of University Washington.

**CHS acknowledgements:**

Cardiovascular Health Study: This research was supported by contracts HHSN268201200036C, HHSN268200800007C, HHSN268201800001C, N01HC55222, N01HC85079, N01HC85080, N01HC85081, N01HC85082, N01HC85083, N01HC85086, 75N92021D00006, and grants U01HL080295, U01HL130114, and HL105756 from the National Heart, Lung, and Blood Institute (NHLBI), with additional contribution from the National Institute of Neurological Disorders and Stroke (NINDS). Additional support was provided by R01AG023629 from the National Institute on Aging (NIA). A full list of principal CHS investigators and institutions can be found at CHS-NHLBI.org. The content is solely the responsibility of the authors and does not necessarily represent the official views of the National Institutes of Health.

## COPDGene

COPDGene [14] is a cohort study for respiratory disease research, recruiting more than 10,000 subjects between the ages of 45 and 80 who had at least 10 pack-years of smoking during January 2008 - June 2011 at 21 clinical centers. Participants were characterized using spirometry, six-minute walk, inspiratory and expiratory chest CT scans, respiratory symptoms, medical history, medication history and 36-Item short form health survey. In the current analysis, we only used COPDGene control participants (meaning, individuals without COPD).

**Ethics statement:**

All COPDGene participants provided written informed consent, and the study was approved by the Institutional Review Boards of the participating clinical centers.

**COPDGene acknowledgements:**

The COPDGene project described was supported by Award Number U01 HL089897 and Award Number U01 HL089856 from the National Heart, Lung, and Blood Institute. The content is solely the responsibility of the authors and does not necessarily represent the official views of the National Heart, Lung, and Blood Institute or the National Institutes of Health. The COPDGene project is also supported by the COPD Foundation through contributions made to an Industry Advisory Board comprised of AstraZeneca, Boehringer Ingelheim, GlaxoSmithKline, Novartis, Pfizer, Siemens and Sunovion. A full listing of COPDGene investigators can be found at: http://www.copdgene.org/directory

## FHS

The Framingham Heart Study (dbGaP accession phs000007) began in 1948 with the recruitment of an original cohort of 5,209 men and women (mean age 44 years; 55 percent women). In 1971 a second generation of study participants was enrolled; this cohort (mean age 37 years; 52% women) consisted of 5,124 children and spouses of children of the original cohort. A third-generation cohort of 4,095 children of offspring cohort participants (mean age 40 years; 53 percent women) was enrolled in 2002-2005 and are seen every 4 to 8 years. Details of study designs for the three cohorts are summarized elsewhere [15–17]. At each clinic visit, a medical history was obtained, and participants underwent a physical examination. Only study participants consented for genetic and non-genetic data are included. FHS has been approved by the Boston University IRB

**Ethics statement:**

The Framingham Heart Study was approved by the Institutional Review Board of the Boston University Medical Center. All study participants provided written informed consent.

**FHS acknowledgements:**

The Framingham Heart Study (FHS) acknowledges the support of contracts NO1-HC-25195, HHSN268201500001I and 75N92019D00031 from the National Heart, Lung and Blood Institute and grant supplement R01 HL092577-06S1 for this research. We also acknowledge the dedication of the FHS study participants without whom this research would not be possible. Dr. Vasan is supported in part by the Evans Medical Foundation and the Jay and Louis Coffman Endowment from the Department of Medicine, Boston University School of Medicine.

## GENOA

The Genetic Epidemiology Network of Arteriopathy (GENOA) study (dbGaP accession phs000379), a part of the Family Blood Pressure Program (FBPP Investigators, 2002), consists of hypertensive sibships that were recruited for linkage and association studies in order to identify genes that influence blood pressure and its target organ damage (Daniels, 2004). In the initial phase of the GENOA study (Phase I: 1996-2001), all members of sibships containing ≥ 2 individuals with essential hypertension clinically diagnosed before age 60 were invited to participate, including both hypertensive and normotensive siblings. In the second phase of the GENOA study (Phase II: 2000-2004), 1,239 non-Hispanic white and 1,482 African American participants were successfully re-recruited to measure potential target organ damage due to hypertension.

**Ethics statement:**

Written informed consent was obtained from all subjects and approval was granted by participating institutional review boards (University of Michigan, University of Mississippi Medical Center, and Mayo Clinic).

**GENOA acknowledgements:**

Support for the Genetic Epidemiology Network of Arteriopathy (GENOA) was provided by the National Heart, Lung and Blood Institute (U01 HL054457, U01 HL054464, U01 HL054481, R01 HL119443, and R01 HL087660) of the National Institutes of Health. DNA extraction for “NHLBI

TOPMed: Genetic Epidemiology Network of Arteriopathy" (phs001345) was performed at the Mayo Clinic Genotyping Core, and WGS was performed at the DNA Sequencing and Gene Analysis Center at the University of Washington (3R01HL055673-18S1) and the Broad Institute (HHSN268201500014C). We would like to thank the GENOA participants.

## GenSalt

The GenSalt study (dbGaP accession phs000784) is a unique NHLBI- sponsored family feeding-study designed to examine the interaction between genes and dietary sodium and potassium intake on BP. A detailed description of the GenSalt study design and participants has been reported previously [18]. Briefly, 3,142 participants from 633 Han families from rural, north China were ascertained through a proband with untreated pre-hypertension or stage-1 hypertension identified from a population-based BP screening. A total of 1,906 GenSalt probands and their siblings, spouses, and offspring were eligible. Among them, 1,818 took part in the TOPMed WGS program and had BP and covariable data available for the current analysis. Three morning BP measurements were obtained according to a standard protocol during each of the 3-days of baseline observation. All BP readings were measured by trained and certified observers using a random–zero sphygmomanometer using a standard protocol [19]. BP was measured with the participant in the sitting position after 5 minutes of rest. In addition, participants were advised to avoid alcohol, cigarette smoking, coffee/tea, and exercise for at least 30 minutes prior to their BP measurements. Systolic and diastolic BP measures were taken in triplicate during each day of the three-day baseline observation. After throwing out the first measure, the subsequent two measures obtained on the first day of baseline observation were averaged and used in this analysis.

**Ethics statement:**

All subjects provided informed consent and the GenSalt study was approved by the Institutional Review Board (IRB) of all participating institutes in the US and China.

**GenSalt acknowledgements:**

The Genetic Epidemiology Network of Salt-Sensitivity (GenSalt) was supported by research grants (U01HL072507, R01HL087263, and R01HL090682) from the National Heart, Lung and Blood Institute, National Institutes of Health, Bethesda, MD.

## HCHS/SOL

The Hispanic Community Health Study/Study of Latinos (dbGaP accession phs000810) is a community-based longitudinal cohort study of 16,415 self-identified Hispanic/Latino persons aged 18–74 years and selected from households in predefined census-block groups across four US field centers (in Chicago, Miami, the Bronx, and San Diego). The census-block groups were chosen to provide diversity among cohort participants with regard to socioeconomic status and national origin or background [20,21]. The HCHS/SOL cohort includes participants who self-identified as having a Hispanic/Latino background; the largest groups are Central American (n = 1,730), Cuban (n = 2,348), Dominican (n = 1,460), Mexican (n = 6,471), Puerto Rican (n = 2,728), and South American (n = 1,068). The HCHS/SOL baseline clinical examination occurred between 2008 and 2011 and included comprehensive biological, behavioral, and sociodemographic assessments. Visit 2 took place between 2014 and 2017, which re-examined 11,623 participants from the baseline sample. Visit 3 has started in 2020 and will last 3 years. In addition to clinic visit, participants are contacted annually to assess clinical outcomes. The study was approved by the Institutional Review Boards at each participating institution and written informed consent was obtained from all participants.

**Ethics statement:**

This study was approved by the institutional review boards (IRBs) at each field center, where all participants gave written informed consent, and by the Non-Biomedical IRB at the University of North Carolina at Chapel Hill, to the HCHS/SOL Data Coordinating Center. All IRBs approving the study are: Non-Biomedical IRB at the University of North Carolina at Chapel Hill. Chapel Hill, NC; Einstein IRB at the Albert Einstein College of Medicine of Yeshiva University. Bronx, NY; IRB at Office for the Protection of Research Subjects (OPRS), University of Illinois at Chicago. Chicago,

IL; Human Subject Research Office, University of Miami. Miami, FL; Institutional Review Board of San Diego State University. San Diego, CA.

**HCHS/SOL acknowledgements:**

The Hispanic Community Health Study/Study of Latinos is a collaborative study supported by contracts from the National Heart, Lung, and Blood Institute (NHLBI) to the University of North Carolina (HHSN268201300001I / N01-HC-65233), University of Miami (HHSN268201300004I / N01-HC- 65234), Albert Einstein College of Medicine (HHSN268201300002I / N01-HC-65235), University of Illinois at Chicago – HHSN268201300003I / N01- HC-65236 Northwestern Univ), and San Diego State University (HHSN268201300005I / N01-HC-65237). The following Institutes/Centers/Offices have contributed to the HCHS/SOL through a transfer of funds to the NHLBI: National Institute on Minority Health and Health Disparities, National Institute on Deafness and Other Communication Disorders, National Institute of Dental and Craniofacial Research, National Institute of Diabetes and Digestive and Kidney Diseases, National Institute of Neurological Disorders and Stroke, NIH Institution-Office of Dietary Supplements.

## JHS

The Jackson Heart Study (dbGaP accession phs000286) is a longitudinal investigation of genetic and environmental risk factors associated with the disproportionate burden of cardiovascular disease in African Americans [22,23]. JHS is funded by the NHLBI and the National Institute on Minority Health and Health Disparities (NIMHD). JHS is an expansion of the ARIC study in its Jackson Field Center. At baseline, the JHS recruited 5306 African American residents of the Jackson Mississippi Metropolitan Statistical Area aged, approximately 6.6% of all African American adults aged 35-84 residing in the area. Participants were recruited via random sampling (17% of participants), volunteers (30%), prior participants in the Atherosclerosis Risk in Communities (ARIC) study (31%), and secondary family members (22%). Among these participants, approximately 3400 gave consent that allows genetic research. JHS participants received three back-to-back clinical examinations (Exam 1, 2000-2004; Exam 2, 2005-2008; and Exam 3, 2009-2013), and a fourth clinical examination has started in 2020. Participants are also

contacted annually by telephone to update personal and health information including vital status, interim medical events, hospitalizations, functional status and sociocultural information

**Ethics statement:**

The JHS study was approved by Jackson State University, Tougaloo College, and the University of Mississippi Medical Center IRBs, and all participants provided written informed consent.

**JHS acknowledgements:**

The Jackson Heart Study (JHS) is supported and conducted in collaboration with Jackson State University (HHSN268201800013I), Tougaloo College (HHSN268201800014I), the Mississippi State Department of Health (HHSN268201800015I) and the University of Mississippi Medical Center (HHSN268201800010I, HHSN268201800011I and HHSN268201800012I) contracts from the National Heart, Lung, and Blood Institute (NHLBI) and the National Institute for Minority Health and Health Disparities (NIMHD). The authors also wish to thank the staffs and participants of the JHS.

## WHI

The Women's Health Initiative (WHI) cohort. The WHI is a prospective national health study focused on identifying optimal strategies for preventing chronic diseases that are the major causes of death and disability in postmenopausal women [refs]. The WHI initially recruited 161,808 women between 1993 and 1997 with the goal of including a socio-demographically diverse population with diversity background groups proportionate to the total minority population of US women aged 50-79 years. The WHI consists of two major parts: a set of randomized Clinical Trials and an Observational Study. The WHI Clinical Trials (CT; N=68,132) includes three overlapping components, each a randomized controlled comparison: the Hormone Therapy Trials (HT), Dietary Modification Trial, and Calcium and Vitamin D Trial. A parallel prospective observational study (OS; N = 93,676) examined biomarkers and risk factors associated with various chronic diseases. While the HT trials ended in the mid-2000s, active follow-up of the WHI-CT and WHI-OS cohorts has continued for over 25 years, with the

accumulation of large numbers of diverse clinical outcomes, risk factor measurements, medication use, and many other types of data.

**Ethics statement:**

All WHI participants provided informed consent and the study was approved by the Institutional Review Board (IRB) of the Fred Hutchinson Cancer Research Center.

**WHI acknowledgements:**

The WHI program is funded by the National Heart, Lung, and Blood Institute, National Institutes of Health, U.S. Department of Health and Human Services through contracts 75N92021D00001, 75N92021D00002, 75N92021D00003, 75N92021D00004, 75N92021D00005.

## MESA

The Multi-Ethnic Study of Atherosclerosis (dbGaP accession phs000209) is a study of the characteristics of subclinical cardiovascular disease (disease detected non-invasively before it has produced clinical signs and symptoms) and the risk factors that predict progression to clinically overt cardiovascular disease or progression of the subclinical disease [24]. MESA consisted of a diverse, population-based sample of an initial 6,814 asymptomatic men and women aged 45-84. 38 percent of the recruited participants were white, 28 percent African American, 22 percent Hispanic, and 12 percent Asian, predominantly of Chinese descent. Participants were recruited from six field centers across the United States: Wake Forest University, Columbia University, Johns Hopkins University, University of Minnesota, Northwestern University and University of California - Los Angeles. Participants are being followed for identification and characterization of cardiovascular disease events, including acute myocardial infarction and other forms of coronary heart disease (CHD), stroke, and congestive heart failure; for cardiovascular disease interventions; and for mortality. The first examination took place over two years, from July 2000 - July 2002. It was followed by five examination periods that were 17-20 months in length. Participants have been contacted every 9 to 12 months throughout the study to assess clinical morbidity and mortality.

**Ethics statements:**

All MESA participants provided written informed consent, and the study was approved by the Institutional Review Boards at The Lundquist Institute (formerly Los Angeles BioMedical Research Institute) at Harbor-UCLA Medical Center, University of Washington, Wake Forest School of Medicine, Northwestern University, University of Minnesota, Columbia University, and Johns Hopkins University.

**MESA acknowledgements:**

MESA and the MESA SHARe projects are conducted and supported by the National Heart, Lung, and Blood Institute (NHLBI) in collaboration with MESA investigators. Support for MESA is provided by contracts 75N92020D00001, HHSN268201500003I, N01-HC-95159, 75N92020D00005, N01-HC-95160, 75N92020D00002, N01-HC-95161, 75N92020D00003, N01-HC-95162, 75N92020D00006, N01-HC-95163, 75N92020D00004, N01-HC-95164, 75N92020D00007, N01-HC-95165, N01-HC-95166, N01-HC-95167, N01-HC-95168, N01-HC-95169, UL1-TR-000040, UL1-TR-001079, and UL1-TR-001420, UL1TR001881, DK063491, and R01HL105756. Funding for SHARe genotyping was provided by NHLBI Contract N02-HL-64278. Genotyping was performed at Affymetrix (Santa Clara, California, USA) and the Broad Institute of Harvard and MIT (Boston, Massachusetts, USA) using the Affymetrix Genome-Wide Human SNP Array 6.0. The authors thank the other investigators, the staff, and the participants of the MESA study for their valuable contributions. A full list of participating MESA investigators and institutes can be found at http://www.mesa-nhlbi.org.

## THRV

The THRV-TOPMed study comprises 2,353 Taiwan Chinese participants in three cohorts: The SAPPHIRe (Stanford-Asian Pacific Program in Hypertension and Insulin Resistance) Family cohort of approximately 300 hypertensive sibships (N=1,271) and two hospital-based cohorts, the TSGH (Tri-Service General Hospital) cohort (N=160) and the TCVGH (Taichung Veterans General Hospital) cohort (N=922) that provide population-based controls (unrelated

hypertensive or non-hypertensive) matched to SAPPHIRe samples. All three cohorts are based in Taiwan. The 1,271 SAPPHIRe subjects were previously recruited as part of the SAPPHIRe Network of the NHLBI-sponsored Family Blood Pressure Program (FBPP). The SAPPHIRe families were recruited to have two or more hypertensive sibs, with some families having one normotensive/hypotensive sib. The two Hospital-based cohorts (TSGH and TCVGH) both recruited unrelated subjects at the SAPPHIRe field centers/hospitals in Taiwan, that matched with the SAPPHIRe subjects for age, sex, and BMI category. Several metabolic variables associated with blood pressure and insulin resistance were measured in the first 5-year SAPPHIRe funding from the NHLBI (1995-2000). Additional phenotyping through return visits and regular follow ups occurred between 2001 and 2008 which included echocardiographic and multi-detector row CT imaging procedures.

**Ethics statements:**

All THRV participants provided informed consent, and the study was approved by the Institutional Review Board at The Lundquist Institute (formerly Los Angeles BioMedical Research Institute, or LA BioMed) at Harbor-UCLA Medical Center, and at Washington University in St. Louis.

**THRV acknowledgments:**

The Rare Variants for Hypertension in Taiwan Chinese (THRV) is supported by the National Heart, Lung, and Blood Institute (NHLBI) grant (R01HL111249) and its participation in TOPMed is supported by an NHLBI supplement (R01HL111249-04S1). THRV is a collaborative study between Washington University in St. Louis, LA BioMed at Harbor UCLA, University of Texas in Houston, Taichung Veterans General Hospital, Taipei Veterans General Hospital, Tri-Service General Hospital, National Health Research Institutes, National Taiwan University, and Baylor University. THRV is based (substantially) on the parent SAPPHIRe study, along with additional population-based and hospital-based cohorts. SAPPHIRe was supported by NHLBI grants (U01HL54527, U01HL54498) and Taiwan funds, and the other cohorts were supported by Taiwan funds.

## Supplementary Note 3: TOPMed and CCDG acknowledgements

Molecular data for the Trans-Omics in Precision Medicine (TOPMed) program was supported by the National Heart, Lung and Blood Institute (NHLBI). Genome sequencing for “NHLBI TOPMed: Genetics of Cardiometabolic Health in the Old Order Amish Study” (phs000956) were performed at the Broad Institute of MIT and Harvard (HHSN268201500014C).

Genome sequencing for “NHLBI TOPMed: Whole Genome Sequencing and Related Phenotypes in the Framingham Heart Study” (phs000974.v4.p3) was performed at the Broad Institute Genomics Platform (3R01HL092577-06S1, 3U54HG003067-12S2). Genome sequencing for the “NHLBI TOPMed: Genetic Epidemiology Network of Arteriopathy (GENOA)” (phs001345.v2.p1) was performed at the Broad Institute Genomics Platform (HHSN268201500014C) and the Northwest Genomics Center (3R01HL055673-18S1). Genome sequencing for “NHLBI TOPMed: The Jackson Heart Study” (phs000964.v1.p1) was performed at the Northwest Genomics Center (HHSN268201100037C). Genome sequencing for the “NHLBI TOPMed: The Atherosclerosis Risk in Communities Study” (phs001211.v3.p2) was performed at the Broad Institute Genomics Platform (3R01HL092577-06S1) and the Baylor College of Medicine Human Genome Sequencing Center (HHSN268201500015C, 3U54HG003273-12S2). Genome sequencing for “NHLBI TOPMed: Coronary Artery Risk Development in Young Adults Study” (phs001612.v1.p1) was performed at the Baylor College of Medicine Human Genome Sequencing Center (HHSN268201600033I). Genome sequencing for “NHLBI TOPMed: Cleveland Family Study” (phs000954.v3.p2) was performed at the Northwest Genomics Center (3R01HL098433-05S1, HHSN268201600032I). Genome sequencing for “NHLBI TOPMed: Genetic Epidemiology of COPD (COPDGene) in the TOPMed Program” (phs000951) was performed at the University of

Washington Northwest Genomics Center (3R01 HL089856-08S1) and the Broad Institute of MIT and Harvard (HHSN268201500014C). Genomics sequencing for “NHLBI TOPMed: Cardiovascular Health Study” (phs001368.v2.p1) was performed at the Baylor College of Medicine Human Genome Sequencing Center (3U54HG003273-12S2, HHSN268201500015C, HHSN268201600033I). Genome sequencing for “NHLBI TOPMed: Hispanic Community Health Study/Study of Latinos” (phs001395.v1.p1) was performed at the Baylor College of Medicine Human Genome Sequencing Center (HHSN268201600033I). Genome sequencing for “NHLBI TOPMed: Women’s Health Initiative (WHI)” (phs001237.v2.p1) was performed at the Broad Institute of MIT and Harvard (HHSN268201500014C). Genome sequencing for “NHLBI TOPMed: Multi-Ethnic Study of Atherosclerosis” (phs001416.v2.p1) was performed at Broad Institute Genomics Platform (HHSN268201500014C, 3U54HG003067-13S1). Genome sequencing for “NHLBI TOPMed: Genetic Epidemiology Network of Salt Sensitivity (GenSalt)” (phs001217.v3.p1) was performed at the Baylor College of Medicine Human Genome Sequencing Center (HHSN268201500015C). Genome sequencing for “NHLBI TOPMed: Rare Variants for Hypertension in Taiwan Chinese (THRV)” (phs001387.v3.p1) was performed at the Baylor College of Medicine Human Genome Sequencing Center (3R01HL111249-04S1, HHSN26820150015C). Core support including centralized genomic read mapping and genotype calling, along with variant quality metrics and filtering were provided by the TOPMed Informatics Research Center (3R01HL-117626-02S1; contract HHSN268201800002I). Core support including phenotype harmonization, data management, sample-identity QC, and general program coordination were provided by the TOPMed Data Coordinating Center (R01HL-120393; U01HL-120393; contract HHSN268201800001I). We gratefully acknowledge the studies

and participants who provided biological samples and data for TOPMed. The Genome Sequencing Program (GSP) was funded by the National Human Genome Research Institute (NHGRI), the National Heart, Lung, and Blood Institute (NHLBI), and the National Eye Institute (NEI). The GSP Coordinating Center (U24 HG008956) contributed to cross-program scientific initiatives and provided logistical and general study coordination. The Centers for Common Disease Genomics (CCDG) program was supported by NHGRI and NHLBI, and whole genome sequencing was performed at the Baylor College of Medicine Human Genome Sequencing Center (UM1 HG008898 and R01HL059367).

## Supplementary Note 4: TOPMed consortium members

Namiko Abe[1], Goncalo Abecasis[2], Francois Aguet[3], Christine Albert[4], Laura Almasy[5], Alvaro Alonso[6], Seth Ament[7], Peter Anderson[8], Pramod Anugu[9], Deborah Applebaum-Bowden[10], Kristin Ardlie[3], Dan Arking[11], Donna K Arnett[12], Allison Ashley-Koch[13], Stella Aslibekyan[14], Tim Assimes[15], Paul Auer[16], Dimitrios Avramopoulos[11], Najib Ayas[17], Adithya Balasubramanian[18], John Barnard[19], Kathleen Barnes[20], R. Graham Barr[21], Emily Barron-Casella[11], Lucas Barwick[22], Terri Beaty[11], Gerald Beck[23], Diane Becker[24], Lewis Becker[11], Rebecca Beer[25], Amber Beitelshees[7], Emelia Benjamin[26], Takis Benos[27], Marcos Bezerra[28], Larry Bielak[2], Joshua Bis[29], Thomas Blackwell[2], John Blangero[30], Nathan Blue[31], Eric Boerwinkle[32], Donald W. Bowden[33], Russell Bowler[34], Jennifer Brody[8], Ulrich Broeckel[35], Jai Broome[8], Deborah Brown[36], Karen Bunting[1], Esteban Burchard[37], Carlos Bustamante[38], Erin Buth[39], Brian Cade[40], Jonathan Cardwell[41], Vincent Carey[42], Julie Carrier[43], April P. Carson[44], Cara Carty[45], Richard Casaburi[46], Juan P Casas Romero[47], James Casella[11], Peter Castaldi[48], Mark Chaffin[3], Christy Chang[7], Yi-Cheng Chang[49], Daniel Chasman[50], Sameer Chavan[41], Bo-Juen Chen[1], Wei-Min Chen[51], Yii-Der Ida Chen[52], Michael Cho[42], Seung Hoan Choi[3], Lee-Ming Chuang[53], Mina Chung[54], Ren-Hua Chung[55], Clary Clish[56], Suzy Comhair[57], Matthew Conomos[39], Elaine Cornell[58], Adolfo Correa[59], Carolyn Crandall[46], James Crapo[60], L. Adrienne Cupples[61], Joanne Curran[62], Jeffrey Curtis[63], Brian Custer[64], Coleen Damcott[7], Dawood Darbar[65], Sean David[66], Colleen Davis[8], Michelle Daya[41], Mariza de Andrade[67], Lisa de las Fuentes[68], Paul de Vries[69], Michael DeBaun[70], Ranjan Deka[71], Dawn DeMeo[42], Scott Devine[7], Huyen Dinh[18], Harsha Doddapaneni[18], Qing Duan[72], Shannon Dugan-Perez[73], Ravi Duggirala[74], Jon Peter Durda[75], Susan K. Dutcher[76], Charles Eaton[77], Lynette Ekunwe[9], Adel El Boueiz[78], Patrick Ellinor[79], Leslie Emery[8], Serpil Erzurum[80], Charles Farber[51], Jesse Farek[18], Tasha Fingerlin[81], Matthew Flickinger[2], Myriam Fornage[82], Nora Franceschini[83], Chris Frazar[8], Mao Fu[7], Stephanie M. Fullerton[8], Lucinda Fulton[84], Stacey Gabriel[3], Weiniu Gan[25], Shanshan Gao[41], Yan Gao[9], Margery Gass[85], Heather Geiger[86], Bruce Gelb[87], Mark Geraci[88], Soren Germer[1], Robert Gerszten[89], Auyon Ghosh[42], Richard Gibbs[18], Chris Gignoux[15], Mark Gladwin[90], David Glahn[91],

Stephanie Gogarten[8], Da-Wei Gong[7], Harald Goring[92], Sharon Graw[93], Kathryn J. Gray[94], Daniel Grine[41], Colin Gross[2], C. Charles Gu[84], Yue Guan[7], Xiuqing Guo[52], Namrata Gupta[95], Jeff Haessler[85], Michael Hall[96], Yi Han[18], Patrick Hanly[97], Daniel Harris[98], Nicola L. Hawley[99], Jiang He[100], Ben Heavner[39], Susan Heckbert[101], Ryan Hernandez[37], David Herrington[102], Craig Hersh[103], Bertha Hidalgo[14], James Hixson[32], Brian Hobbs[104], John Hokanson[41], Elliott Hong[7], Karin Hoth[105], Chao (Agnes) Hsiung[106], Jianhong Hu[18], Yi-Jen Hung[107], Haley Huston[108], Chii Min Hwu[109], Marguerite Ryan Irvin[14], Rebecca Jackson[110], Deepti Jain[8], Cashell Jaquish[111], Jill Johnsen[112], Andrew Johnson[25], Craig Johnson[8], Rich Johnston[6], Kimberly Jones[11], Hyun Min Kang[113], Robert Kaplan[114], Sharon Kardia[2], Shannon Kelly[115], Eimear Kenny[87], Michael Kessler[7], Alyna Khan[8], Ziad Khan[18], Wonji Kim[116], John Kimoff[117], Greg Kinney[118], Barbara Konkle[119], Charles Kooperberg[85], Holly Kramer[120], Christoph Lange[121], Ethan Lange[41], Leslie Lange[122], Cathy Laurie[8], Cecelia Laurie[8], Meryl LeBoff[42], Jiwon Lee[42], Sandra Lee[18], Wen-Jane Lee[109], Jonathon LeFaive[2], David Levine[8], Daniel Levy[25], Joshua Lewis[7], Xiaohui Li[52], Yun Li[72], Henry Lin[52], Honghuang Lin[123], Xihong Lin[124], Simin Liu[125], Yongmei Liu[126], Yu Liu[127], Ruth J.F. Loos[128], Steven Lubitz[79], Kathryn Lunetta[129], James Luo[25], Ulysses Magalang[130], Michael Mahaney[62], Barry Make[11], Ani Manichaikul[51], Alisa Manning[131], JoAnn Manson[42], Lisa Martin[132], Melissa Marton[86], Susan Mathai[41], Rasika Mathias[11], Susanne May[39], Patrick McArdle[7], Merry-Lynn McDonald[133], Sean McFarland[116], Stephen McGarvey[134], Daniel McGoldrick[135], Caitlin McHugh[39], Becky McNeil[136], Hao Mei[9], James Meigs[137], Vipin Menon[18], Luisa Mestroni[93], Ginger Metcalf[18], Deborah A Meyers[138], Emmanuel Mignot[139], Julie Mikulla[25], Nancy Min[9], Mollie Minear[140], Ryan L Minster[90], Braxton D. Mitchell[7], Matt Moll[48], Zeineen Momin[18], May E. Montasser[7], Courtney Montgomery[141], Donna Muzny[18], Josyf C Mychaleckyj[51], Girish Nadkarni[87], Rakhi Naik[11], Take Naseri[142], Pradeep Natarajan[3], Sergei Nekhai[143], Sarah C. Nelson[39], Bonnie Neltner[41], Caitlin Nessner[18], Deborah Nickerson[144], Osuji Nkechinyere[18], Kari North[72], Jeff O'Connell[145], Tim O'Connor[7], Heather Ochs-Balcom[146], Geoffrey Okwuonu[18], Allan Pack[147], David T. Paik[148], Nicholette Palmer[149], James Pankow[150], George Papanicolaou[25], Cora Parker[151], Gina Peloso[152], Juan Manuel Peralta[74], Marco Perez[15], James Perry[7], Ulrike Peters[153], Patricia Peyser[2], Lawrence S Phillips[6], Jacob Pleiness[2], Toni Pollin[7], Wendy Post[154], Julia Powers Becker[155], Meher Preethi Boorgula[41], Michael Preuss[87], Bruce Psaty[8], Pankaj Qasba[25], Dandi Qiao[42], Zhaohui Qin[6], Nicholas Rafaels[156], Laura Raffield[157], Mahitha Rajendran[18], Vasan S. Ramachandran[129], D.C. Rao[84], Laura Rasmussen-Torvik[158], Aakrosh Ratan[51], Susan Redline[48], Robert Reed[7], Catherine Reeves[159], Elizabeth Regan[60], Alex Reiner[160], Muagututiâ€˜a Sefuiva Reupena[161], Ken Rice[8], Stephen Rich[51], Rebecca Robillard[162], Nicolas Robine[86], Dan Roden[163], Carolina Roselli[3], Jerome Rotter[164], Ingo Ruczinski[11], Alexi Runnels[86], Pamela Russell[41], Sarah Ruuska[165], Kathleen Ryan[7], Ester Cerdeira Sabino[166], Danish Saleheen[167], Shabnam Salimi[168], Sejal Salvi[18], Steven Salzberg[11], Kevin Sandow[169], Vijay G. Sankaran[170], Jireh Santibanez[18], Karen Schwander[84], David Schwartz[41], Frank Sciurba[90], Christine Seidman[171], Jonathan Seidman[172], FrÃ©dÃ©ric SÃ©riÃ¨s[173], Vivien Sheehan[174], Stephanie L. Sherman[175], Amol Shetty[7], Aniket Shetty[41], Wayne Hui-Heng Sheu[109], M. Benjamin Shoemaker[176], Brian Silver[177], Edwin Silverman[42], Robert Skomro[178], Albert Vernon Smith[179], Jennifer Smith[2], Josh Smith[8], Nicholas Smith[180], Tanja Smith[1], Sylvia Smoller[114], Beverly Snively[181], Michael Snyder[182], Tamar Sofer[89], Nona Sotoodehnia[8], Adrienne M. Stilp[8], Garrett Storm[183], Elizabeth Streeten[7], Jessica Lasky Su[184], Yun Ju Sung[84], Jody Sylvia[42], Adam Szpiro[8], Daniel Taliun[2], Hua Tang[182], Margaret Taub[11], Kent D. Taylor[185], Matthew Taylor[93], Simeon Taylor[7], Marilyn Telen[13], Timothy A. Thornton[8], Machiko Threlkeld[186], Lesley Tinker[187], David Tirschwell[8], Sarah Tishkoff[188], Hemant Tiwari[189],

Catherine Tong[190], Russell Tracy[191], Michael Tsai[150], Dhananjay Vaidya[11], David Van Den Berg[192], Peter VandeHaar[2], Scott Vrieze[150], Tarik Walker[41], Robert Wallace[105], Avram Walts[41], Fei Fei Wang[8], Heming Wang[193], Jiongming Wang[179], Karol Watson[46], Jennifer Watt[18], Daniel E. Weeks[194], Joshua Weinstock[113], Bruce Weir[8], Scott T Weiss[195], Lu-Chen Weng[79], Jennifer Wessel[196], Cristen Willer[63], Kayleen Williams[39], L. Keoki Williams[197], Scott Williams[198], Carla Wilson[42], James Wilson[199], Lara Winterkorn[86], Quenna Wong[8], Baojun Wu[200], Joseph Wu[148], Huichun Xu[7], Lisa Yanek[11], Ivana Yang[41], Ketian Yu[2], Seyedeh Maryam Zekavat[3], Yingze Zhang[201], Snow Xueyan Zhao[60], Wei Zhao[202], Xiaofeng Zhu[203], Elad Ziv[204], Michael Zody[1], Sebastian Zoellner[2]

1 - New York Genome Center, New York, New York, 10013; 2 - University of Michigan, Ann Arbor, Michigan, 48109; 3 - Broad Institute, Cambridge, Massachusetts, 02142; 4 - Cedars Sinai, Boston, Massachusetts, 02114; 5 - Children's Hospital of Philadelphia, University of Pennsylvania, Philadelphia, Pennsylvania, 19104; 6 - Emory University, Atlanta, Georgia, 30322; 7 - University of Maryland, Baltimore, Maryland, 21201; 8 - University of Washington, Seattle, Washington, 98195; 9 - University of Mississippi, Jackson, Mississippi, 38677; 10 - National Institutes of Health, Bethesda, Maryland, 20892; 11 - Johns Hopkins University, Baltimore, Maryland, 21218; 12 - University of South Carolina; 13 - Duke University, Durham, North Carolina, 27708; 14 - University of Alabama, Birmingham, Alabama, 35487; 15 - Stanford University, Stanford, California, 94305; 16 - Medical College of Wisconsin, Milwaukee, Wisconsin, 53211; 17 - Providence Health Care, Medicine, Vancouver; 18 - Baylor College of Medicine Human Genome Sequencing Center, Houston, Texas, 77030; 19 - Cleveland Clinic, Cleveland, Ohio, 44195; 20 - Tempus, University of Colorado Anschutz Medical Campus, Aurora, Colorado, 80045; 21 - Columbia University, New York, New York, 10032; 22 - The Emmes Corporation, LTRC, Rockville, Maryland, 20850; 23 - Cleveland Clinic, Quantitative Health Sciences, Cleveland, Ohio, 44195; 24 - Johns Hopkins University, Medicine, Baltimore, Maryland, 21218; 25 - National Heart, Lung, and Blood Institute, National Institutes of Health, Bethesda, Maryland, 20892; 26 - Boston University, Massachusetts General Hospital, Boston University School of Medicine, Boston, Massachusetts, 02114; 27 - University of Florida, Epidemiology, Gainesville, Florida, 32610; 28 - FundaÃ§Ã£o de Hematologia e Hemoterapia de Pernambuco - Hemope, Recife, 52011-000; 29 - University of Washington, Cardiovascular Health Research Unit, Department of Medicine, Seattle, Washington, 98195; 30 - University of Texas Rio Grande Valley School of Medicine, Human Genetics, Brownsville, Texas, 78520; 31 - University of Utah, Obstetrics and Gynecology, Salt Lake City, Utah, 84132; 32 - University of Texas Health at Houston, Houston, Texas, 77225; 33 - Wake Forest Baptist Health, Department of Biochemistry, Winston-Salem, North Carolina, 27157; 34 - National Jewish Health, National Jewish Health, Denver, Colorado, 80206; 35 - Medical College of Wisconsin, Pediatrics, Milwaukee, Wisconsin, 53226; 36 - University of Texas Health at Houston, Pediatrics, Houston, Texas, 77030; 37 - University of California, San Francisco, San Francisco, California, 94143; 38 - Stanford University, Biomedical Data Science, Stanford, California, 94305; 39 - University of Washington, Biostatistics, Seattle, Washington, 98195; 40 - Brigham & Women's Hospital, Brigham and Women's Hospital, Boston, Massachusetts, 02115; 41 - University of Colorado at Denver, Denver, Colorado, 80204; 42 - Brigham & Women's Hospital, Boston, Massachusetts, 02115; 43 - University of Montreal; 44 - University of Mississippi, Medicine, Jackson, Mississippi, 39213; 45 - Washington State University, Pullman, Washington, 99164; 46 - University of California, Los Angeles, Los Angeles, California, 90095;

47 - Brigham & Women's Hospital; 48 - Brigham & Women's Hospital, Medicine, Boston, Massachusetts, 02115; 49 - National Taiwan University, Taipei, 10617; 50 - Brigham & Women's Hospital, Division of Preventive Medicine, Boston, Massachusetts, 02215; 51 - University of Virginia, Charlottesville, Virginia, 22903; 52 - Lundquist Institute, Torrance, California, 90502; 53 - National Taiwan University, National Taiwan University Hospital, Taipei, 10617; 54 - Cleveland Clinic, Cleveland Clinic, Cleveland, Ohio, 44195; 55 - National Health Research Institute Taiwan, Miaoli County, 350; 56 - Broad Institute, Metabolomics Platform, Cambridge, Massachusetts, 02142; 57 - Cleveland Clinic, Immunity and Immunology, Cleveland, Ohio, 44195; 58 - University of Vermont, Burlington, Vermont, 05405; 59 - University of Mississippi, Population Health Science, Jackson, Mississippi, 39216; 60 - National Jewish Health, Denver, Colorado, 80206; 61 - Boston University, Biostatistics, Boston, Massachusetts, 02115; 62 - University of Texas Rio Grande Valley School of Medicine, Brownsville, Texas, 78520; 63 - University of Michigan, Internal Medicine, Ann Arbor, Michigan, 48109; 64 - Vitalant Research Institute, San Francisco, California, 94118; 65 - University of Illinois at Chicago, Chicago, Illinois, 60607; 66 - University of Chicago, Chicago, Illinois, 60637; 67 - Mayo Clinic, Health Quantitative Sciences Research, Rochester, Minnesota, 55905; 68 - Washington University in St Louis, Department of Medicine, Cardiovascular Division, St. Louis, Missouri, 63110; 69 - University of Texas Health at Houston, Human Genetics Center, Department of Epidemiology, Human Genetics, and Environmental Sciences, Houston, Texas, 77030; 70 - Vanderbilt University, Nashville, Tennessee, 37235; 71 - University of Cincinnati, Cincinnati, Ohio, 45220; 72 - University of North Carolina, Chapel Hill, North Carolina, 27599; 73 - Baylor College of Medicine Human Genome Sequencing Center, BCM, Houston, Texas, 77030; 74 - University of Texas Rio Grande Valley School of Medicine, Edinburg, Texas, 78539; 75 - University of Vermont, Pathology and Laboratory Medicine, Burlington, Vermont, 05405; 76 - Washington University in St Louis, Genetics, St Louis, Missouri, 63110; 77 - Brown University, Providence, Rhode Island, 02912; 78 - Harvard University, Channing Division of Network Medicine, Cambridge, Massachusetts, 02138; 79 - Massachusetts General Hospital, Boston, Massachusetts, 02114; 80 - Cleveland Clinic, Lerner Research Institute, Cleveland, Ohio, 44195; 81 - National Jewish Health, Center for Genes, Environment and Health, Denver, Colorado, 80206; 82 - University of Texas Health at Houston, Institute of Molecular Medicine McGovern Medical School, Houston, Texas, 77225; 83 - University of North Carolina, Epidemiology, Chapel Hill, North Carolina, 27599; 84 - Washington University in St Louis, St Louis, Missouri, 63130; 85 - Fred Hutchinson Cancer Research Center, Seattle, Washington, 98109; 86 - New York Genome Center, New York City, New York, 10013; 87 - Icahn School of Medicine at Mount Sinai, New York, New York, 10029; 88 - University of Pittsburgh, Pittsburgh, Pennsylvania; 89 - Beth Israel Deaconess Medical Center, Boston, Massachusetts, 02215; 90 - University of Pittsburgh, Pittsburgh, Pennsylvania, 15260; 91 - Boston Children's Hospital, Harvard Medical School, Department of Psychiatry, Boston, Massachusetts, 02115; 92 - University of Texas Rio Grande Valley School of Medicine, San Antonio, Texas, 78229; 93 - University of Colorado Anschutz Medical Campus, Aurora, Colorado, 80045; 94 - Mass General Brigham, Obstetrics and Gynecology, Boston, Massachusetts, 02115; 95 - Broad Institute, Broad Institute, Cambridge, Massachusetts, 02142; 96 - University of Mississippi, Cardiology, Jackson, Mississippi, 39216; 97 - University of Calgary, Medicine, Calgary; 98 - University of Maryland, Genetics, Philadelphia, Pennsylvania, 19104; 99 - Yale University, Department of Chronic Disease Epidemiology, New Haven, Connecticut, 06520; 100 - Tulane University, New Orleans, Louisiana, 70118; 101 - University

of Washington, Epidemiology, Seattle, Washington, 98195-9458; 102 - Wake Forest Baptist Health, Winston-Salem, North Carolina, 27157; 103 - Brigham & Women's Hospital, Channing Division of Network Medicine, Boston, Massachusetts, 02115; 104 - Regeneron Genetics Center, Boston, Massachusetts, 02115; 105 - University of Iowa, Iowa City, Iowa, 52242; 106 - National Health Research Institute Taiwan, Institute of Population Health Sciences, NHRI, Miaoli County, 350; 107 - Tri-Service General Hospital National Defense Medical Center; 108 - Blood Works Northwest, Seattle, Washington, 98104; 109 - Taichung Veterans General Hospital Taiwan, Taichung City, 407; 110 - Oklahoma State University Medical Center, Internal Medicine, DIvision of Endocrinology, Diabetes and Metabolism, Columbus, Ohio, 43210; 111 - National Heart, Lung, and Blood Institute, National Institutes of Health, NHLBI, Bethesda, Maryland, 20892; 112 - University of Washington, Medicine, Seattle, Washington, 98109; 113 - University of Michigan, Biostatistics, Ann Arbor, Michigan, 48109; 114 - Albert Einstein College of Medicine, New York, New York, 10461; 115 - University of California, San Francisco, San Francisco, California, 94118; 116 - Harvard University, Cambridge, Massachusetts, 02138; 117 - McGill University, MontrÃ©al, QC H3A 0G4; 118 - University of Colorado at Denver, Epidemiology, Aurora, Colorado, 80045; 119 - Blood Works Northwest, Medicine, Seattle, Washington, 98104; 120 - Loyola University, Public Health Sciences, Maywood, Illinois, 60153; 121 - Harvard School of Public Health, Biostats, Boston, Massachusetts, 02115; 122 - University of Colorado at Denver, Medicine, Aurora, Colorado, 80048; 123 - Boston University, University of Massachusetts Chan Medical School, Worcester, Massachusetts, 01655; 124 - Harvard School of Public Health, Dept of Biostatistics, Boston, Massachusetts, 02115; 125 - Brown University, Epidemiology and Medicine, Providence, Rhode Island, 02912; 126 - Duke University, Cardiology, Durham, North Carolina, 27708; 127 - Stanford University, Cardiovascular Institute, Stanford, California, 94305; 128 - Icahn School of Medicine at Mount Sinai, The Charles Bronfman Institute for Personalized Medicine, New York, New York, 10029; 129 - Boston University, Boston, Massachusetts, 02215; 130 - The Ohio State University, Division of Pulmonary, Critical Care and Sleep Medicine, Columbus, Ohio, 43210; 131 - Broad Institute, Harvard University, Massachusetts General Hospital; 132 - George Washington University, cardiology, Washington, District of Columbia, 20037; 133 - University of Alabama, University of Alabama at Birmingham, Birmingham, Alabama, 35487; 134 - Brown University, Epidemiology, Providence, Rhode Island, 02912; 135 - University of Washington, Genome Sciences, Seattle, Washington, 98195; 136 - RTI International; 137 - Massachusetts General Hospital, Medicine, Boston, Massachusetts, 02114; 138 - University of Arizona, Tucson, Arizona, 85721; 139 - Stanford University, Center For Sleep Sciences and Medicine, Palo Alto, California, 94304; 140 - National Institute of Child Health and Human Development, National Institutes of Health, Bethesda, Maryland, 20892; 141 - Oklahoma Medical Research Foundation, Genes and Human Disease, Oklahoma City, Oklahoma, 73104; 142 - Ministry of Health, Government of Samoa, Apia; 143 - Howard University, Washington, District of Columbia, 20059; 144 - University of Washington, Department of Genome Sciences, Seattle, Washington, 98195; 145 - University of Maryland, Balitmore, Maryland, 21201; 146 - University at Buffalo, Buffalo, New York, 14260; 147 - University of Pennsylvania, Division of Sleep Medicine/Department of Medicine, Philadelphia, Pennsylvania, 19104-3403; 148 - Stanford University, Stanford Cardiovascular Institute, Stanford, California, 94305; 149 - Wake Forest Baptist Health, Biochemistry, Winston-Salem, North Carolina, 27157; 150 - University of Minnesota, Minneapolis, Minnesota, 55455; 151 - RTI International, Biostatistics and Epidemiology Division, Research Triangle Park, North Carolina, 27709-2194; 152 - Boston

University, Department of Biostatistics, Boston, Massachusetts, 02118; 153 - Fred Hutchinson Cancer Research Center, Fred Hutch and UW, Seattle, Washington, 98109; 154 - Johns Hopkins University, Cardiology/Medicine, Baltimore, Maryland, 21218; 155 - University of Colorado at Denver, Medicine, Denver, Colorado, 80204; 156 - University of Colorado at Denver, CCPM, Denver, Colorado, 80045; 157 - University of North Carolina, Genetics, Chapel Hill, North Carolina, 27599; 158 - Northwestern University, Chicago, Illinois, 60208; 159 - New York Genome Center, New York Genome Center, New York City, New York, 10013; 160 - Fred Hutchinson Cancer Research Center, University of Washington, Seattle, Washington, 98109; 161 - Lutia I Puava Ae Mapu I Fagalele, Apia; 162 - University of Ottawa, Sleep Research Unit, University of Ottawa Institute for Mental Health Research, Ottawa, ON K1Z 7K4; 163 - Vanderbilt University, Medicine, Pharmacology, Biomedicla Informatics, Nashville, Tennessee, 37235; 164 - Lundquist Institute, Pediatrics, Torrance, California, 90502; 165 - University of Washington, Seattle, Washington, 98104; 166 - Universidade de Sao Paulo, Faculdade de Medicina, Sao Paulo, 01310000; 167 - Columbia University, New York, New York, 10027; 168 - University of Maryland, Pathology, Seattle, Washington, 98195; 169 - Lundquist Institute, TGPS, Torrance, California, 90502; 170 - Harvard University, Division of Hematology/Oncology, Boston, Massachusetts, 02115; 171 - Harvard Medical School, Genetics, Boston, Massachusetts, 02115; 172 - Harvard Medical School, Boston, Massachusetts, 02115; 173 - UniversitÃ© Laval, Quebec City, G1V 0A6; 174 - Emory University, Pediatrics, Atlanta, Georgia, 30307; 175 - Emory University, Human Genetics, Atlanta, Georgia, 30322; 176 - Vanderbilt University, Medicine/Cardiology, Nashville, Tennessee, 37235; 177 - UMass Memorial Medical Center, Worcester, Massachusetts, 01655; 178 - University of Saskatchewan, Saskatoon, SK S7N 5C9; 179 - University of Michigan; 180 - University of Washington, Epidemiology, Seattle, Washington, 98195; 181 - Wake Forest Baptist Health, Biostatistical Sciences, Winston-Salem, North Carolina, 27157; 182 - Stanford University, Genetics, Stanford, California, 94305; 183 - University of Colorado at Denver, Genomic Cardiology, Aurora, Colorado, 80045; 184 - Brigham & Women's Hospital, Channing Department of Medicine, Boston, Massachusetts, 02115; 185 - Lundquist Institute, Institute for Translational Genomics and Populations Sciences, Torrance, California, 90502; 186 - University of Washington, University of Washington, Department of Genome Sciences, Seattle, Washington, 98195; 187 - Fred Hutchinson Cancer Research Center, Cancer Prevention Division of Public Health Sciences, Seattle, Washington, 98109; 188 - University of Pennsylvania, Genetics, Philadelphia, Pennsylvania, 19104; 189 - University of Alabama, Biostatistics, Birmingham, Alabama, 35487; 190 - University of Washington, Department of Biostatistics, Seattle, Washington, 98195; 191 - University of Vermont, Pathology & Laboratory Medicine, Burlington, Vermont, 05405; 192 - University of Southern California, USC Methylation Characterization Center, University of Southern California, California, 90033; 193 - Brigham & Women's Hospital, Mass General Brigham, Boston, Massachusetts, 02115; 194 - University of Pittsburgh, Department of Human Genetics, Pittsburgh, Pennsylvania, 15260; 195 - Brigham & Women's Hospital, Channing Division of Network Medicine, Department of Medicine, Boston, Massachusetts, 02115; 196 - Indiana University, Epidemiology, Indianapolis, Indiana, 46202; 197 - Henry Ford Health System, Detroit, Michigan, 48202; 198 - Case Western Reserve University; 199 - Beth Israel Deaconess Medical Center, Cardiology, Cambridge, Massachusetts, 02139; 200 - Henry Ford Health System, Department of Medicine, Detroit, Michigan, 48202; 201 - University of Pittsburgh, Medicine, Pittsburgh, Pennsylvania, 15260; 202 - University of Michigan, Department of Epidemiology, Ann Arbor, Michigan, 48109; 203 - Case Western Reserve

University, Department of Population and Quantitative Health Sciences, Cleveland, Ohio, 44106; 204 - University of California, San Francisco, Medicine, San Francisco, California, 94143

## Supplementary References


1. Sudlow, C. *et al.* UK biobank: an open access resource for identifying the causes of a wide range of complex diseases of middle and old age. *PLoS Med.* **12,** e1001779 (2015).
2. Constantinescu, A.-E. *et al.* A framework for research into continental ancestry groups of the UK Biobank. *Hum. Genomics* **16,** 3 (2022).
3. Chiu, A. M., Molloy, E. K., Tan, Z., Talwalkar, A. & Sankararaman, S. Inferring population structure in biobank-scale genomic data. *Am. J. Hum. Genet.* **109,** 727–737 (2022).
4. Bycroft, C. *et al.* The UK Biobank resource with deep phenotyping and genomic data. *Nature* **562,** 203–209 (2018).
5. Choi, S. W. & O'Reilly, P. F. PRSice-2: Polygenic Risk Score software for biobank-scale data. *Gigascience* **8,** (2019).
6. Alexander, D. H., Novembre, J. & Lange, K. Fast model-based estimation of ancestry in unrelated individuals. *Genome Res.* **19,** 1655–1664 (2009).
7. Maples, B. K., Gravel, S., Kenny, E. E. & Bustamante, C. D. RFMix: a discriminative modeling approach for rapid and robust local-ancestry inference. *Am. J. Hum. Genet.* **93,** 278–288 (2013).
8. All of Us Research Program Investigators *et al.* The "All of Us" Research Program. *N. Engl. J. Med.* **381,** 668–676 (2019).
9. Yu, S. *et al.* Toward high-throughput phenotyping: unbiased automated feature extraction and selection from knowledge sources. *J. Am. Med. Inform. Assoc.* **22,** 993–1000 (2015).
10. The ARIC Investigators. The Atherosclerosis Risk in Communities (ARIC) Study: design and objectives. . *Am. J. Epidemiol.* **129,** 687–702 (1989).
11. Wright, J. D. *et al.* The ARIC (atherosclerosis risk in communities) study: JACC focus seminar 3/8. *J. Am. Coll. Cardiol.* **77,** 2939–2959 (2021).
12. Friedman, G. D. *et al.* CARDIA: study design, recruitment, and some characteristics of the examined subjects. *J. Clin. Epidemiol.* **41,** 1105–1116 (1988).
13. Redline, S. *et al.* The familial aggregation of obstructive sleep apnea. *Am. J. Respir. Crit. Care Med.* **151,** 682–687 (1995).
14. Regan, E. A. *et al.* Genetic epidemiology of COPD (COPDGene) study design. *COPD* **7,** 32–43 (2010).
15. Dawber, T. R., Kannel, W. B. & Lyell, L. P. An approach to longitudinal studies in a community: the Framingham Study. *Ann. N. Y. Acad. Sci.* **107,** 539–556 (1963).
16. Splansky, G. L. *et al.* The Third Generation Cohort of the National Heart, Lung, and Blood Institute's Framingham Heart Study: design, recruitment, and initial examination. *Am. J. Epidemiol.* **165,** 1328–1335 (2007).
17. Kannel, W. B., Feinleib, M., McNamara, P. M., Garrison, R. J. & Castelli, W. P. An investigation of coronary heart disease in families. The Framingham offspring study. *Am. J. Epidemiol.* **110,** 281–290 (1979).
18. GenSalt Collaborative Research Group. GenSalt: rationale, design, methods and baseline

characteristics of study participants. (2007).
19. Perloff, D. *et al.* Human blood pressure determination by sphygmomanometry. *Circulation* **88,** 2460–2470 (1993).
20. Lavange, L. M. *et al.* Sample design and cohort selection in the Hispanic Community Health Study/Study of Latinos. *Ann Epidemiol* **20,** 642–649 (2010).
21. Sorlie, P. D. *et al.* Design and implementation of the Hispanic Community Health Study/Study of Latinos. *Ann Epidemiol* **20,** 629–641 (2010).
22. Wyatt, S. B. *et al.* A community-driven model of research participation: the Jackson Heart Study Participant Recruitment and Retention Study. *Ethn Dis* **13,** 438–455 (2003).
23. Taylor, H. A. *et al.* Toward resolution of cardiovascular health disparities in African Americans: design and methods of the Jackson Heart Study. *Ethn Dis* **15,** S6-4 (2005).
24. Bild, D. E. *et al.* Multi-Ethnic Study of Atherosclerosis: objectives and design. *Am. J. Epidemiol.* **156,** 871–881 (2002).
