# Supplementary material for: Evaluating the use of blood pressure polygenic risk scores across race/ethnic background groups
Source: Nat Commun. 2023 Jun 2;14:3202. doi: 10.1038/s41467-023-38990-9 (PMC10238525; doi:10.1038/s41467-023-38990-9)
Supplement: Supplementary file 3 — Description of Additional Supplementary Files [file 41467_2023_38990_MOESM3_ESM.pdf]

Description of Additional Supplementary Files

File Name: Supplementary Data 1
Description: Data behind Figure 2

File Name: Supplementary Data 2
Description: Data behind Figure 3

File Name: Supplementary Data 3
Description: Data behind Figure 4

File Name: Supplementary Data 4
Description: Data behind Figure 5
