# Supplementary material for: Evaluating the use of blood pressure polygenic risk scores across race/ethnic background groups
Source: Nat Commun. 2023 Jun 2;14:3202. doi: 10.1038/s41467-023-38990-9 (PMC10238525; doi:10.1038/s41467-023-38990-9)
Supplement: Supplementary file 8 — Reporting Summary [file 41467_2023_38990_MOESM8_ESM.pdf]

nature portfolio

Corresponding author(s): Dr. Tamar Sofer

Last updated by author(s): May 8, 2023

# Reporting Summary

Nature Portfolio wishes to improve the reproducibility of the work that we publish. This form provides structure for consistency and transparency in reporting. For further information on Nature Portfolio policies, see our Editorial Policies and the Editorial Policy Checklist.

## Statistics

For all statistical analyses, confirm that the following items are present in the figure legend, table legend, main text, or Methods section.

| n/a | Confirmed | |
|---|---|---|
| ☐ | ☒ | The exact sample size (*n*) for each experimental group/condition, given as a discrete number and unit of measurement |
| ☒ | ☐ | A statement on whether measurements were taken from distinct samples or whether the same sample was measured repeatedly |
| ☐ | ☒ | The statistical test(s) used AND whether they are one- or two-sided<br>*Only common tests should be described solely by name; describe more complex techniques in the Methods section.* |
| ☐ | ☒ | A description of all covariates tested |
| ☐ | ☒ | A description of any assumptions or corrections, such as tests of normality and adjustment for multiple comparisons |
| ☐ | ☒ | A full description of the statistical parameters including central tendency (e.g. means) or other basic estimates (e.g. regression coefficient) AND variation (e.g. standard deviation) or associated estimates of uncertainty (e.g. confidence intervals) |
| ☐ | ☒ | For null hypothesis testing, the test statistic (e.g. *F*, *t*, *r*) with confidence intervals, effect sizes, degrees of freedom and *P* value noted<br>*Give P values as exact values whenever suitable.* |
| ☒ | ☐ | For Bayesian analysis, information on the choice of priors and Markov chain Monte Carlo settings |
| ☒ | ☐ | For hierarchical and complex designs, identification of the appropriate level for tests and full reporting of outcomes |
| ☐ | ☒ | Estimates of effect sizes (e.g. Cohen's *d*, Pearson's *r*), indicating how they were calculated |

*Our web collection on statistics for biologists contains articles on many of the points above.*

## Software and code

Policy information about availability of computer code

| | |
|---|---|
| Data collection | No software was used. |
| Data analysis | The following softwares were used: R/4.0.2, Python 2.7, PRSice 2.3.1.e , PLINK v1.9, bigsnpr v1.9.11,PRS-CSx v1.0.0, GENESIS R Packages v2.16.1, pROC R package v1.16.2. Costume code is available on the GitHub repository https://github.com/nkurniansyah/BP_PRS, and the Zenodo repository https://doi.org/10.5281/zenodo.7908793. |

For manuscripts utilizing custom algorithms or software that are central to the research but not yet described in published literature, software must be made available to editors and reviewers. We strongly encourage code deposition in a community repository (e.g. GitHub). See the Nature Portfolio guidelines for submitting code & software for further information.

## Data

Policy information about availability of data

All manuscripts must include a data availability statement. This statement should provide the following information, where applicable:

- Accession codes, unique identifiers, or web links for publicly available datasets
- A description of any restrictions on data availability
- For clinical datasets or third party data, please ensure that the statement adheres to our policy

TOPMed freeze 8 WGS data and harmonized BP phenotypes are available by application to dbGaP according to the study specific accessions:
Amish: “phs000956 [https://www.ncbi.nlm.nih.gov/projects/gap/cgi-bin/study.cgi?study_id=phs000956.v1.p1]”, ARIC: “phs001211 [ https://www.ncbi.nlm.nih.gov/

projects/gap/cgi-bin/study.cgi?study_id=phs001211.v4.p3]", BioMe: "phs001644 [https://www.ncbi.nlm.nih.gov/projects/gap/cgi-bin/study.cgi?study_id=phs001644.v2.p2]", CARDIA: "phs001612 [https://www.ncbi.nlm.nih.gov/projects/gap/cgi-bin/study.cgi?study_id=phs001612.v1.p1]", CFS: "phs000954 [https://www.ncbi.nlm.nih.gov/projects/gap/cgi-bin/study.cgi?study_id=phs000954.v4.p2]", CHS: "phs001368 [https://www.ncbi.nlm.nih.gov/projects/gap/cgi-bin/study.cgi?study_id=phs001368.v3.p2]", COPDGene: "phs000951 [https://www.ncbi.nlm.nih.gov/projects/gap/cgi-bin/study.cgi?study_id=phs000951.v5.p5]", FHS: "phs000974 [https://www.ncbi.nlm.nih.gov/projects/gap/cgi-bin/study.cgi?study_id=phs000974.v4.p3]", GENOA: "phs001345 [https://www.ncbi.nlm.nih.gov/projects/gap/cgi-bin/study.cgi?study_id=phs001345.v3.p1]", GenSalt: "phs001217 [https://www.ncbi.nlm.nih.gov/projects/gap/cgi-bin/study.cgi?study_id=phs001217.v3.p1]", HCHS/SOL: "phs001395 [https://www.ncbi.nlm.nih.gov/projects/gap/cgi-bin/study.cgi?study_id=phs001395.v2.p1, JHS: "phs000964 [https://www.ncbi.nlm.nih.gov/projects/gap/cgi-bin/study.cgi?study_id=phs000964.v5.p1]", MESA: "phs001211 [https://www.ncbi.nlm.nih.gov/projects/gap/cgi-bin/study.cgi?study_id=phs001211.v4.p3]", THRV: "phs001387 [https://www.ncbi.nlm.nih.gov/projects/gap/cgi-bin/study.cgi?study_id=phs001387.v3.p1]", WHI: "phs001237 [https://www.ncbi.nlm.nih.gov/projects/gap/cgi-bin/study.cgi?study_id=phs001237.v3.p1]". Summary statistics from MVP BP GWAS are available from dbGaP by application to study accession "phs001672 [https://www.ncbi.nlm.nih.gov/projects/gap/cgi-bin/study.cgi?study_id=phs001672.v4.p1]". The summary statistics from the UKBB+ICBP BP GWAS are available at https://grasp.nhlbi.nih.gov/FullResults.aspx. The summary statistics from the COGENT BP GWAS are available at https://tarheels.live/cogentkidney/main/gwas-cogent-bp/. The summary statistics from the BBJ BP GWAS are available at http://jenger.riken.jp/en/. MGB Biobank genotyping and phenotypic data are available to Mass General Brigham investigators with required approval from the Mass General Brigham Institutional Review board (IRB).
Data from the NIH All of Us study are available via institutional data access for researchers who meet the criteria for access to confidential data. To register as a researcher with All of Us, researchers may use the following URL and complete the laid out steps: https://www.researchallofus.org/register/. Researchers can contact All of Us Researcher Workbench Support at support@researchallofus.org. Data needed to construct the selected BP PRSs generated in this study are publicly available on the Zenodo repository https://doi.org/10.5281/zenodo.7908793, and include variants, alleles, and weights for each of the PRS based on GWAS of SBP and DBP, mean and SD computed based on the TOPMed-BP dataset, and code to generate the PRS from plink files using PLINK v1.9. Source data for figures 2-5 are provided with this paper.

## Human research participants

Policy information about studies involving human research participants and Sex and Gender in Research.

Reporting on sex and gender

Our data included self-reported males and females. For TOPMed data, self-reported males and females also passed chromosomal checks requiring two x chromosomes for females and one x and one y chromosome for males. For All of Us, we used sex assigned at birth.

Population characteristics

Individuals are adults from multiple cohort and biobank studies. Population characteristics are reported in Supplementary Tables 2-5

Recruitment

Multiple studies participated in the analyses in this manuscript. All the detail can be found in supplementary material in the study description section.

Ethics oversight

Ethics statements are provided in the Supplementary Materials for each study.

Amish:
All study protocols were approved by the institutional review board at the University of Maryland Baltimore. Informed consent was obtained from each study participant.
ARIC:
The ARIC study has been approved by Institutional Review Boards (IRB) at all participating institutions: University of North Carolina at Chapel Hill IRB, Johns Hopkins University IRB, University of Minnesota IRB, and University of Mississippi Medical Center IRB. Study participants provided written informed consent at all study visits.
BioMe:
The BioMe cohort was approved by the Institutional Review Board at the Icahn School of Medicine at Mount Sinai. All BioMe participants provided written, informed consent for genomic data sharing.
CARDIA:
All CARDIA participants provided informed consent, and the study was approved by the Institutional Review Boards of the University of Alabama at Birmingham and the University of Texas Health Science Center at Houston.
CFS:
Cleveland Family Study was approved by the Institutional Review Board (IRB) of Case Western Reserve University and Mass General Brigham (formerly Partners HealthCare). Written informed consent was obtained from all participants.
CHS:
All CHS participants provided informed consent, and the study was approved by the Institutional Review Board [or ethics review committee] of University Washington.
COPDGene
All COPDGene participants provided written informed consent, and the study was approved by the Institutional Review Boards of the participating clinical centers.
FHS:
The Framingham Heart Study was approved by the Institutional Review Board of the Boston University Medical Center. All study participants provided written informed consent.
GENOA:
Written informed consent was obtained from all subjects and approval was granted by participating institutional review boards (University of Michigan, University of Mississippi Medical Center, and Mayo Clinic).
GenSalt:
All subjects provided informed consent and the GenSalt study was approved by the Institutional Review Board (IRB) of all participating institutes in the US and China.
HCHS/SOL:
This study was approved by the institutional review boards (IRBs) at each field center, where all participants gave written informed consent, and by the Non-Biomedical IRB at the University of North Carolina at Chapel Hill, to the HCHS/SOL Data

Coordinating Center. All IRBs approving the study are: Non-Biomedical IRB at the University of North Carolina at Chapel Hill. Chapel Hill, NC; Einstein IRB at the Albert Einstein College of Medicine of Yeshiva University. Bronx, NY; IRB at Office for the Protection of Research Subjects (OPRS), University of Illinois at Chicago. Chicago, IL; Human Subject Research Office, University of Miami. Miami, FL; Institutional Review Board of San Diego State University. San Diego, CA.
JHS:
The JHS study was approved by Jackson State University, Tougaloo College, and the University of Mississippi Medical Center IRBs, and all participants provided written informed consent
WHI:
All WHI participants provided informed consent and the study was approved by the Institutional Review Board (IRB) of the Fred Hutchinson Cancer Research Center.
MESA:
All MESA participants provided written informed consent, and the study was approved by the Institutional Review Boards at The Lundquist Institute (formerly Los Angeles BioMedical Research Institute) at Harbor-UCLA Medical Center, University of Washington, Wake Forest School of Medicine, Northwestern University, University of Minnesota, Columbia University, and Johns Hopkins University.
THRV:
All THRV participants provided informed consent, and the study was approved by the Institutional Review Board at The Lundquist Institute (formerly Los Angeles BioMedical Research Institute, or LA BioMed) at Harbor-UCLA Medical Center, and at Washington University in St. Louis.
UKBB:
The National Health Service National Research Ethics Service (ref. 11/NW/0382) gave approval for the study.
MGB Biobank:
All Biobank subjects have provided their consent to join the MGB Biobank, which includes agreeing to provide a blood sample linked to the electronic medical record. Subjects also agree to be recontacted by the Partners Biobank staff as needed.
All of Us:
The All of Us research program was approved by a single IRB, the "All of Us IRB", which is charged with reviewing the protocol, informed consent, and other participant-facing materials for the All of Us Research Program. The IRB follows the regulations and guidance of the Office for Human Research Protections for all studies, ensuring that the rights and welfare of research participants are overseen and protected uniformly. More information is provided online https://allofus.nih.gov/about/who-we-are/institutional-review-board-irb-of-all-of-us-research-program and in the All of Us design paper (5)

Note that full information on the approval of the study protocol must also be provided in the manuscript.

# Field-specific reporting

Please select the one below that is the best fit for your research. If you are not sure, read the appropriate sections before making your selection.

☒ Life sciences ☐ Behavioural & social sciences ☐ Ecological, evolutionary & environmental sciences

For a reference copy of the document with all sections, see nature.com/documents/nr-reporting-summary-flat.pdf

# Life sciences study design

All studies must disclose on these points even when the disclosure is negative.

| | |
|---|---|
| Sample size | The TOPMed aggregated dataset included 62,491<br>The UKB dataset included 8,643<br>The MGB Biobank included 36,434<br>The All of Us data set included 88,521<br>The sample sizes were selected based on being the available samples. We knew that these sample sizes are sufficient based on previous work with PRS for BP phenotypes, that used smaller sample sizes and detected associations. |
| Data exclusions | In analysis COPDGene in TOPMed data, we only use non-COPD cases .<br>In analysis of All of Us data, as well as of MGB dataset, we excluded unrelated individuals. In All of Us, we included only self-reported non-Hispanic Asian, non-Hispanic White, non-Hispanic Black, and Hispanic/Latino individuals. We excluded individuals who were not male or female in their sex assigned at birth. |
| Replication | Polygenic risk scores were constructed based on TOPMed and MGB biobank, and were validated in the independent All of Us study. |
| Randomization | There was no randomization, this is an observational study. |
| Blinding | There was no blinding, this is an observational study (no treatment). |

# Reporting for specific materials, systems and methods

We require information from authors about some types of materials, experimental systems and methods used in many studies. Here, indicate whether each material, system or method listed is relevant to your study. If you are not sure if a list item applies to your research, read the appropriate section before selecting a response.

## Materials & experimental systems

| n/a | Involved in the study |
|---|---|
| ☒ | ☐ Antibodies |
| ☒ | ☐ Eukaryotic cell lines |
| ☒ | ☐ Palaeontology and archaeology |
| ☒ | ☐ Animals and other organisms |
| ☒ | ☐ Clinical data |
| ☒ | ☐ Dual use research of concern |

## Methods

| n/a | Involved in the study |
|---|---|
| ☒ | ☐ ChIP-seq |
| ☒ | ☐ Flow cytometry |
| ☒ | ☐ MRI-based neuroimaging |
